# Tree functional traits, forest biomass, and tree species diversity interact with site properties to drive forest soil carbon

**DOI:** 10.1038/s41467-022-28748-0

**Published:** 2022-03-01

**Authors:** Laurent Augusto, Antra Boča

**Affiliations:** 1 INRAE, Bordeaux Sciences Agro, UMR 1391 ISPA, 33882 Villenave d’Ornon, France; 2 Latvia University of Life Sciences and Technologies, Lielā iela 2, Jelgava, LV-3001 Latvia

**Keywords:** Carbon cycle, Forest ecology, Ecosystem ecology

## Abstract

Forests constitute important ecosystems in the global carbon cycle. However, how trees and environmental conditions interact to determine the amount of organic carbon stored in forest soils is a hotly debated subject. In particular, how tree species influence soil organic carbon (SOC) remains unclear. Based on a global compilation of data, we show that functional traits of trees and forest standing biomass explain half of the local variability in forest SOC. The effects of functional traits on SOC depended on the climatic and soil conditions with the strongest effect observed under boreal climate and on acidic, poor, coarse-textured soils. Mixing tree species in forests also favours the storage of SOC, provided that a biomass over-yielding occurs in mixed forests. We propose that the forest carbon sink can be optimised by (i) increasing standing biomass, (ii) increasing forest species richness, and (iii) choosing forest composition based on tree functional traits according to the local conditions.

## Abstract (web summary)

Forests constitute important ecosystems in the global carbon cycle. This study investigates how tree species influence soil organic carbon using a global dataset, showing the importance of tree functional traits and forest standing biomass to optimise forest carbon sink.

## Introduction

Forests have been identified as a major leverage for climate change mitigation because of their capacity to fix atmospheric CO_2_ and transform it as organic carbon, in biomass and then in soils^1,2^. On average, trees and soils store similar amounts of carbon in forest ecosystems^2,3^. Nevertheless, standing tree biomass reaches its limit in various regions of the world^4,5^ and, contrary to soils, the carbon pool stored in standing biomass is highly vulnerable to many hazards like windthrow, drought, wildfire, pest, and disease (e.g.,^6,7^). In a context of global change-induced disturbances, storing carbon in forest soils thus appears as an approach less vulnerable than storing in the biomass of standing trees^8^.

Massive planting of trees has been proposed as an efficient method to improve the carbon balance of lands^9,10^. The quantitative impact of large scale afforestations is however debatable^9,11^, and the factors and management approaches affecting carbon sequestration in forests on a large scale have yet to be determined^3^. While the debate about planting more than a trillion trees has resulted in actions taken by international companies (e.g. www.trilliontreecampaign.org)^10^, the influence of forest tree composition on soil organic carbon (SOC) is often not part of the debate as it is still elusive despite decades of research^3,12,13^ even though forest composition strongly influences ecosystem functioning^14,15^. Apart from the uppermost soil layer known as the *forest floor* (which is the accumulation of decomposing dead foliage), no general and consistent trend has been isolated for non-nitrogen-fixing species^3,12,16–18^. Consequently, this study aimed at the identification of tree species and their characteristics that enlarge SOC pools and increase SOC stability. Due to the high number of extant tree species, and minimal recurrence of individual species among regions of the world, we did not compare species directly but quantified the influence on SOC of the main plant traits of the tree species (e.g., foliage nitrogen content).

Based on published findings, we formulated three hypotheses. First, because the properties of plant debris (such as litterfall and dead roots) strongly influence both their initial decomposition^19–23^ and the subsequent stabilisation of organic carbon in soil^24–28^, we hypothesised [H1] that plant traits drive SOC, in pool size and in stability. In particular, we anticipated that gymnosperm species increase SOC accumulation whereas angiosperm species favour SOC stabilisation^12^. We further hypothesised [H2] that the imprint of tree species traits, and standing biomass, on SOC is in interaction with site properties^12,29,30^ because the way organic matter is accumulated –or not– depends on processes (i.e., decomposition and mineralisation versus microbial processing, chemical binding, and soil aggregation) whose relative importance varies with local conditions^1^. For instance, SOC stabilisation through microbial processing of organic matter is favoured by soil nutrient availability^31–33^, implying that the influence of plant nutrient content (notably in nitrogen (N) and phosphorus (P)) is highest in nutrient poor soils^31,34^. We assumed that climate (temperature and water availability), soil fertility, soil texture (sand content and clay content), and soil pH are the site properties that are the most likely to affect the influence of plant traits on SOC^1,12,14,35–37^. Finally, we hypothesised that forest standing biomass [H3a] and tree diversity [H3b] increase the SOC pool size, respectively directly and indirectly. Indeed, high levels of standing biomass imply large fluxes of plant necromass, which in turn can increase the quantity of organic matter in soils^1,38–41^. Similarly, because the so-called mixed forests (i.e., forests composed of several tree species) have on average a higher growth rate than mono-specific forests^42–45^, we expected that they store more SOC through their positive effect on standing biomass.

To address these hypotheses, we compiled, aggregated and harmonised published data on forest SOC and tree traits (see “Methods”), and conducted a global analysis based on 454 mature and mono-specific forest stands clustered in 136 sites worldwide. The stands at each site were in comparable conditions, enabling us to compare the influence of 178 different tree species, from 35 families, on SOC pools. Data about mixed stands or SOC stability were extracted from original publications for 29 and 30 sites, respectively.

## Results and discussion

At the global scale, the SOC stocks are strongly influenced by climate, soil properties, and nitrogen atmospheric deposition (Supplementary Fig. S1), as already reported in the literature^46–48^. Because of the strong imprint of the environment on SOC when comparing different sites, and because our objective was to quantify the influence of plant traits of different tree species growing in the same conditions, we normalised SOC values relative to the mean C pool of a site before further analysis. Similarly, the functional trait values and the properties of the forest stands (e.g., aboveground biomass) were normalised per site to take into account the site to site variations in tree species composition. This means that the results reported in the present study showed the effect of forest properties (plant trait values and stand biomass) after the influence of the environment was removed. Positive, null, or negative normalised values indicated absolute values that were respectively above, equal, or below the site mean value (see “Data handling and normalisation” in Methods).

Our results showed that the SOC pool size (forest floor plus mineral soil layers) was negatively correlated with many plant functional traits, such as leaf nitrogen (N) content, specific leaf area (SLA), specific root length (SRL), and wood density, with the exception of leaf dry matter content (LDMC) that had a positive effect (Supplementary Fig. S2). Having numerous plant traits that are related to a single response variable is not surprising since all these traits form the well-known *Plant Economics Spectrum* (PES;^49,50^). The PES describes how all plant functions are dependent on each-other, implying trade-offs and high levels of correlation among traits^51^, as observed in our study (Supplementary Fig. S3). Observed at the plant scale, these trade-offs among traits are also strong at the organ scale, defining the *Leaf Economics Spectrum*^52,53^ and the *Root Economics Spectrum*^54,55^. Interestingly, while plant traits forming the PES had a strong effect on SOC (| r | ≥ 0.25; see below), the phylogenetic distance among tree species, which represents life history characteristics, only poorly explained the differences in SOC (Supplementary Fig. S4). This lack of consistency was not totally surprising because, whereas the phylogenetic distance may explain the values distribution of a trait^56,57^, it also often poorly correlates with important traits^58^ and more generally with ecological difference^59^.

**Fig. 1:**
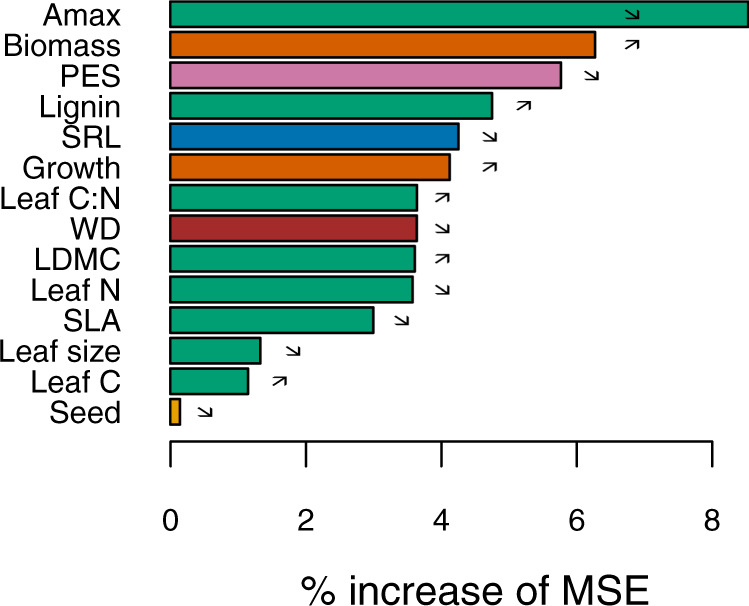
Main variables explaining the SOC pools at the local scale. The SOC pool was modelled using relative values, normalised to site conditions. The predictors were: (i) leaf traits, in green: maximum photosynthetic capacity (A_max_), C content, N content, C:N ratio, lignin content, leaf dry matter content (LDMC), leaf size and specific leaf area (SLA); (ii) other plant traits [seed mass (in yellow), wood density (WD; in brown), specific root length (SRL; in blue)]; (iii) the index score of the Plant Economics Spectrum (PES; in violet); and (iv) stand biomass dynamics, in dark orange (standing biomass; tree species growth). The influence of the variables was assessed using the percentage of increase of MSE from the Random Forest approach (see “Methods”). Arrows indicate positive (↗) or negative (↘) effects of the predictors on SOC. Source data are provided as a Source Data files.

**Fig. 2:**
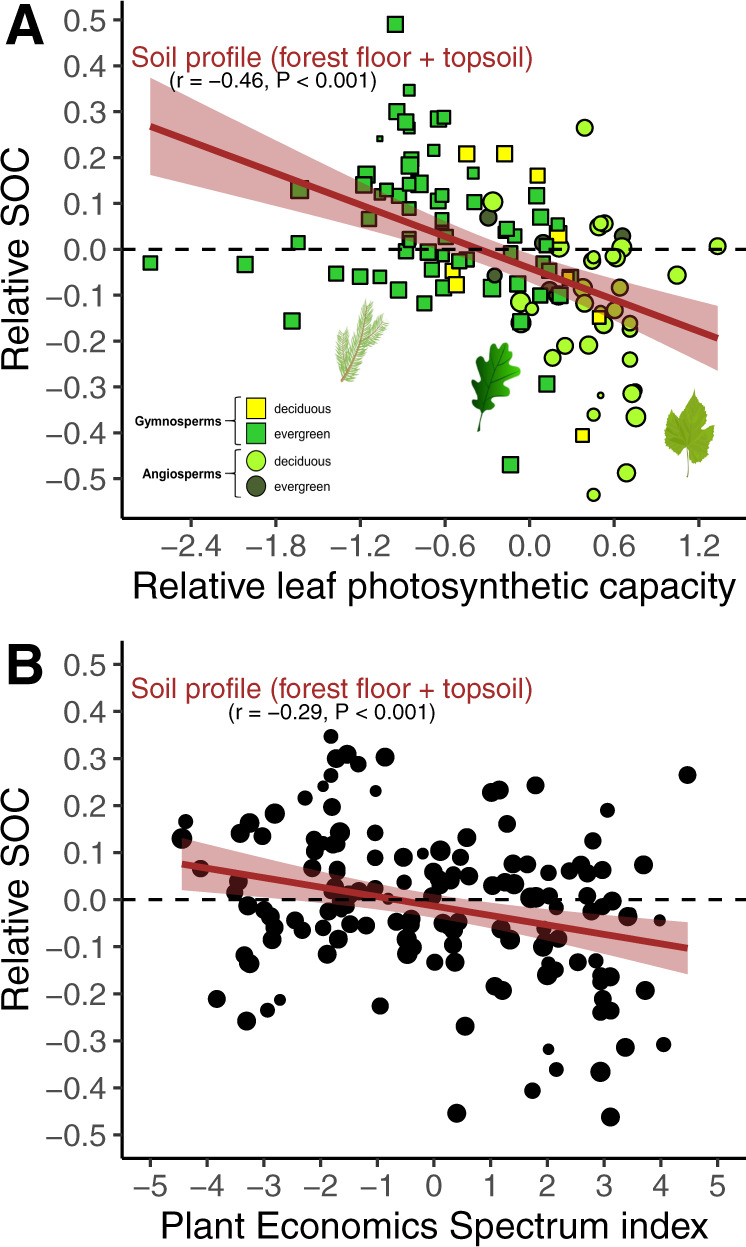
Global influence of plant functional traits on SOC pool. Relationship between photosynthetic capacity of tree species (A_max_) and SOC pool (**A**); relationship between the index score of the Plant Economics Spectrum (PES) and SOC pool (**B**). Values are normalised and the symbol size is proportional to data reliability (see “Methods”), which was taken into account as a weighting factor in the regression. A linear regression was fitted (level of confidence of the error band = 0.95). Source data are provided as a Source Data files.

Even though LDMC, SLA, and SRL had a high influence on SOC pools (Supplementary Table S1), we built our model using firstly the leaf photosynthetic maximum capacity (A_max_) as it is an integrative trait of plant functioning^51^, and because it is the best predictor of SOC (Fig. 1). A model based on A_max_ (Fig. 2A), and to a lower extent forest standing biomass, explained half (48.5%; adjusted r^2^) of the variance associated with SOC pool size (48.5% = 37.0% for A_max_ + 11.5% for biomass; Supplementary Figs. S5A and S6; Supplementary Table S2), supporting our hypothesis H1 (see below more results about biomass). This model showed a satisfactory level of consistency using an independent dataset (Supplementary Fig. S5B). The influence of plant A_max_ on SOC was particularly clear in the forest floor (Supplementary Fig. S7A), but was also significant in the mineral soil (Supplementary Fig. S7B; Supplementary Table S1). Using an integrative index of the PES (based on the PCA approach with imputation; see Methods for more details) confirmed that not only tree A_max_ influenced SOC pools, but the whole combination of functional traits and species characteristics (Fig. 2B). On average, tree species with low values of leaf A_max_, N and P content, wood density, SLA and SRL, but high value of LDMC, have large SOC pools. This combination of traits is typical of tree species with a conservative resource strategy (hereafter referred to as *conservative species*), such as many ectomycorrhizal gymnosperms (Supplementary Table S3;^60^), in contrast to arbuscular mycorrhizal angiosperms with an acquisitive resource strategy (hereafter: *acquisitive species*). We found that gymnosperm species store on average more SOC than angiosperm species, which could be attributed to them being generally conservative (Supplementary Fig. S8).

**Fig. 3:**
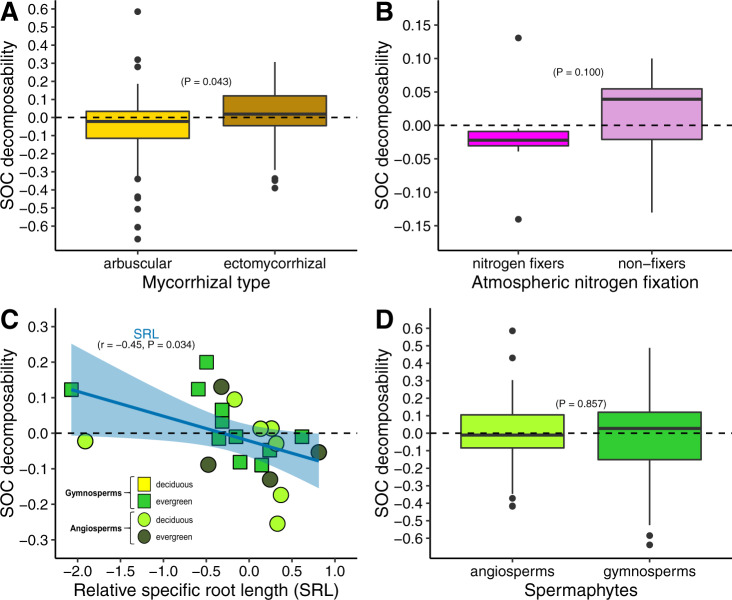
SOC decomposability as a function of plant functional types and specific root length. The values show the SOC decomposability, which is the opposite of SOC stability. Values are normalised (see “Methods”). Number of values: *n* = 49 & 52 (arbuscular versus ectomycorrhizal), *n* = 8 & 14 (fixers versus non-fixers), *n* = 28 & 31 (angiosperms versus gymnosperms). **A**, **B**, **D**: boxplots represent the median, the first and third quartiles, and 1.5× the inter-quartile range; significant differences tested with pairwise *t* test or Wilcoxon test (two-sided), depending on data structure. **C** a linear regression was fitted (level of confidence of the error band = 0.95). Source data are provided as a Source Data files.

In addition to total SOC content, we studied several metrics of SOC decomposability. Even if they do not take into account all process involved in organic matter preservation such as leaching of dissolved organic carbon, these metrics enabled us to evaluate the effect of tree species on SOC stability. Our results indicated that plant functional types (e.g., mycorrhizal type) and tree traits influenced SOC stability with arbuscular mycorrhizal species, nitrogen-fixing species, and species with high SRL favouring SOC stability (Fig. 3A–C). The absence of clear effect of the spermaphyte type (Fig. 3D) may be explained by the fact that both mycorrhizal types could be found in angiosperms or gymnosperms, and that SRL values of angiosperms overlap with gymnosperms values^61^. The influence of some plant functional types on SOC stability supports the idea that species with nutrient-rich litters (i.e., arbuscular mycorrhizal species and N-fixers)^60^ may favour chemical binding to soil minerals, soil aggregation, and microbial processing of organic matter^12,25,62^, which in turn increases SOC stability^12,15,19,26^. But, because in our global dataset the SOC pool is on average smaller under these species as compared with under conservative species, it suggests that the influence of tree species on the amount of carbon storage in soils is generally more driven by accumulation of organic matter inherently recalcitrant to decomposition^23^ than by SOC long-term stabilisation through chemical binding to soil minerals, or soil aggregation^1^.

**Fig. 4:**
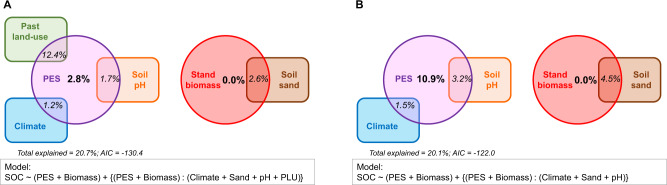
Modulation of the imprint of tree species on SOC by site properties. The Euler diagrams present the relative importance of factors (and some of their interactions) in explaining the SOC pool. The model used an integrative index of the Plant Economics Spectrum (PES) as predictor (see Methods), which enabled to include a large dataset of sites worldwide but at the expense of the level of variance explained (see Supplementary Table S2 for results with original values from a smaller set of sites). The model was run with (**A**), or without (**B**), the past land-use information (see Methods). PLU = past land-use (classed as “*agriculture*” or “*forest*”; see Supplementary Fig. S11).

After finding important effects of tree traits on SOC, we explored whether this relationship could be modulated by environmental characteristics of the sites (e.g., climate or soil properties). Our best model, which considered A_max_ and biomass, was not improved by adding site properties (Supplementary Table S2; Supplementary Fig. S9). Because the original values of A_max_ were not available for all sites, we used the PES index as the predictor for further linear models (Fig. 2B). The main finding was that the control of the PES on SOC was in interaction with past land-use, climate, and soil properties (Supplementary Table S2; Fig. 4 and Supplementary Fig. S10), which supported our initial expectations (hypothesis H2). More precisely, when tree species were on sites with a past history of agricultural land-use or fertilisation, the influence of plant traits was not significant anymore (Supplementary Fig. S11)^63^. Similar to past land-use, the effect of the PES on SOC was also influenced by climate. The PES effect was observed under most climates except under warm-wet conditions (Supplementary Fig. S12A). Finally, the influence of the PES on SOC tended to decrease with soil fertility: finely-textured and neutral soils exhibited only weak trends, while sandy acidic soils showed significant relationships between SOC pools and plant traits (Supplementary Fig. S12B, C). It is probable that both agronomic practices (such as liming and fertilisation) and the tendency of farmers to select the best lands (such as non-acidic, finely textured, soils)^64–66^ explain why past land-use and soil properties had a confounded effect on the PES-SOC relationship. Overall, the PES control over SOC weakened when site conditions became favourable for biological activity (wet-warm climates or fertile soils). This suggests that the improved SOC storage observed under some tree species may be due to biochemical recalcitrance of their necromass to biological decomposition^20^. When the environmental conditions are favourable for biological activity, they may enable the soil biocenosis to decompose any kind of necromass^41^, even if it is in theory less prone to degradation. Conversely, when the environmental conditions become harsher, biological activity is reduced and necromass decomposition becomes more dependent on the biochemical composition of the substrate, leading to higher SOC accumulation under tree species with more recalcitrant necromass, meaning low nutrient contents and high LDMC^12,67^, like found for conservative species.

The second factor controlling SOC storage was the standing biomass of the forest (Fig. 1): the higher the biomass, the greater the SOC (Supplementary Fig. S2G). This effect is linked to the observation that a larger biomass represents a larger production of necromass (i.e., litterfall (Supplementary Fig. S13), and dead fine roots), which in turn positively affects SOC accumulation^1,41^, supporting our hypothesis (H3a). High standing biomass also induces shading of the soil surface with negative consequences on forest floor decomposition^68^. In our study, tree species that produced high amounts of biomass were conservative species, like gymnosperms (Supplementary Fig. S14). This result is logical since, in natural environmental conditions generally characterised by limited supplies of resources, conservative species like gymnosperms can grow as fast as –or even faster than– acquisitive species like many angiosperms^69,70^.

**Fig. 5:**
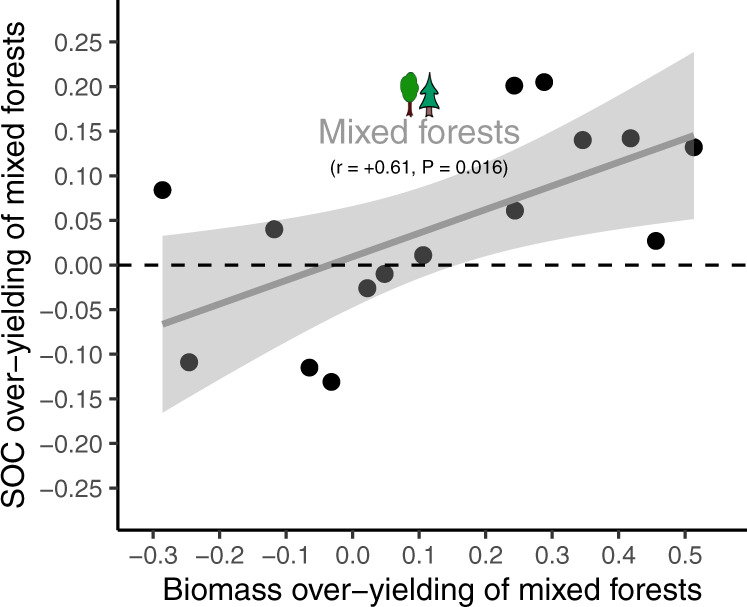
Increase in SOC sequestration by forest production overyielding induced by tree species mixtures. Values are indices of over-yielding due to tree species mixtures. Negative, zero, or positive values indicate that mixed forests performed worse, equally, or better on average than their respective mono-specific counterparts (i.e., the tree species that compose the mixture). All mixed forests are angiosperm-gymnosperm mixtures, excepted two cases (one angiosperm-angiosperm, one gymnosperm-gymnosperm, but of different tree species). The SOC pool considered is the whole soil profile (forest floor + topsoil). A linear regression was fitted (level of confidence of the error band = 0.95). Source data are provided as a Source Data files.

In addition to the direct effect of standing biomass on SOC, it is worth highlighting that mixing tree species in a given forest can indirectly increase SOC storage through positive effects on ecosystem productivity (hypothesis H3b). We found that, when mixtures produced more biomass than the relative mono-specific stands, it resulted in more SOC (Fig. 5). However, it is also important to note that mixed forests did not systematically store more SOC than the mono-specific forests (*P* = 0.161, *t* test = +1.46, *n* = 19), and on average they stored less SOC than the most efficient mono-specific forest at the same site (*P* = 0.011, *t* test = –2.83, *n* = 19).

Overall, results supported our initial hypotheses, and our study showed that tree species composition of a forest, and standing biomass, have a strong imprint on SOC storage, and should be considered in the current numerous initiatives for massive tree-plantings^10^. A simple recommendation could be to maintain high levels of standing biomass as it influences the storage of SOC positively. However, the carbon pool in trees is exposed to many hazards and it therefore appears reasonable to restrict the high-biomass strategy to regions that are not exposed to frequent disturbances^6^.

Our global study particularly showed that the tree species composition of a forest, by determining the dominant plant traits, was the first driver of its capacity to store SOC, but that the role of forest composition was context-dependent^71^, meaning that probably no unique global mitigation strategy exists^72^. Indeed, our study suggests that functional traits of tree species do not play an important role for SOC storage in warm-wet regions, like tropical regions. In those regions, the most urgent goal remains to protect or restore the natural composition of tropical forests^73,74^, whose surface area is shrinking. They have some of the highest levels of biodiversity worldwide, and are composed of broadleaved trees that cool the atmosphere due to their intensive evapotranspiration and moderate albedo^73,75^.

In non-tropical regions, the most efficient forest compositions to mitigate climate change depend on both climate and soil properties. Under warm-temperate climates or on finely-textured fertile soils, tree species with an acquisitive resource strategy, like many deciduous angiosperms symbiotically associated with arbuscular mycorrhizae, appear to be beneficial. In such environments they perform as well as conservative tree species at storing SOC while often growing fast^69^. In addition, our results suggested that SOC under acquisitive tree species may be more stable, rendering it sequestrated for longer. Finally, even if more studies are needed on this topic^76,77^, it seems that promoting deciduous angiosperms under warm-temperate climates may be more interesting than gymnosperms to cool the atmosphere through biophysical effects (evapotranspiration and albedo;^11,77^).

In the least favourable environments for biological activity, which are boreal climates or infertile soils, conservative tree species perform better at storing SOC than acquisitive tree species because of the strong control of the PES on necromass decomposition^60^. Under such conditions, ectomycorrhizal evergreen gymnosperms are more efficient to help mitigate climate change. Beyond their positive effect on SOC accumulation (Supplementary Fig. S8), conservative tree species are adapted to maintain their growth in constrained and competitive environments^69,70^. They are more able to profit from the CO_2_ enrichment of the atmosphere than acquisitive species^78^, and they only have a slight effect on air temperature through biophysical effects^72,77^. In boreal forests, where SOC constitutes the largest carbon pool of the ecosystem^2^ and where conifers are well-adapted to the widely-spread poor soils, forest composition is consequently a major force for enhancing SOC storage.

The way forests can best help mitigate climate change is context-dependent, implying that no single specific or functional tree composition constitutes a practical solution. Instead, maintaining high levels of standing biomass in areas not exposed to frequent disturbances, and restricting tree species to habitats where they are ecologically adapted –such as conservative species in harsh conditions– is one main guideline that emerged from this global study. It also follows that in tropical areas an efficient strategy to help mitigate climate change is to protect the natural forests, and at mid- and high-latitudes (or on fertile soils and on poor soils respectively) it would be to promote broadleaf tree species and needleleaf tree species, respectively. In addition, promoting mixed stands instead of mono-specific stands can improve the carbon sink in forests, provided that the composition of the mixture is well-designed^79^.

Because they play a major role in the Earth’s carbon cycle, forests can contribute substantially to climate change mitigation if properly protected and managed^80^. Within this perspective, in parallel to actions that reduce CO_2_ emissions, the present study showed that choosing the most efficient composition of tree species is a leverage close to hand.

## Methods

### Data collection: soil organic carbon

The process of data acquisition, selection and harmonisation is illustrated in the Supplementary Fig. S15 and in the Supplementary Tables S4–6. We conducted a systematic review for peer-reviewed journal articles, published before December 2018, from Web of Science, and Google Scholar with the search terms “(tree species OR forest) AND (soil organic carbon OR soil organic matter)”. We also used studies listed in two previously published meta-analyses^16,17^, or cited in already retained references (including references in English, French, Spanish, Portuguese, or Russian). For inclusion in the analysis we chose studies based on the following criteria: (1) the study reported soil organic carbon (SOC) or soil organic matter (SOM) concentrations or pools, at least in the topsoil layer and under at least two single-species forest stands; (2) the stands had to be older than 10 years^81^; (3) the stands had not experienced a major disturbance that differed between tree species, for at least 30 years (e.g., we rejected studies that compared natural forests with planted forests that were less than 30 years old); (4) The SOC concentration was <20% (i.e., we did not consider Histosols and peatlands). We considered studies at the scale of individual trees if the effect of other trees could be assumed to be negligible (e.g., tree clusters^82^, or lysimeters^83^). We excluded forests used for agricultural production, e.g., coffee. Together with the single-species requirement, this implied that we mainly considered common garden experiments and comparative plantations because spontaneous forests are typically composed of multi-species stands. We also rejected studies that examined highly disturbed reforested sites (mining spoil heaps, harbour sediments, city parks, etc.) as they often present artificial soil conditions. In a given site, we retained only the stands that had not been disturbed (e.g. wildfire^84^ or windthrow). For regions where data were scarce (e.g., Africa, Asia (except China), the Tropics, Russia etc.), the selection criteria could be applied with flexibility, e.g., comparisons of spontaneous stands with planted stands. When results from the same study sites were reported in different articles, only one article (the most complete or the most recent one) was included in our database. All studies and data were collegially evaluated to avoid inclusion/exclusion bias. A total of 114 articles from 110 independent author groups, covering 136 sites, and 454 stand-level observations matched the selection criteria and were included (by the two authors, working together) in this study (Supplementary Fig. S16, Source Data file, Supplementary Reference 1). In sites where, besides monospecific stands, two-species mixtures (50–50%) were tested, we also collected data from the mixed stands (29 stands). When data were presented graphically, the values were extracted using the free software WebPlotDigitizer version 4.3 (https://automeris.io/WebPlotDigitizer).

One major bias in soil carbon studies is caused by the comparisons of soil layers with a fixed thickness. Indeed, because the soil bulk density strongly depends on SOC, having two layers with different SOC contents generally implies different bulk density values and different soil mass, which means comparing two SOC pools but from non-equivalent soil layers. Taking bulk density into account improves comparisons done solely with SOC concentration values, but it still produces large errors^85,86^. To overcome this difficulty, it is recommended to use the *equivalent soil mass* approach^85^. We calculated SOC pools in equivalent soil masses (ESM) following a published procedure^87^. We converted SOC concentrations and bulk density values into SOC pools contained in soil layers with equivalent mass. In our study, we chose to describe soil profiles using ESM increments of 1000 Mg_-soil_ ha^−1^ (see Supplementary Fig. S17 for an example). In practice, ESM.0000-1000 is the SOC content of the uppermost soil layer with a mass of 1000 Mg (dry weight) of soil per hectare, whereas ESM.1000-2000 is the SOC content of the soil layer (also with a soil mass of 1000 Mg ha^−1^) just below the ESM.0000-1000 layer. The same rationale applies for the soil layers ESM.2000-3000 and so on. During data analysis, we sometimes grouped several ESM layers into one pooled layer. Notably, the pooled layer containing ESM.0000-1000, ESM.1000-2000, and ESM.2000-3000 was referenced to as ESM.0000-3000. To be used, the ESM method requires data for several soil layers. For studies that reported values for only a single topsoil layer depth (e.g., 0–15 cm), we estimated SOC in ESM units by taking into account the soil bulk density and layer thickness. When soil data contained only SOM values, we converted them to SOC values using the simple equation [SOC = SOM / 2.0], which is based on a review study^88^.

Information about the soil bulk density (BD) was given for only 36% of the stands so, for stands where BD information was missing, we estimated BD based on soil properties. For the forest floor layer (9% of data), we used 0.1346 as the bulk density, which is the mean value from different studies^89,90^. For the mineral soil layers, we first tested several pedo-transfer functions with a global dataset that contained measured BD values (Supplementary Table S7). It appeared that a function using SOC and soil texture as predictors^91^ was the most reliable in soil layers with low SOC concentration values (SOC ≤ 20 mg g^−1^), whereas a function based only on SOC^89^ was more reliable in richer soil layers. We applied these functions accordingly (43% of data). For the remaining data (12% of data) that contained no SOC concentration values but SOC pool values, we built a dedicated function based on data with known values of BD and SOC pool (*n* = 288; *P* < 0.001; r^2^ = 0.42). It is noticeable that our studied sites were homogeneous in terms of data availability: when values of soil bulk density were missing in a given site, it was for all the site’s stands. Because the main focus of our study was the comparison of different tree species in the same site, we assumed that possible errors induced by estimating the soil bulk density did not bias the relative ranking of the several tree species present in this site.

In forest soils, BD value is generally around 1 (i.e., 0.8–1.5 kg L^−1;^ ^91^), implying that an ESM layer of 1,000 Mg_-soil_ ha^–1^ corresponds approximately to a 10 cm thick soil layer. When a study reported SOC values for very thick soil layers, e.g., 30 cm, it was not possible to calculate SOC pools directly for ESM.0000-1000, and so on. To overcome this problem, we used the mean relative distribution of SOC within a given soil mass, which we calculated from the other studies. For example, for a topsoil layer of 3000 Mg_-soil_ ha^–1^ (ESM.0000-3000), the SOC contained in ESM.0000-1000, ESM.1000-2000 and ESM.2000-3000 was on average 45%, 30%, and 25%, respectively.

Finally, we obtained a dataset with values for nine soil layers: the forest floor and eight layers of the mineral soil horizons (from ESM.0000-1000, which is the uppermost mineral layer, down to ESM.7000-8000, which corresponds to a mean depth of 95 cm (range = 60–100 cm)). Whereas studying deep SOC is highly needed^92–94^, we did not do this because it was neither possible nor appropriate. It was not possible because published studies rarely report data for deep soil. In our compilation, data availability was 100% in ESM.0000-1000 but dropped quickly down to 12% in ESM.7000-8000. It was not suitable because of the age of the studied forest stands in comparison to the SOC age along the soil profile. In our study, the mean value of stand age was 44 years (22–54 years between the first and third quartiles). In soils, deep SOC is generally much older than the organic carbon contained in the uppermost layers^95,96^. A recent global analysis showed that around 80% of the SOC that has been incorporated over the last 40 years are in the 0–30 cm soil layer^97^. Taking into account our mean stand age (44 years), the tree species effect on total SOC quantity was not expected to be visible below a depth of 30 cm. For the analysis, we consequently retained data only from four layers (i.e., forest floor, ESM.0000-1000, ESM.1000-2000, and ESM.2000-3000), which we finally grouped into two layers (forest floor and mineral layers hereafter referred to as topsoil (ESM.0000-3000, which was the sum of ESM.0000-1000, ESM.1000-2000, and ESM.2000-3000)). In our study, the SOC content of the cumulated pools in topsoil represented a large proportion (66%) of the whole soil profile. Finally, retaining a limiting value of ∼30 cm depth also had the advantage of producing results that could be easily applicable at a large scale because most global soil inventories are carried out using a 0–30 cm basis^98,99^.

In addition to values of the total SOC content of soils, we collected information about SOC stability. For this, we collected data based on all approaches (i.e., incubation results, size and density fractions, chemical extractions) because there is no unique and standardised method to quantify SOC stability. The final dataset contained 25 studies that reported metrics for SOC stability at 30 sites.

### Data collection: auxiliary data

SOC content at medium or large scales can be controlled by soil properties, vegetation type (in this study: tree species), land-use, topography, soil parent material, and climate^100^. Consequently, we collected auxiliary data related to those factors. In a first step, we collected data about sites, stands, and soil characteristics by extracting information from each publication (or companion publications from the same author group). At the site scale, the collected information was: site name and location (longitude and latitude; to enable the identification of several studies about the same site), elevation, mean annual values of temperature and precipitation (MAT, MAP; °C, mm yr^−1^), past land-use, study design (e.g., number of blocks; see below the paragraph dedicated to data weighting), fertilisation history, soil name following the USDA classification (conversions from other classification systems were made following published references ^101,102^), soil weathering stage and soil parent material^102^, topsoil texture (based on the quantitative particle size analysis of the clay, silt and sand fractions, or estimated from the qualitative description and the USDA texture triangle: e.g., “silty loam” corresponds to clay = 150 mg g^−1^, silt = 650 mg g^−1^, and sand = 200 mg g^−1^), and other topsoil properties (e.g., pH, cation exchange capacity and its saturation value, total content in phosphorus). When the topsoil properties were presented at the stand scale, we calculated mean values at the site scale.

At the stand scale, we collected the following information: tree species name (updated to the current nomenclature where necessary), stand age (yr), stand density (stems ha^−1^), stand aboveground biomass (Mg ha^−1^), stand aboveground volume (m^3^ ha^−1^), stand basal area (m^2^ ha^−1^), mean value of stem diameter at breast height (cm), mean tree height (cm), flux of litterfall (Mg ha^−1^ yr^−1^), fine root biomass (Mg ha^−1^), and fine root production (Mg ha^−1^ yr^−1^). We also investigated the opportunity to collect more information (stand productivity, understorey composition and abundance, earthworms, etc.), but they were generally lacking in the original studies. To enable comparisons, when possible we calculated the different stand metrics (e.g., missing values of basal area were calculated from stand density and stem diameter). Then, when possible (25% of data), we estimated the missing values of stand biomass using linear regressions with other stand metrics (i.e., based on tree height [*n* = 37; *P* < 0.001; r^2^ = 0.51] or stem diameter [*n* = 50; *P* < 0.001; r^2^ = 0.45]), relying on the strong allometric relationships that exist among tree compartments^103–105^. We applied the same rationale to estimate the stand litterfall flux using the fine root production as predictor (3% of data; [*n* = 13; *P* = 0.017; r^2^ = 0.42]).

In a second step, we complemented our dataset (based on values provided in the original studies) with external sources using the latitude-longitude coordinates of the sites. These external sources were used to include variables that were not reported in the original studies (e.g., nitrogen atmospheric deposition), or to fill in gaps in our datasets when values were missing in the original studies. The extracted variables were: atmospheric nitrogen deposition^106^, Köppen-Geiger climate classes^107^, mean annual –or monthly– values of precipitation or temperature (MAP, MAT; http://worldclim.org), potential evapotranspiration and aridity index (https://cgiarcsi.community), elevation (https://www2.jpl.nasa.gov/srtm), soil properties^108,109^, and soil parent material^110^. The latter was simplified in a four class system (acid, intermediate, mafic, and calcareous)^102^. When possible, we checked the quality of the external sources by comparing them with the values that existed in the original studies (Supplementary Table S8). The consistency was fairly good (*n* = 30–99, slope = 0.75–1.12, r^2^ = 0.71-0.98) for soil texture and soil acidity (pH or base saturation), and excellent for climate data and site elevation (*n* = 79–110, slope = 0.98–1.03, r^2^ = 0.90–0.99). Conversely, we considered that the consistency for other variables (e.g., phosphorus content, cation exchange capacity, exchangeable cations, soil name, etc.) was not high enough and consequently we did not retain these external data. The relationships among the site properties are shown in the Supplementary Fig. S18.

### Data collection: plant functional traits

We collected values of the functional traits that define the Plant Economics Spectrum^49^, and widely expanded this list by adding plant traits, leaf traits, and root traits (n = 74). The values of plant functional traits were determined based on several approaches. First, we used the TRY trait database^111^. We downloaded the publicly available data related to the 178 tree species included in our SOC database (www.try-db.org; access date: January 2019). This dataset contained 938,978 individual trait values for the 74 studied traits.

In a second stage, we complemented our trait database with data from other published sources like FRED^112^, China Plant Trait database^113^, ORNL DAAC leaf traits (https://daac.ornl.gov), and TTT^114^. We also used trait values from the references used for building our SOC database as well as 143 other published studies (Supplementary Reference 2). The latter references were included because, when evaluating the data quality of the other sources (including TRY), we realised that some important functional traits had no value for many of the tree species present in our SOC database. To look for additional studies to fill in data gaps, we searched in Google Scholar and Web of Science using specific keywords, for instance: {“cupressus lusitanica” AND (SLA or “specific leaf area”)} or {“cupressus lusitanica” AND (leaf OR foliage OR foliar OR needle) AND nitrogen}.

For traits such as leaf life span (classed as deciduous or evergreen) or tree maximum height (in metres), we also consulted the following websites: Wikipedia (in English, French, Spanish, and Portuguese; https://www.wikipedia.org), https://www.conifers.org, http://efloras.org, http://www.fs.fed.us, https://www.gbif.org, http://issg.org/database/welcome/, www.iplantz.com, https://pfaf.org, http://www.tree-guide.com, http://tropical.theferns.info, https://wiki.bugwood.org/Main_Page, http://www.worldagroforestry.org. Missing values for seed mass and wood density were found respectively in the Kew Seed Information Database (http://data.kew.org/sid) and the Tree Functional Attributes and Ecological Database (http://db.worldagroforestry.org/wd). The mycorrhizal status of tree species that were not in the TRY database was complemented using dedicated references ^115–117^. When, despite these references, a status was still missing, a value based on the genus or the family was assigned.

In total, the second trait dataset was composed of ∼48,700 values, from which we retained 6,611 values (about 71 traits) that were related to the tree species present in our SOC database. This dataset was merged with the TRY dataset.

Data quality was carefully assessed for all traits by checking if *(i)* each category was homogeneously coded (e.g.,: ectomycorrhizal tree species were coded either “E.” or “EM”; deciduous species were coded in 12 different manners such as “deciduous”, “deciduous type 1”, “winter deciduous”, etc.), *(ii)* the number of values for a given variable –or category– was high enough to be used (e.g., leaf palatability was discarded as n = 7), *(iii)* the unit used for a numerical trait was the same for all values (e.g., leaf carbon content was found in g_-C_ g_-DW_^−1^, mg_-C_ g_-DW_^−1^, %, or mmol_-C_ g_-DW_^−1^). When necessary, the values of the categorical traits were homogenized (renaming, or merging similar classes). Similarly, the units of the numerical traits were homogenised, and the values were changed accordingly. After this quality assessment stage, we excluded traits for which most of the values were missing (e.g., plant light requirement, or root type). We did the same for traits that presented many classes, which resulted in having only a few tree species per class (e.g., Grime’s groups or reproductive phenology), and for traits for which the classes could not be homogenised because of classification inconsistencies among the original data sources (e.g., soil texture requirement of the tree species).

Duplicated values from the same original study, but present in several sources, were removed. When three traits were interconnected (e.g., leaf C content, leaf N content, and leaf C:N ratio) and one value was missing, the latter was calculated based on the two non-missing values. When several values existed for the same trait-species combination, mean values were calculated. Using mean values for functional traits may introduce some additional variance in results because trait values in a given plant species are plastic and acclimate to environmental conditions from site to site^118,119^. However, interspecific trait variation generally exceeds intraspecific trait variation^120,121^. For instance, the interspecific coefficients of variation for SLA, LDMC, leaf N content, and leaf P content (*n* = 65–144 species) were 73%, 19%, 44%, and 45% in our database, while the intraspecific coefficients of variation of these variables were reported to be ∼19%, ∼10%, ∼18%, and ∼22% for two tree species (*Pinus koraiensis* and *Fraxinus mandshurica*;^122^). Similarly, the interspecific coefficient of variation of the leaf photosynthetic maximum rate was very high in our database (CV = 93%, *n* = 95), which is in line with the global variation of this trait^123^. Consequently, because our study encompassed a wide range of functional plant types studied at the global scale, we assumed that our approach based on mean trait values was reliable due to high interspecific trait variation^124^.

We investigated the relationships among functional traits using Principal Component Analyses. When several traits were well-correlated to each other (example 1: leaf dry matter content, leaf strength, leaf thickness, and leaf lifespan; example 2: leaf photosynthetic maximum rate, leaf photosynthesis carboxylation capacity, leaf stomatal conductance, and specific leaf area; example 3: leaf content in C, N, P, K, Ca, Mg and C:N and N:P ratios), we removed the traits with the highest frequency of missing values (in the examples above we removed strength, thickness, lifespan, photosynthesis carboxylation capacity, stomatal conductance, K content, Mg content).

To decrease the number of retained traits even further, and increase the rate of non-missing values, we used a gap filling procedure for five foliage traits (A_max_, C, N, P, Ca): first, we built relationships among redundant traits (*cf*. example 2, above): when, for a given tree species, the retained trait had a missing value, it was estimated based on the removed traits (e.g., leaf photosynthetic maximum rate estimated based on the values of the leaf photosynthesis carboxylation capacity, or leaf stomatal conductance). Furthermore, we observed that, for a given element (C, N, P, Ca), the contents in leaves, litter, and fine roots were all inter-correlated^56,125,126^ while root traits are poorly known^127^. Consequently, we built regressions between various related plant traits, and estimated the missing values for leaf nutrient content based on litterfall nutrient content, or fine root nutrient content (r^2^ = 0.44–0.99; estimated data = 1.5–13.7% of data; Supplementary Table S9).

For the leaf C content, the remaining missing values were estimated using the plant genus, family, or spermaphyte group, based on published reviews^56,128,129^. Out of the 454 stands, 4 stands from scarcely represented areas only reported the tree genus but not the species name (i.e., *Acer* spp., *Eucalyptus* spp., *Pinus* spp., and *Quercus* spp.). For these few cases, we used the mean trait values of all the species in that particular genus.

The dataset containing the final trait values could not be provided in this publication because we agreed to the intellectual property guidelines for the TRY initiative, which prohibit the redistribution of the trait data received (see a summary in the Supplementary Table S10). However, all the trait data that we used are publicly available, and thus it is possible to rebuild our dataset following the methods presented above. We also made the trait data available for the reviewers.

Compiling values of plant traits for a global scale study is exposed to two pitfalls: *(i)* it is difficult to have non-missing values for uncommon plant species, and *(ii)* the high number of traits (which are dependent on each other) makes the data interpretation difficult. To circumvent these problems, in addition to the approach described above, we used an approach based on Principal Component Analysis (PCA), which is particularly adequate to synthetically describe datasets with a high level of covariance among variables^130^. First, we produced estimated values for missing values (using the functions *estim_ncpPCA* and *imputePCA* from the *missMDA* package^131^). Then, we performed a PCA analysis (function *PCA*) to produce an integrative index of our explanatory variables. In practice, this integrative index was the score on the first axis of the PCA. We produced two integrative indexes: (i) an index score of the PES (produced by a PCA that took into account the main traits: leaf size, A_max_, LDMC, SLA, SRL, and leaf contents in lignin, C, P, N, P, Ca, and C:N ratio; Supplementary Fig. S19), and (ii) a Biomass index (based on stand biomass, annual litterfall, tree species maximal height, species growth rate, seed mass, and wood density; Supplementary Fig. S20). Adding categorical variables (such as spermaphytes) either did not change the results or made them worse, and were consequently not used for the PCA.

We used both original trait values and the index values in our study. Original values were mainly used to test the main effects of the studied drivers (i.e., plant traits and stand biomass) because they were not estimated values. Nevertheless, original values were more or less missing, rending difficult any multi-variables analysis. On the other hand, because index values were calculated for most sites, they were used to confirm results on a larger scale, and to test the influence of the environmental conditions (e.g., temperature or soil clay content) on the PES-SOC relationship (see below). However, as these indexes were only proxies, their use substantially decreased the explanatory power of the analyses.

### Dataset compilation: phylogenetic distance

We built a phylogenetic tree from all species in our SOC dataset to determine whether life history characteristics, represented by phylogenetic proximity, constitute a characteristic that affects SOC. The phylogenetic distance between two species was estimated, based on relevant literature and using the approach of the *most recent common ancestor*. To do this, we built a phylogenetic tree that contained all the families included in our dataset about soil organic carbon (Supplementary Fig. S21). The distance between angiosperms and gymnosperms was fixed as 350 Myr^132^. Following the same reference ^132^, the distance between *Cupressales* and *Pinales* was set at 273 Myr. Within the gymnosperms, the distances among clades down to genera were estimated based on a recent study^133^. The distances between two species of the same genus (*Abies*, *Picea*, or *Pinus*) were fixed based on dedicated studies^134–136^. Within the angiosperms, we first determined the phylogenetic distances among families^137^. Then, we used the Angiosperm Phylogeny Website (http://www.mobot.org/MOBOT/research/APweb; accessed in January 2020) and relevant references ^132,138–142^ to estimate the distances between two species that were in the same family or genus.

In total, our dataset about soil organic carbon contained 787 combinations of tree species pairs (by considering pairs only within the same site). For instance, in a common garden with stands of four different tree species, there are six possible pairs of tree species comparisons. Overall our dataset contained 641 distinct pairs because some pairs were found in several sites. The phylogenetic tree contained 629 non-missing values of phylogenetic distance (i.e. 98.1% of all pairs). For the remaining pairs (*n* = 12; 1.9%), we estimated the missing values as being half of the crown age of the clade containing the two tree species.

The influence of the phylogenetic distance was studied by investigating whether the absolute difference of soil organic carbon between two tree species within the same site was correlated to their phylogenetic distance.

### Data handling and normalisation

We applied weights to each case study in order to take into account the robustness of the study design. The three criteria considered were: the site design (C1), the number of blocks (C2), the number of sampled soil profiles (C3). For each criterion, a weight value was given or calculated (normalised to [0-1]; see below).

For the site design (C1), the weight value (W_S_) was fixed as 1.00, 0.66, 0.33, and 0.00 respectively for: (i) common gardens (i.e., stands installed specifically for use as comparisons, following a unique installation protocol), (ii) comparative plantations (i.e., stands of similar age and planted following similar methods, but without the intention to compare), (iii) spontaneous forests (i.e., non-planted stands, fairly mono-specific (>80% of biomass or stem density), and in similar site conditions), and (iv) heterogeneous designs (i.e., comparisons of spontaneous stands with plantations; the case studies based on this design were retained only in regions where reliable data were lacking).

For the number of blocks (C2), the weight value (W_B_) was calculated as: W_B_ = [log_10_ (nb.blocks)]

For the number of soil profiles (C3), the weight value (W_P_) was calculated as: W_P_ = [log_10_ (nb.profiles)] / 2

All weight criteria ranged between 0 and 1. The data weight (W_data_) was then calculated as the mean of the weight criteria (W_S_, W_B_, W_P_). To avoid having a null statistical weight, W_data_ values lower than 0.05 were corrected to this threshold value. Overall, W_data_ was on average 0.36 (range = 0.05-0.69). Values of W_data_ were declared as a statistical weight factor during the data analyses (when the SAS procedures or R packages enabled applying such a correction).

At the global scale, the SOC content varies a lot among sites^5^, which implies that inter-site variability is generally much larger than intra-site variability induced by plant species^18^. To examine the effect of plant traits on SOC pools without having the influence of the site characteristics, we calculated relative responses of SOC. The relative response approach enables to normalise values relatively for each site, removing the direct influence of the site conditions^143^. Similarly, because the list of present tree species varied from site to site, it was not possible to use the absolute values of functional traits as predictors and we consequently used relative values also for traits.

For variables with a continuous distribution (e.g., functional traits such as leaf N content, SOC pool size, or stand biomass), we calculated a relative value for each stand by normalising values relatively to the site^144^. For instance, in a study reporting results from three stands (A, B, C), we calculated the response ratio of each stand following this formula (here for the stand A):

1 $${RR}.A={{\log }}\left(\frac{{SOC}.A}{{SOC}.{site}}\right)$$

where *RR.A* is the relative response ratio for stand A of the study, *SOC.A and SOC.site* are soil organic carbon (equivalent soil mass) values for stand A and the whole site reported in the study (average of all stands at a site). If RR.A < 0, then the species of stand A had a negative effect on SOC accumulation at this site. If RR.A > 0, then the species had on average a positive effect on SOC. If RR.A ≈ 0, then there was no effect of the tree species on SOC. We followed the same formula for all plant traits of interest (see Supplementary Table S11 for an example).

We should note that the dataset in the Source Data file does not contain all possible relative values. Indeed, the values presented are only those that could be calculated when all the tree species of a given site had non-missing values. During data analysis however, if we studied a possible relationship between two variables (e.g., between SLA and SOC), we recalculated all the possible relative values provided that at least two species within the same site had non-missing values.

When the tree species in a given site were studied using a categorical approach with two classes (e.g., angiosperms versus gymnosperms, or N-fixing species versus non-fixing species), we calculated the mean SOC value per class (e.g., for angiosperms). In practice, firstly we recalculated the mean normalised values per class in order to avoid pseudo-replicated values in sites with more than two tree species of the same class. Then, we calculated a log relative response, commonly used in meta-analyses^145^. For instance, with angiosperms and gymnosperms (see also Supplementary Table S11), the relative values of SOC were:

2 $${RR}={{\log }}\left(\frac{{SOC}.{angiosperms}}{{SOC}.{gymnosperms}}\right)$$

Because the final dataset about mixed forests did not contain all the data necessary to calculate ESM values, we chose to use SOC pool values in this case. We compared the SOC pools (forest floor + mineral soil) under mono-specific and mixed stands by calculating an *overyielding* index, which was a relative response ratio of stand characteristics and SOC pools following the formula:

3 $${RR}.{overyielding}={{\log }}\left(\frac{{AB}}{{mean}(A,B)}\right)$$

With A, B and AB as the absolute values for the two monospecific stands (species A or species B) and the mixed stand (50%-50% mixture of the species A and B). Negative, null, and positive values indicated that the mixed stand had a lower, equal, or higher value than the mean value of the monospecific stands (overyielding corresponds to cases where RR.overyielding > 0).

We also calculated a *transgressive overyielding* index:

4 $${RR}.{transgr}.{overyielding}={{\log }}\left(\frac{{AB}}{{max }(A,B)}\right)$$

Positive values indicated that the mixed stand had a higher value than the highest value of the monospecific stands.

### Data analysis

We did not remove any outliers, and used all the data we had for our analyses (i.e., regressions, correlations, and tests). However, to produce easily readable graphs, we sometimes reshaped the axes scale which made a few (*n* = 1–3) outliers invisible, but they were still taken into account for the statistics. In graphs showing regressions, error areas are confidence of intervals. Boxplots represent the median, the first and third quartiles, and 1.5 × the inter-quartile range.

During data analysis, we used firstly the *Random Forest* approach to identify the main drivers of SOC (*randomForest* R package^146^). We applied this method to absolute values of SOC to determine the drivers at the global scale (i.e., inter-site variability) and to relative values of SOC to determine the drivers at the site scale (i.e., intra-site variability). The identified drivers of SOC at the site scale (i.e., plant traits and stand properties) were used in subsequent analyses.

Then, we examined the effect of tree species on SOC by evaluating relationships between the RR values of plant traits and SOC using simple linear regressions and Spearman’s rank correlation coefficients. In a second step, we used a modelling approach based on multiple linear regressions. In practice, we modelled the RR SOC values (i.e., combined pools of forest floor plus ESM.0000-3000) with the most predictive variable (i.e., plant traits and stand properties, in normalised values), and then analysed the possible existence of relationships between the residuals and other variables. The built model was evaluated in two ways: with the *performance* package in R (see below), and using an independent dataset. This later dataset was composed of the sites that had non-missing SOC values for the forest floor, and for the ESM.0000-1000 soil layer (or the ESM.0000-2000 layer), but had no value for the deeper layers (n = 43 stands from 23 distinct sites distributed over 4 continents, under cold or temperate climates, and on 6 different soil types). The calibration dataset and the validation dataset were initially not strictly comparable because the contribution of the forest floor layer to the combined SOC pool (i.e., forest floor + topsoil) was lower for the calibration dataset (i.e., topsoil = ESM.0000-3000; forest floor = 19% of the combined pool on average) than for the validation dataset (23% and 33% for the cases where topsoil = ESM.0000-2000 and ESM.0000-1000), and because the influence of plant traits on carbon pools were of unequal magnitude in the forest floor and the topsoil. Indeed, the normalised values of SOC pools (RR.SOC) for the validation dataset were 11.5% (forest floor + ESM.0000-1000) and 6.4% (forest floor + ESM.0000-2000) higher than for the calibration dataset (forest floor + ESM.0000-3000). We consequently modified the validation dataset by applying a correction factor of 1.115 or 1.064, depending on the case.

The influence of tree species on SOC was also investigated using a categorical approach (e.g., gymnosperms versus angiosperms, evergreen versus deciduous, ectomycorrhizal versus arbuscular mycorrhizal stands, N-fixing species versus non-fixing stands). After data normalisation (see Eq. 2), the recalculated values were used to test the existence of a significant difference between the two classes (Bonferroni test, Wilcoxon test, or Mann-Whitney test; two-sided tests).

Eventually, we tested whether a SOC-trait relationship (which was significant at the global scale) was influenced by site conditions (e.g., atmospheric N deposition, climate, soil properties, past land-use). In practice, SOC was modelled based on trait values, and possible interactions between trait values and site conditions were also tested:

$${{{{\rm{SOC}}}}} \sim ({{{{\rm{Trait}}}}}\times {{{{\rm{Biomass}}}}})+\{({{{{\rm{Trait}}}}}+{{{{\rm{Biomass}}}}}):({{{{\rm{Site.conditions}}}}})\}$$

with N deposition, climate, soil pH, soil texture, and past land-use (PLU) as site conditions.

The site conditions were not tested as direct effects because of the way SOC values were normalised. Indeed, the normalisation (see Eq. 1) implied that, for a given site, the mean SOC normalised value was by definition always close to zero. Because all the stands within a given site share the same values of site conditions, the latter variables had no influence on SOC normalised values.

To avoid collinearity among the site descriptors, we simplified the model as follows:

- Climate descriptors (MAT, MAP, aridity, PET, *f*_climate_, water balance) and site location (latitude, elevation) were plotted in a PCA. It resulted that the axis 1 (52.4% of the variance) was best explained by *f*_climate_, MAT and MAP. The *f*_climate_ metric is designed to increase with concomitant increasing MAT values and increasing MAP values, and indicates if the climatic conditions are favourable for biological functioning (enough water and not too cold)^102^. Because *f*_climate_ explained well the climatic conditions (see above), and because it was well correlated with other climatic descriptors (Supplementary Fig. S22), it was retained as the unique climatic descriptor in the models.

- Atmospheric N deposition was highly correlated to climatic conditions (Supplementary Fig. S18) and was consequently removed from the models.

- Variables that described soil texture (i.e., clay, silt, and sand contents) were logically correlated to each other (Supplementary Fig. S18). Because sand content was well-correlated to both clay content and silt content, we retained sand content as unique descriptor of soil texture.

These simplifications resulted in the following model:

$${{{{{\rm{SOC}}}}}} \sim ({\rm Trait}\;\times {\rm Biomass})+\{({\rm Trait}+{\rm Biomass}):({f}_{{{{{{\rm{climate}}}}}}}+{{{{\rm{Sand}}}}}+{{{{\rm{pH}}}}}+{{{{\rm{PLU}}}}})\}$$

However, in our dataset the categories of past land-use (PLU) were not randomly distributed as the “agriculture” PLU category had significantly more favourable conditions (i.e., higher values of *f*_climate_ and soil pH; lower values of soil sand content) than the “forest” PLU category (*P* = 0.003 to 0.045). To take into account this dependency among site descriptors, the data was also analysed with a model without PLU:

$${\rm SOC} \sim ({\rm Trait}\;\times {\rm Biomass})+\{({\rm Trait}+{\rm Biomass}):({f}_{{{{{{\rm{climate}}}}}}}+{\rm Sand}+{\rm pH})\}$$

It should be noted that, while our dataset with SOC content data was large enough to be analysed with these models, the other datasets (about SOC stability or the effects of mixed forests) were too small to enable such an analysis. The later datasets were consequently analysed with simpler models, based on data availability.

We tested these models with a R function that uses the AIC as criterion (*ols_step_forward_aic* from the *olsrr* package^147^). We also used mixed models (*lmer* from *lme4*; assigning the site identity as a random effect) and multi-regression (*glmulti* and *stepAIC*). Because all these methods gave very similar results, we presented only those based on the *olsrr* package. In the case of SOC stability, we used a mixed effects model to take into account that this variable was measured with different lab methods (Supplementary Fig. S23). The quality of the models was evaluated with the *performance* package.

Data warehouse, data handling and preliminary analyses were done using SAS (9.4). Final data analyses and graphs were made with R (4.0.3)^148^, including the packages *car*, *ggeffects*, *ggplot2*, *ggpubr*, *glmulti*, *lme4*, *missMDA*, *multcomp*, *nparcomp*, *olsrr*, *performance*, *psych*, *magick*, *randomForest, RcmdrMisc*, and *VIM*^131,146,148–161^. The pictures used in graphs were creative commons licensed (CC0), and published in dedicated websites (e.g., https://publicdomainvectors.org).

### Reporting summary

Further information on research design is available in the Nature Research Reporting Summary linked to this article.

## Supplementary information

Supplementary Information file

Reporting Summary

## Data Availability

The SOC data generated in this study have been deposited in the https://data.inrae.fr database under accession link 10.15454/LJRFJR. The raw data from the TRY database are protected and are not available due to data privacy laws. The references used to compile SOC data and plant traits are provided in the Supplementary Information.

## Code availability

Code of this study is available in the https://data.inrae.fr database under accession link 10.15454/LJRFJR.
