# Supplementary material for: Tree functional traits, forest biomass, and tree species diversity interact with site properties to drive forest soil carbon
Source: Nat Commun. 2022 Mar 1;13:1097. doi: 10.1038/s41467-022-28748-0 (PMC8888738; doi:10.1038/s41467-022-28748-0)
Supplement: Supplementary file 1 — Supplementary Information file [file 41467_2022_28748_MOESM1_ESM.pdf]

Tree functional traits, forest biomass, and tree species diversity interact with site properties to drive forest soil carbon

L. Augusto & A. Boča

Supplementary Information

Supplementary Figures (S1 to S23)

Supplementary Fig. S1 | Main variables explaining the SOC pools at the global scale.
Supplementary Fig. S2 | Global influence of plant traits and forest biomass on SOC pools.
Supplementary Fig. S3 | Relationships among the traits that constitute the Plant Economics Spectrum.
Supplementary Fig. S4 | SOC pools and phylogenetic distance among tree species.
Supplementary Fig. S5 | Model explaining the SOC pools as a function of the photosynthetic capacity of tree species and stand biomass.
Supplementary Fig. S6 | Evaluation of the model using the calibration dataset.
Supplementary Fig. S7 | Global influence of the photosynthetic capacity of tree species on SOC pools.
Supplementary Fig. S8 | Soil organic carbon under gymnosperms and angiosperms.
Supplementary Fig. S9 | Evaluation of the model based on imputed values, including past land-use (Figure 4A).
Supplementary Fig. S10 | Evaluation of the model based on imputed values, without past land-use (Figure 4B).
Supplementary Fig. S11 | Modulation of the imprint of tree species on forest SOC by past land-use and site fertilisation.
Supplementary Fig. S12 | Modulation of the imprint of tree species on SOC by climate and soil properties.
Supplementary Fig. S13 | Influence of stand aboveground biomass on stand litterfall flux.
Supplementary Fig. S14 | Aboveground stand biomass in gymnosperm and angiosperm forests.
Supplementary Fig. S15 | Data flow of the study.
Supplementary Fig. S16 | Map of study site locations.
Supplementary Fig. S17 | Equivalent Soil Mass calculation.
Supplementary Fig. S18 | Relationships among the site properties.
Supplementary Fig. S19 | Principal Component Analysis used to produce the index score of the Plant Economics Spectrum (PES).
Supplementary Fig. S20 | Principal Component Analysis used to produce the index score of the forest standing biomass.
Supplementary Fig. S21 | Phylogenetic tree of the species of the study.
Supplementary Fig. S22 | Relationships among climatic descriptors.
Supplementary Fig. S23 | Differences of SOC decomposability between arbuscular mycorrhizal tree species and ectomycorrhizal tree species, as influenced by the method used to quantify the SOC decomposability.

Supplementary Tables (S1 to S9)

Supplementary Table S1 | Influence of plant functional traits and stand properties on SOC pools.
Supplementary Table S2 | Models predicting the SOC content.
Supplementary Table S3 | Mean values of functional traits of several tree plant functional types.
Supplementary Table S4 | List of the variables used in the study.
Supplementary Table S5 | PRISMA abstract checklist.
Supplementary Table S6 | PRISMA checklist.
Supplementary Table S7 | Pedotransfer functions tested to estimate missing values of soil bulk density.
Supplementary Table S8 | Comparisons between observed values and global values describing the site properties.
Supplementary Table S9 | Regressions used for the gap filling procedure of the plant functional traits.
Supplementary Table S10 | Data availability for the main functional traits used in the study.
Supplementary Table S11 | Fictive example of the values available in supplementary information and the values used during data analysis to test a possible relationship between two variables.

Supplementary References

Supplementary Reference 1 – List of references used to build the Soil Organic Carbon dataset
Supplementary Reference 2 – List of references used to complement the Plant Functional Traits dataset
Supplementary Reference 3 – List of references used to build the mixed forests dataset and the SOC stability dataset

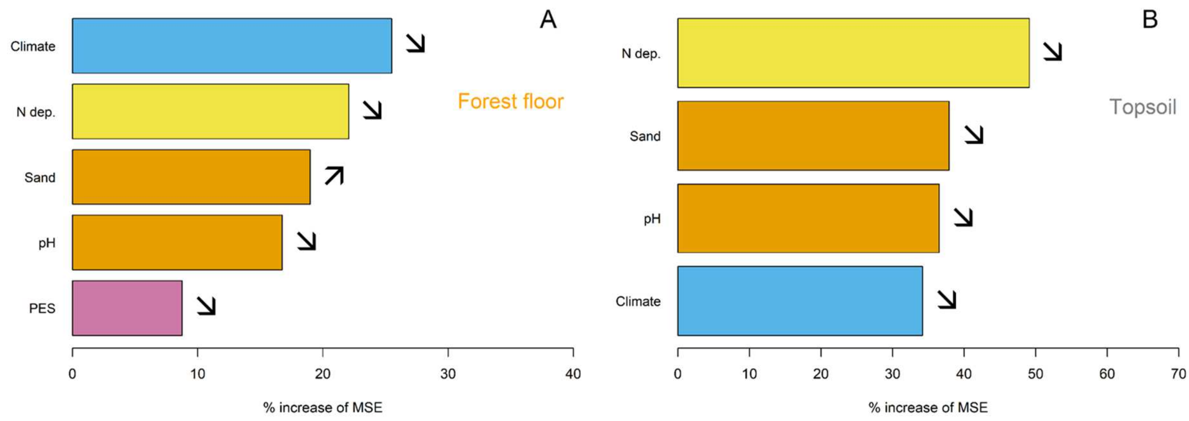

Supplementary Fig. S1 | Main variables explaining the SOC pools at the global scale. The SOC pool was modelled using their absolute values, enabling the evaluation of the influence of the site properties on SOC content. The studied pools were: the forest floor layer (A), and the topsoil layer (B). The predictors were a climatic descriptor (f_{climate} ranging from 0 [unfavourable to biological activity] to 1 [favourable], in blue; see Methods), soil properties, in brown (sand content and pH value), nitrogen atmospheric deposition, in yellow, and the index score of the Plant Economics Spectrum (PES; see Methods) of the tree species, in violet. The influence of the variables was assessed using the percentage of increase of mean square error (MSE) after running the Random Forest approach (see Methods). Arrows indicate positive (↗) or negative (↘) effects of the predictors on SOC.

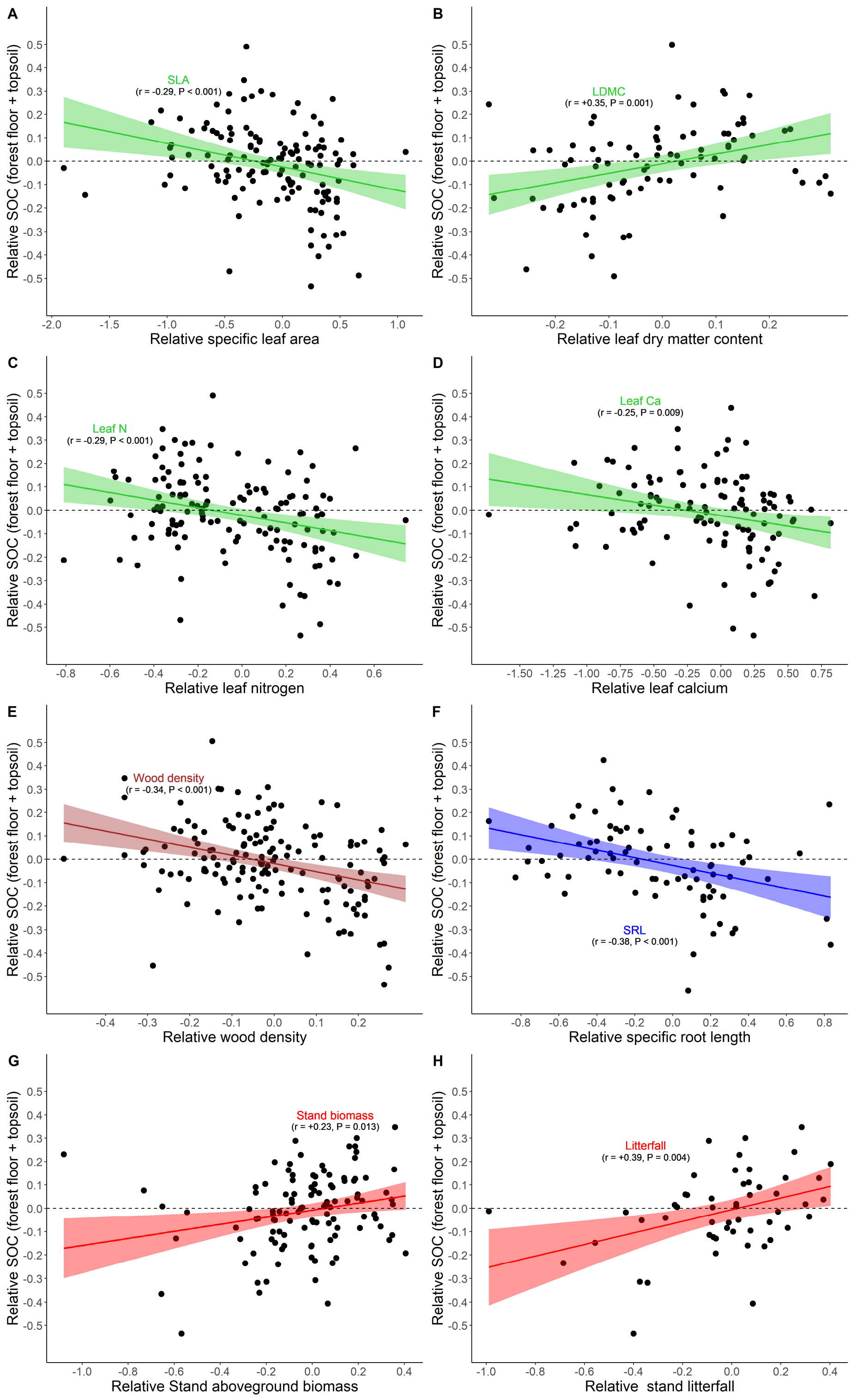

Supplementary Fig. S2 | Global influence of plant traits and forest biomass on SOC pools. Values are normalised (see Methods). Values of r^2 are 0.05-0.15 (see panels for r values and P values). Results for the leaf photosynthetic capacity (A_{max}), leaf size, leaf C:N, and seed mass are not shown here (see Figure 2 for A_{max}). For these latter traits, the relationships were significant ($|r| = 0.21$-0.46; $r^2 = 0.04$-0.21; $P < 0.050$). Linear regressions were fitted (level of confidence of the error band = 0.95).

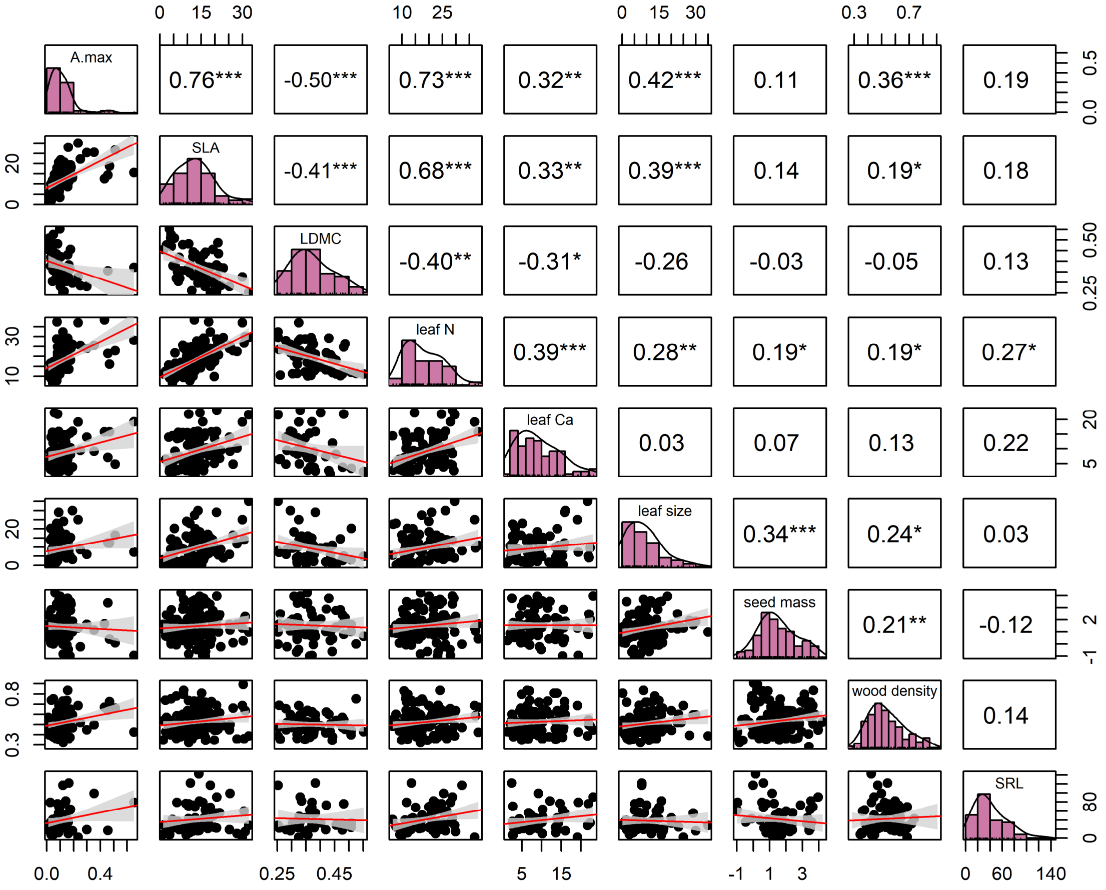

Supplementary Fig. S3 | Relationships among the traits that constitute the Plant Economics Spectrum. To avoid pseudo-replications, we used only a subset of our data, containing one set of trait values per tree species (n = 59-178, depending on the trait). The matrix shows the results of Spearman's rank correlation coefficients. The symbols *, **, and *** indicate correlations with P values respectively as follows: P < 0.05, P < 0.01, and P < 0.001.

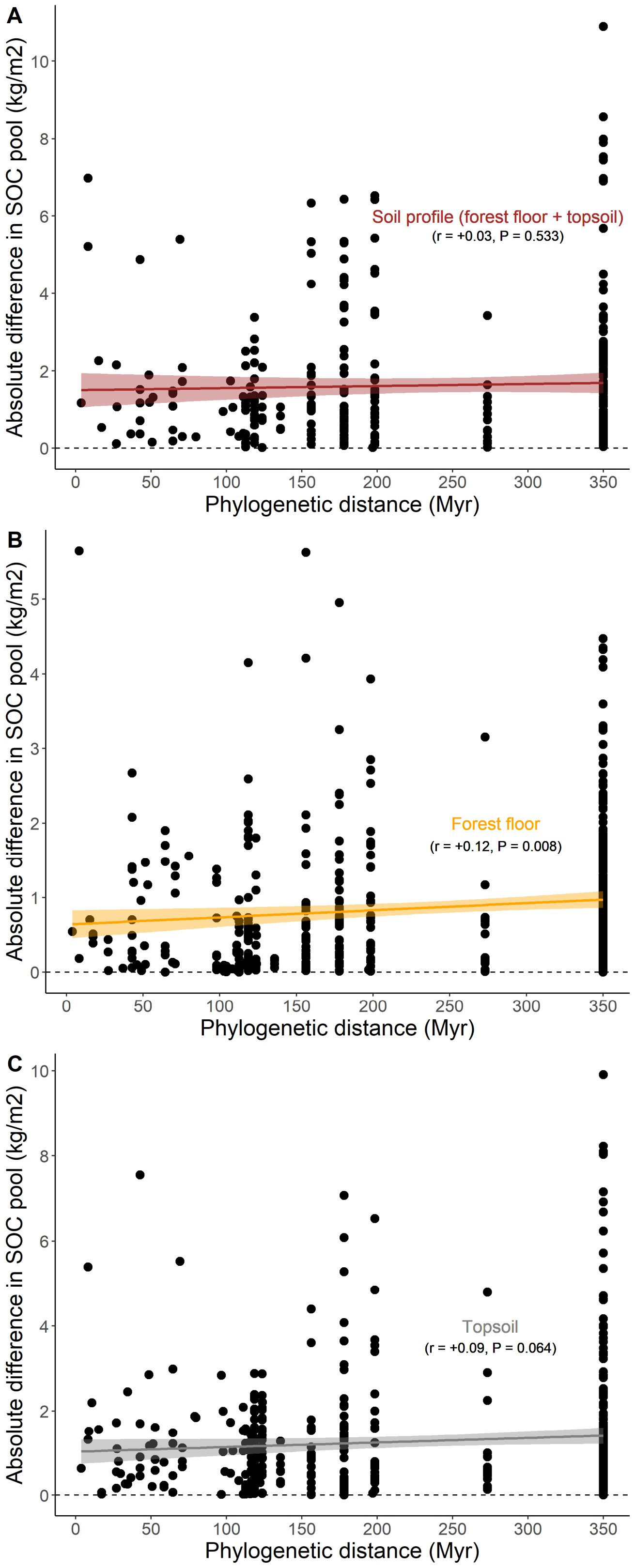

Supplementary Fig. S4 | SOC pools and phylogenetic distance among tree species. SOC pools are: whole soil profile (A), forest floor (B), and topsoil (C). Values are normalised (see Methods). Each dot is a pair of mono-specific stands of different tree species growing in the same site. The phylogenetic distance between the two species of a given pair is in millions of years. Linear regressions were fitted (level of confidence of the error band = 0.95).

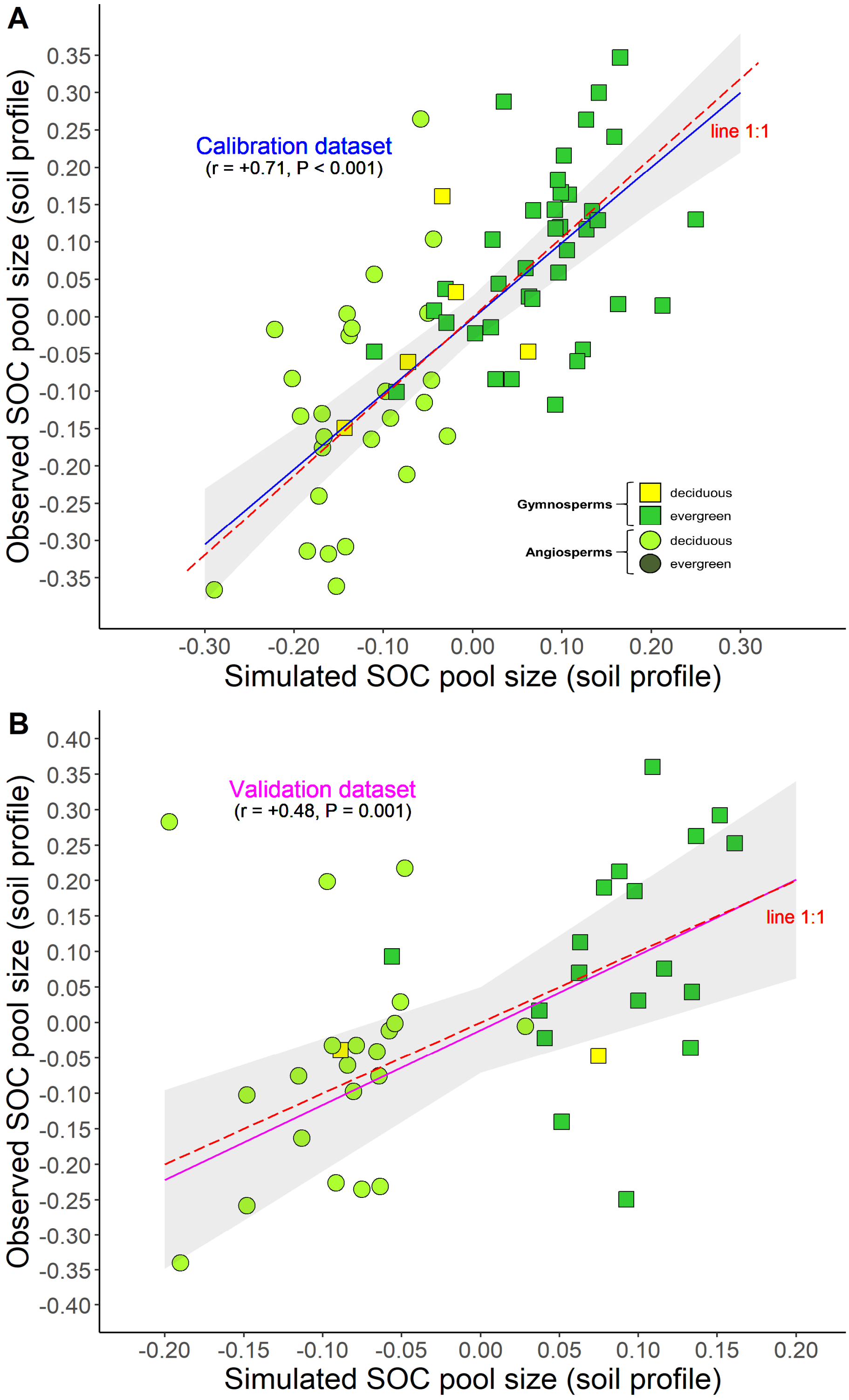

Supplementary Fig. S5 | Model explaining the SOC pools as a function of the photosynthetic capacity of tree species and stand biomass. SOC pools are for the soil profile (forest floor + topsoil). Panels show the performance of the model (based on leaf photosynthetic capacity of tree species and standing biomass of stands) with: the calibration dataset (A) and an independent dataset used for validation (B). Values are normalised (see Methods). Linear regressions were fitted (level of confidence of the error band = 0.95). Linear regressions take into account data reliability as weighting factor.

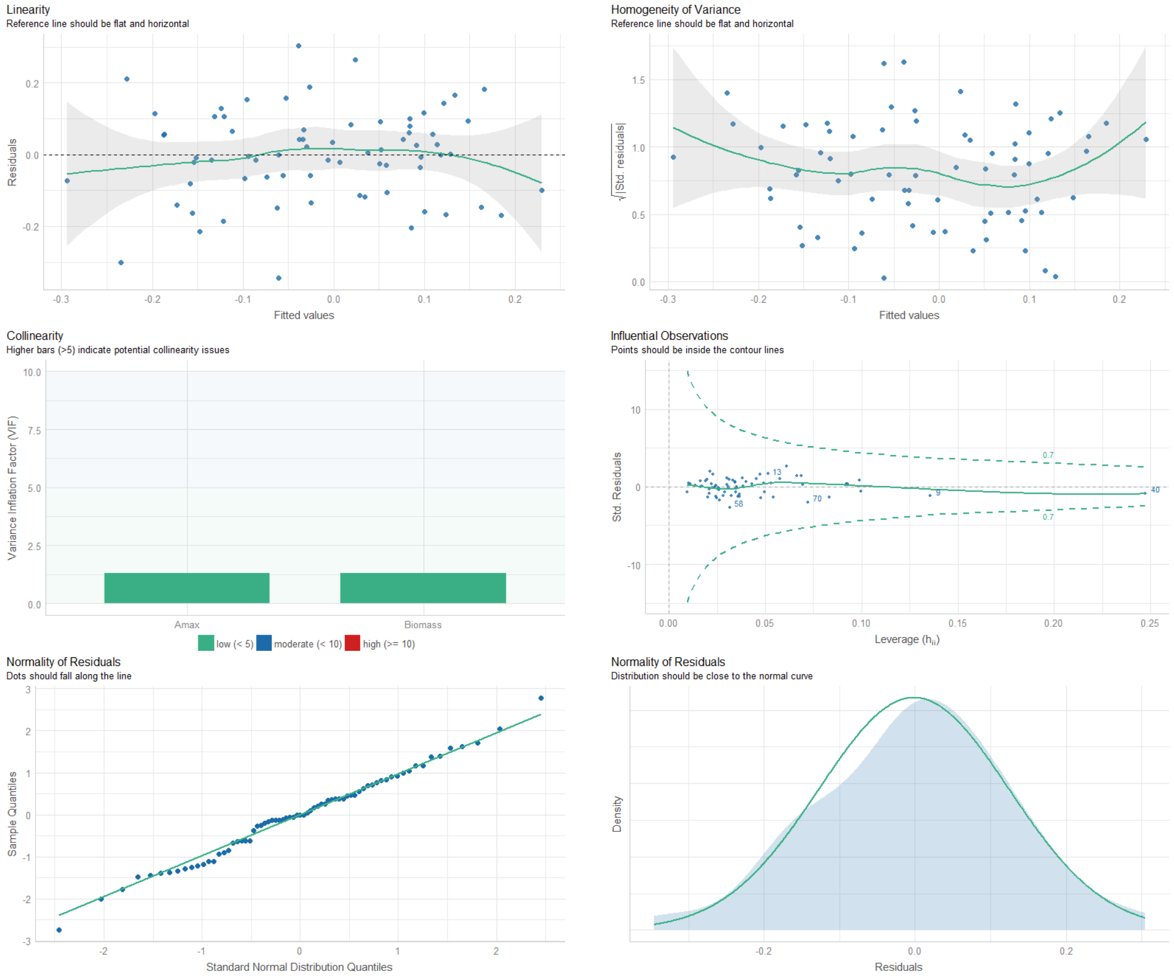

Supplementary Fig. S6 | Evaluation of the model using the calibration dataset (see Figure S5A). The SOC pool was modelled using the values found in the original articles of the data compilation. The predictors retained by the model were: maximal photosynthetic capacity of tree species (A_{max}) and stand biomass (Biomass). Other predictors were not significant.

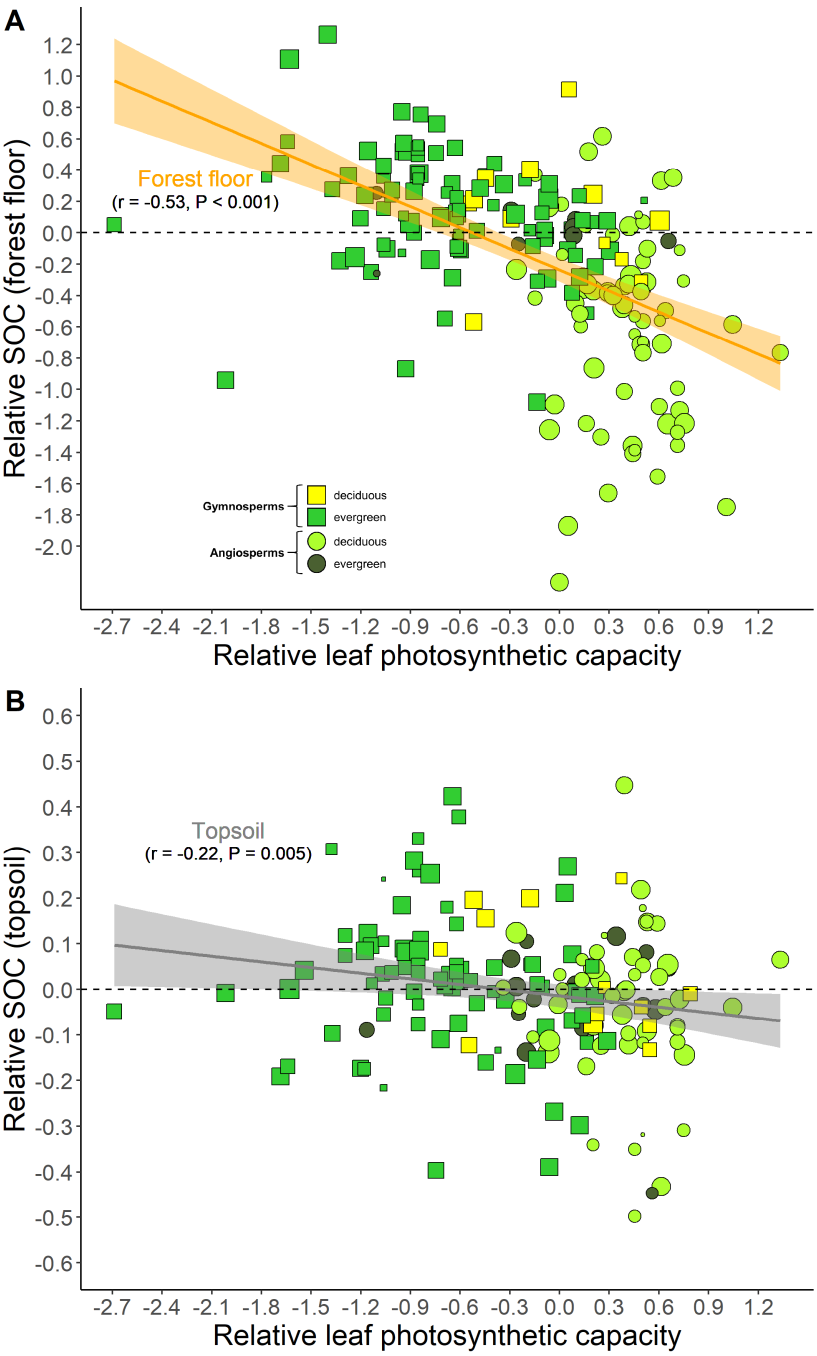

Supplementary Fig. S7 | Global influence of the photosynthetic capacity of tree species on SOC pools. SOC pools are: forest floor (*i.e.* the uppermost organic layer supplied by litterfall; panel A), and topsoil (*i.e.* upper layer of mineral soil with an Equivalent Soil Mass of 3000 Mg ha^{-1}; panel B). Values are normalised (see Methods). Linear regressions were fitted (level of confidence of the error band = 0.95). The symbol size is proportional to data reliability (see Methods), which was taken into account as a weighting factor in the regression.

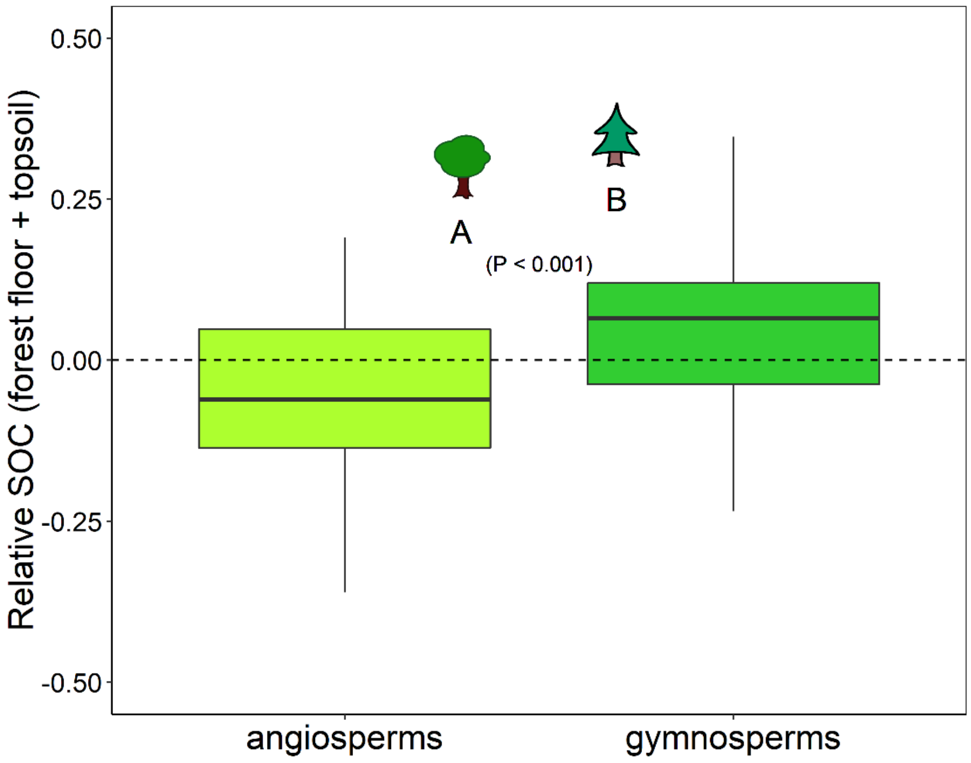

Supplementary Fig. S8 | Soil organic carbon under gymnosperms and angiosperms. Values are normalised (see Methods). The difference was tested with a pairwise *t*-test (*F* value = 16.76). Values: n = 68 pairs. Boxplots represent the median, the first and third quartiles, and 1.5 × the inter-quartile range. The difference between the two groups was tested with a pairwise t-test (two-sided).

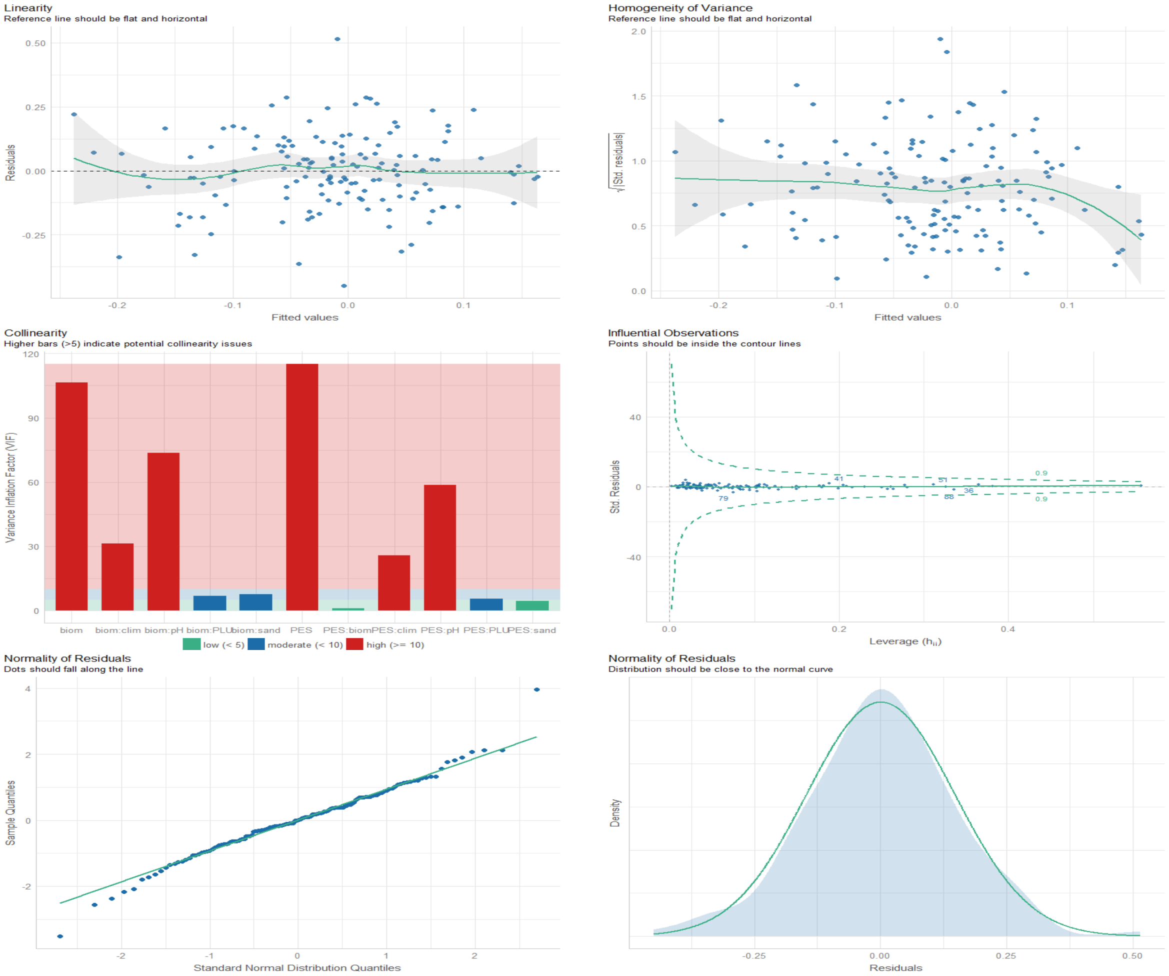

Supplementary Fig. S9 | Evaluation of the model based on imputed values, including past land-use (see Figure 4A). The SOC pool was modelled using imputed values based on a PCA approach (see Methods). The predictors were: an integrated value of the functional traits constituting the plant economics spectrum (PES), stand biomass (Biomass), a climatic index (f_{climate}), soil sand content, soil pH, and past land-use (PLU). The tested model was as follows:

$$\text{SOC} \sim (\text{PES} \times \text{Biomass}) + \{(\text{PES} + \text{Biomass}) : (f_{\text{climate}} + \text{Sand} + \text{pH} + \text{PLU})\}$$

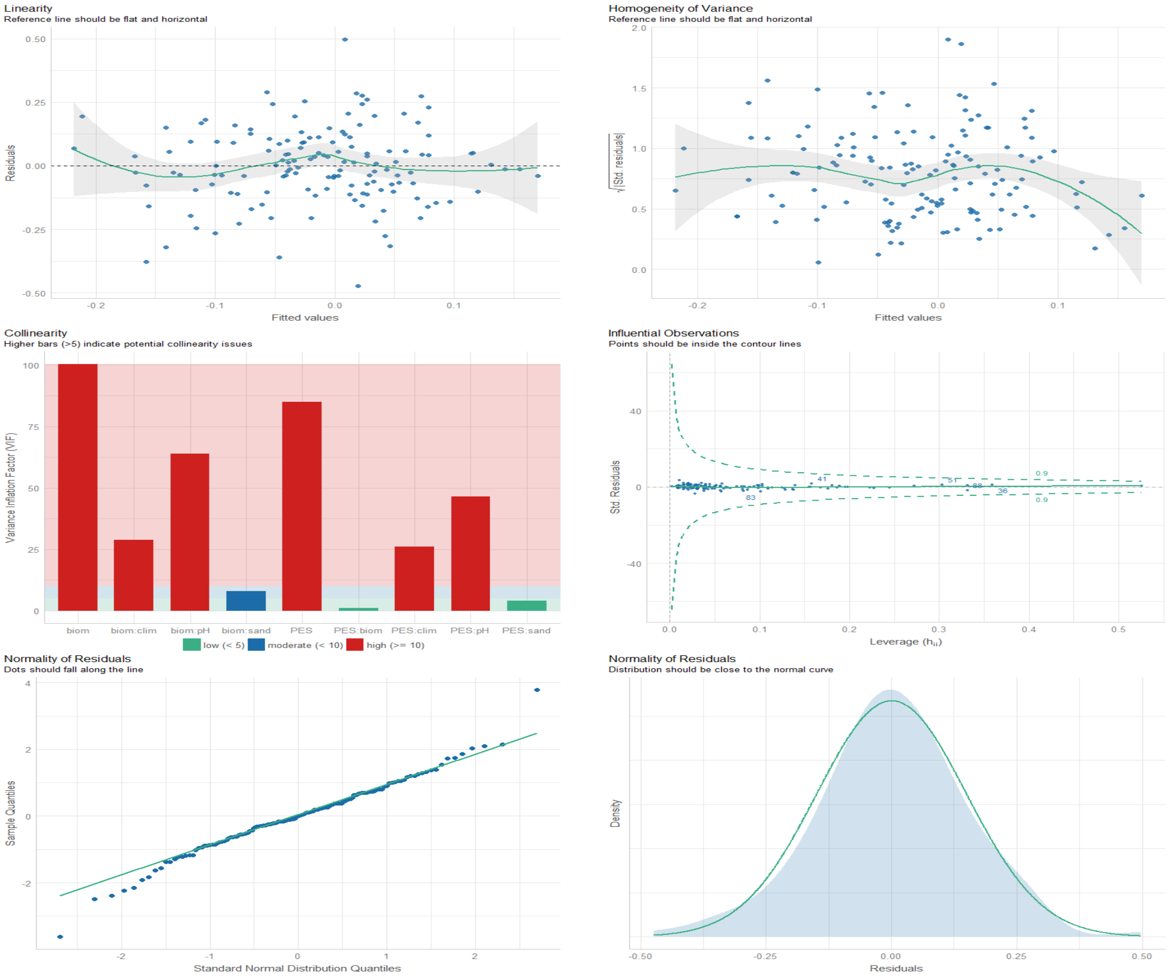

Supplementary Fig. S10 | Evaluation of the model based on imputed values, without past land-use (see Figure 4B). The SOC pool was modelled using imputed values based on a PCA approach (see Methods). The predictors were: an integrated value of the functional traits constituting the plant economics spectrum (PES), stand biomass (Biomass), a climatic index (f_{climate}), soil sand content, and soil pH. The tested model was as follows:

$$\text{SOC} \sim (\text{PES} \times \text{Biomass}) + \{(\text{PES} + \text{Biomass}) : (f_{\text{climate}} + \text{Sand} + \text{pH})\}$$

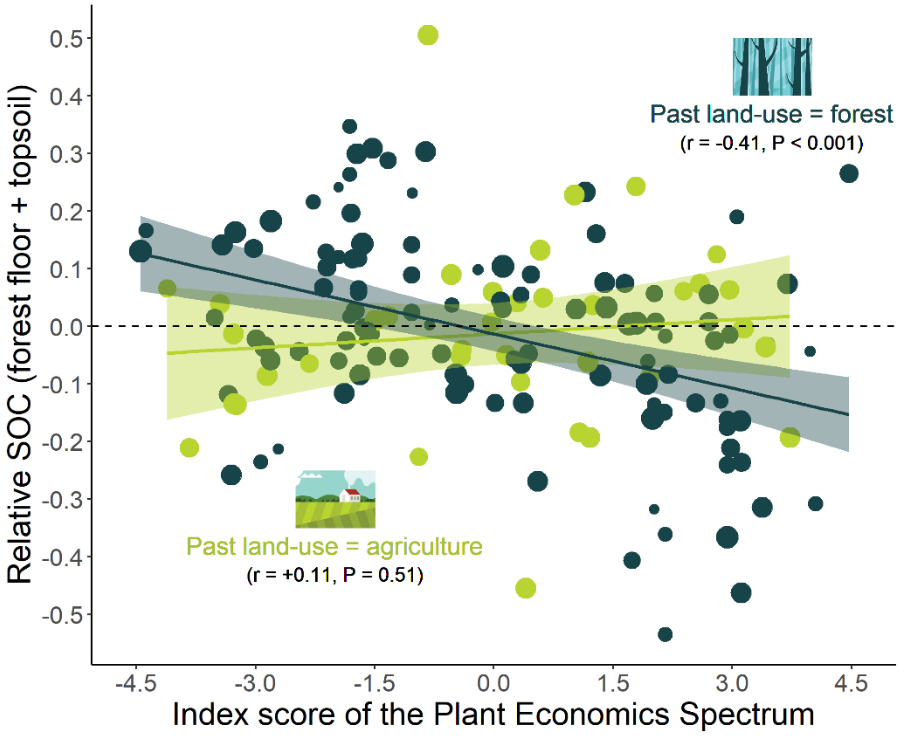

Supplementary Fig. S11 | Modulation of the imprint of tree species on forest SOC by past land-use and site fertilisation. Different colours indicate different past land-use of the studied forest. "agriculture" (green symbols) includes mainly grasslands, but also a few croplands and land treated with inorganic fertilisers; "forest" (dark grey symbols) includes mainly forests, but also a few shrublands. Values are normalised (see Methods). Linear regressions were fitted (level of confidence of the error band = 0.95). The symbol size is proportional to data reliability (see Methods), and regressions take it into account as a weighting factor.

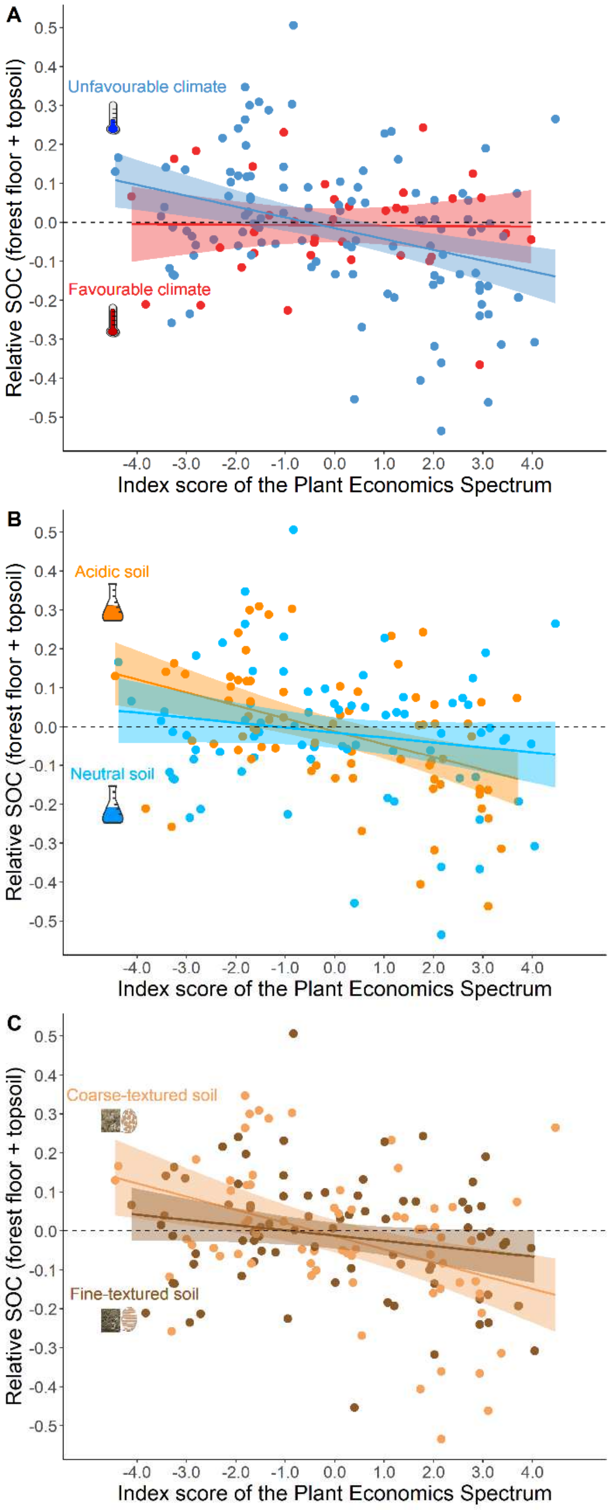

Supplementary Fig. S12 | Modulation of the imprint of tree species on SOC by climate and soil properties. Graphs show the relationships between the index score of the Plant Economics Spectrum and SOC pool in interaction with climate (A), soil texture (B), and soil acidity (C). Values are normalised values (see Methods). Linear regressions take data reliability into account as a weighting factor. Categories are based on threshold values close to the median values of (A) $f_{climate}$ = 0.35 (unitless), (B) soil pH = 5.0, (C) soil sand content = 500 mg g^{-1}. Low values, and high, values of $f_{climate}$ indicate respectively unfavourable climatic conditions (cold and/or dry), and favourable climatic conditions (warm and wet). Linear regressions were fitted (level of confidence of the error band = 0.95).

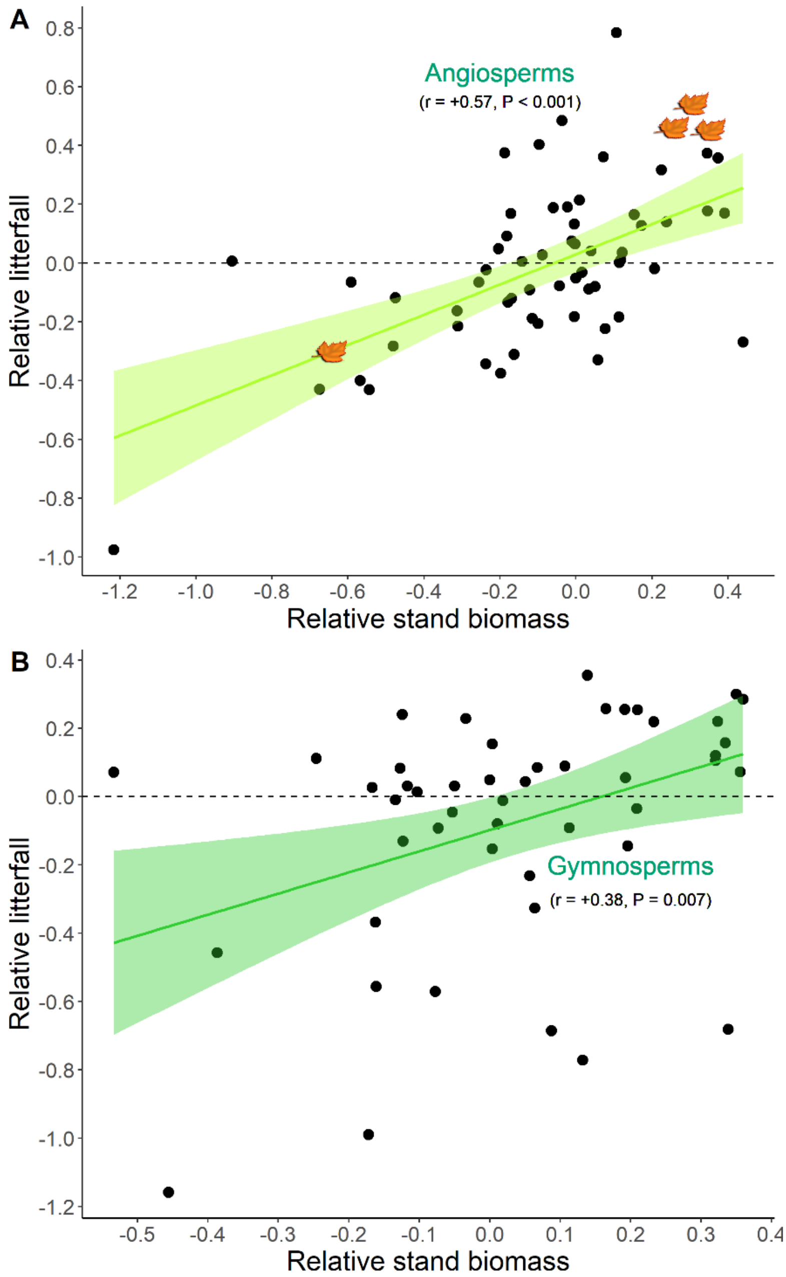

Supplementary Fig. S13 | Influence of stand aboveground biomass on stand litterfall flux. Values are normalised (see Methods). Linear regressions were fitted (level of confidence of the error band = 0.95).

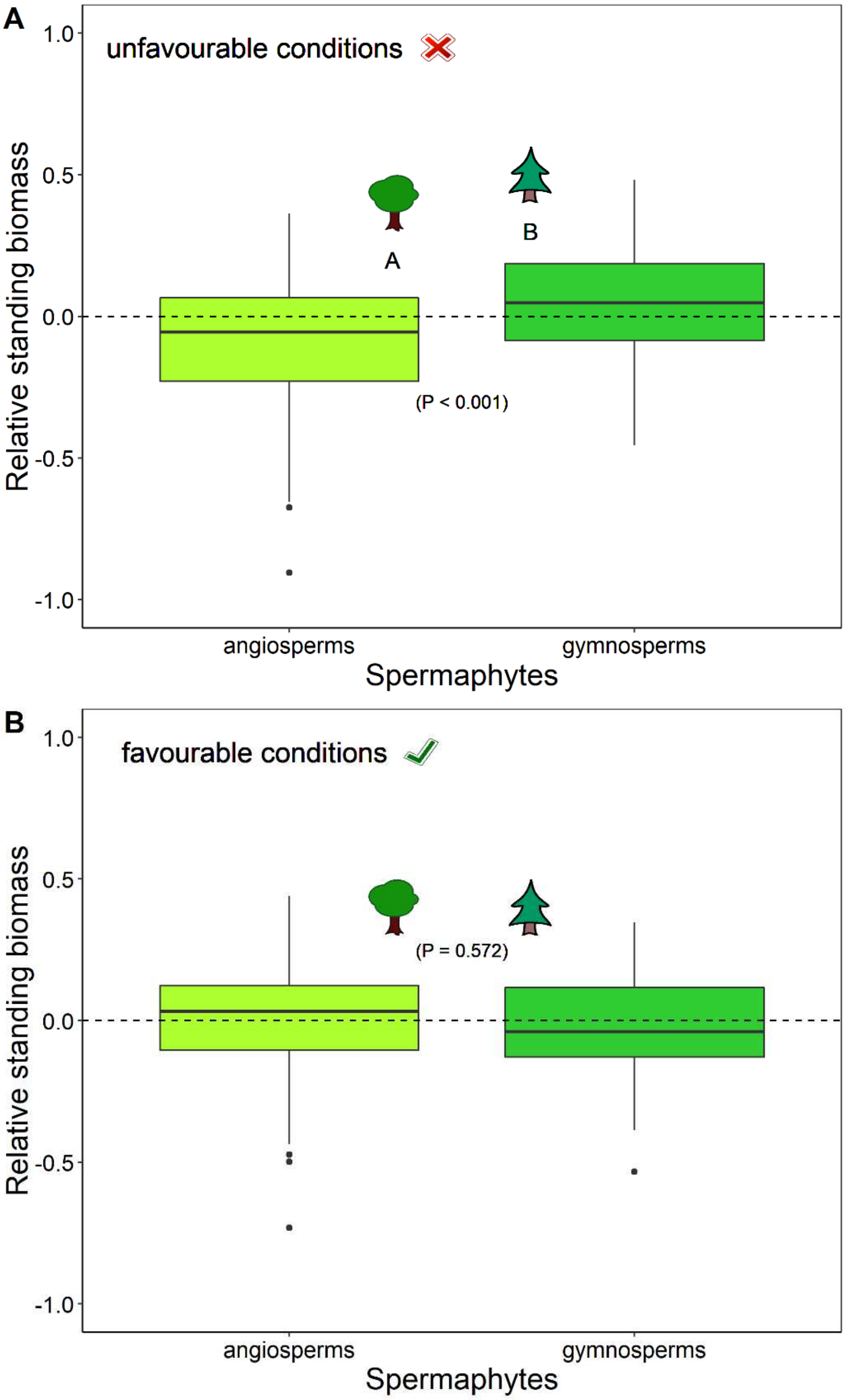

Supplementary Fig. S14 | Aboveground stand biomass in gymnosperm and angiosperm forests. Unfavourable and favourable climatic conditions were defined based on an index of potential biological activity ($f_{climate}$; see Methods) with 0.35 as a threshold value. Values: n = 189 and 67 pairs for panels A and B. W value = 3134 and 459.5 for panels A and B, respectively (Wilcoxon rank sum test; two sided). Values are normalised (see Methods). Boxplots represent the median, the first and third quartiles, and 1.5 × the inter-quartile range.

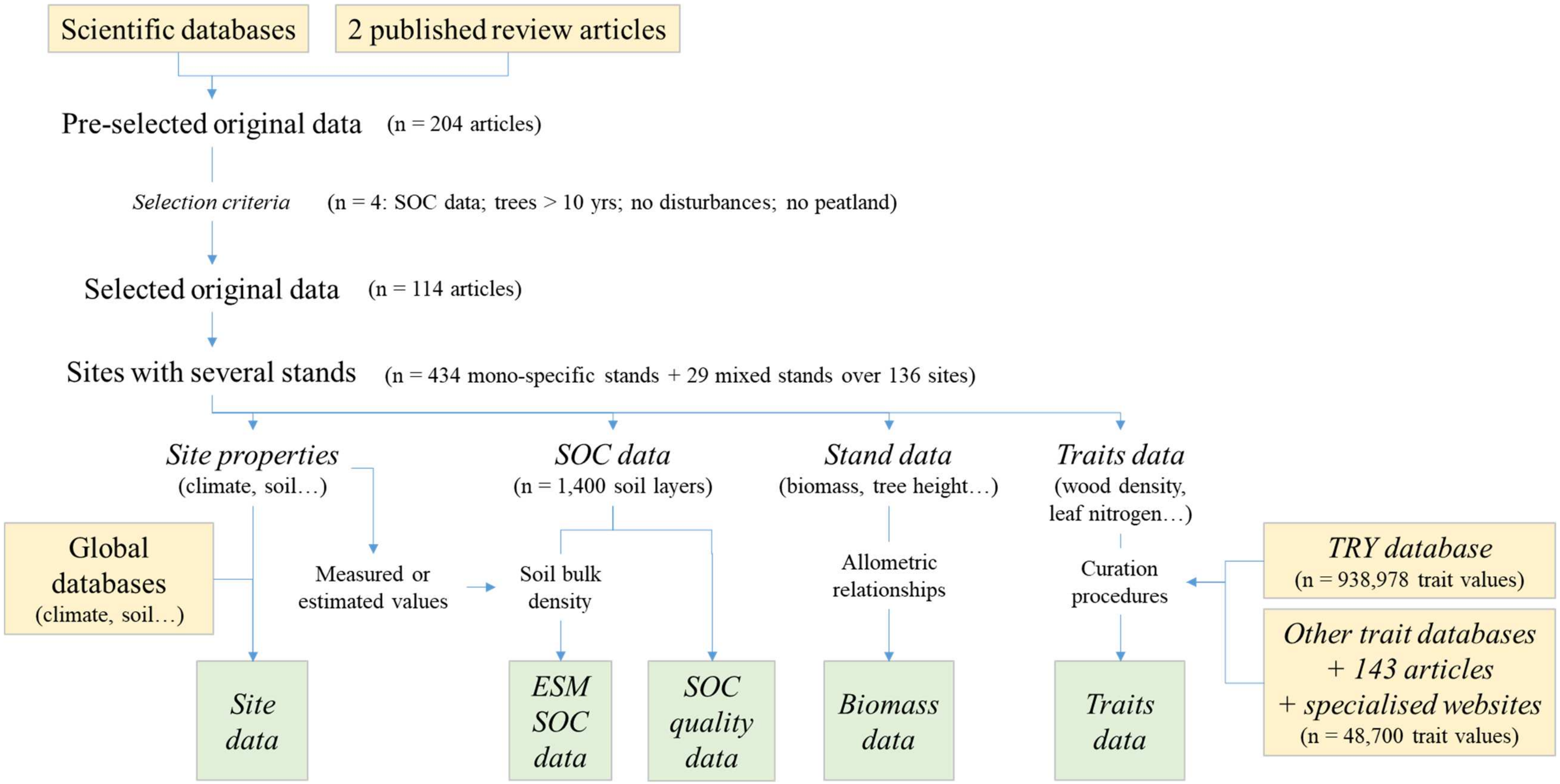

Supplementary Fig. S15 | Data flow of the study. Yellow boxes and green boxes indicate the sources of original data and the final datasets, respectively.

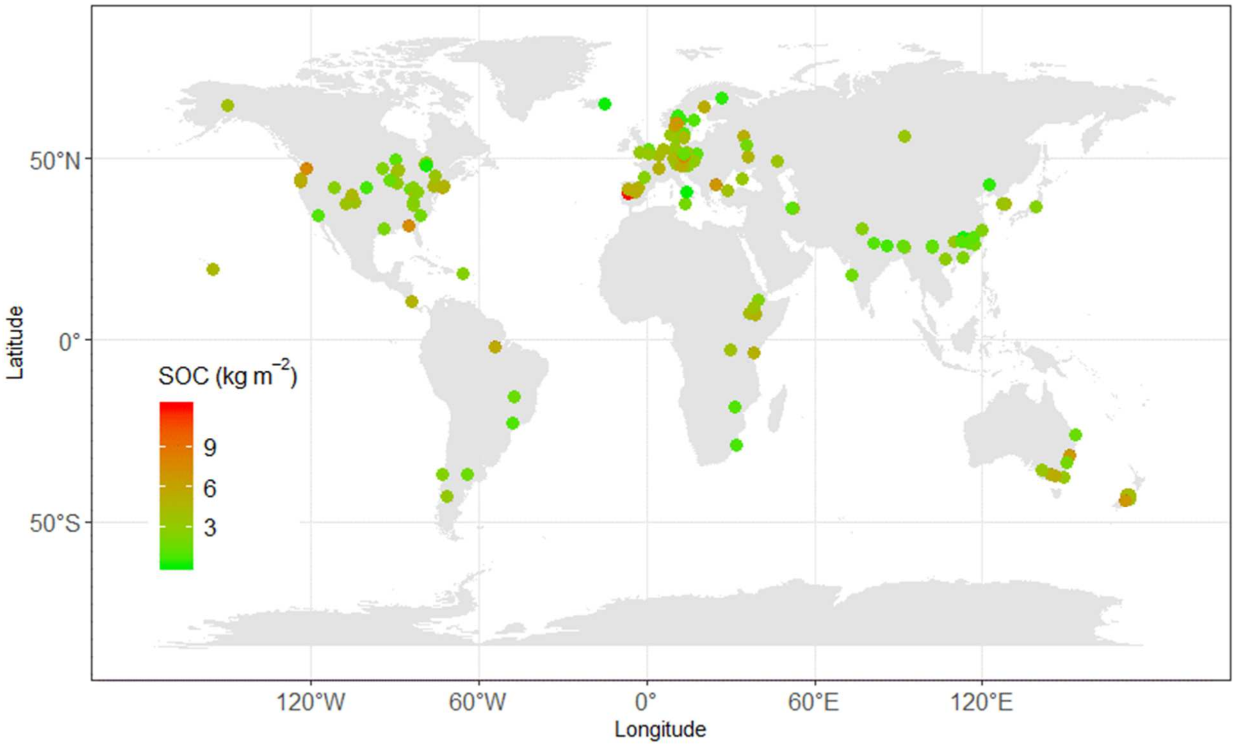

Supplementary Fig. S16 | Map of study site locations. A few sites had no geographical coordinates and are located approximately. The colours indicate the SOC pool size in the upper part of the soil mineral layer (for an Equivalent Soil Mass of 1,000 Mg of soil per hectare).

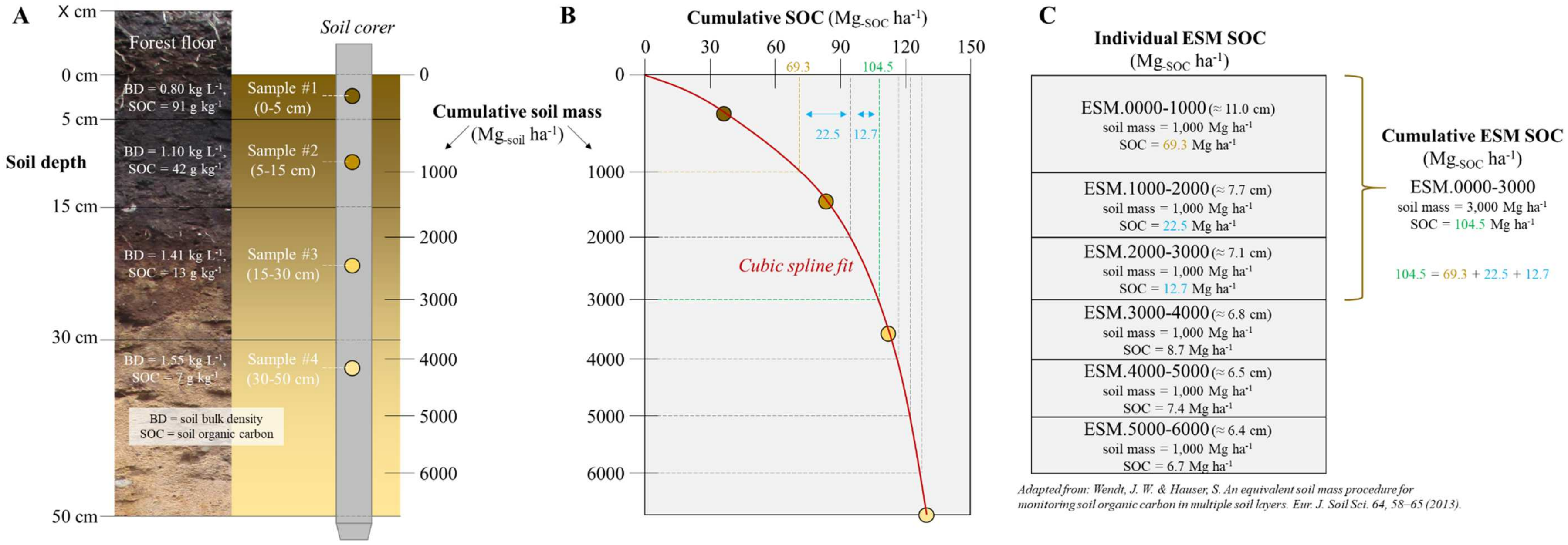

Supplementary Fig. S17 | Equivalent Soil Mass calculation. Fictive example of the way original SOC values were calculated in Equivalent Soil Mass (ESM) values. In the panel (A), a soil profile was sampled down to 50 cm and split in four layers (0-5 cm, 5-15 cm, 15-30 cm, and 30-50 cm; circles coloured from dark brown to light yellow). The right vertical axis indicated the cumulative soil mass of the profile (calculated based on volume and bulk density of the sampled layers). Having the mass and the SOC content value of the soil layers, it enabled to calculate the SOC pool of each layer. In the panel (B), the SOC pool values (*x* axis) were represented in a cumulative way (first the layer 0-5 cm [dark brown circle], then {layer 0-5 cm + layer 5-15 cm} [light brown circle], and so on), and plotted versus the cumulative soil mass (*y* axis). Then, a regression (cubic spline) was fit (red line). This regression was used to estimate the SOC pool of layers of equivalent soil mass (ESM, in Mg_{-soil} ha^{-1}): the topsoil layer of 1000 Mg_{-soil} ha^{-1} contained 69.3 Mg_{-SOC} ha^{-1}, the second layer of 1000 Mg_{-soil} ha^{-1} contained 22.5 Mg_{-SOC} ha^{-1}, the third layer of 1000 Mg_{-soil} ha^{-1} contained 12.7 Mg_{-SOC} ha^{-1}, and so on. The panel (C) indicated the final data. In our study, we retained as main studied soil layer the cumulated value of the three uppermost ESM individual layers (*i.e.* having an ESM of 3000 Mg_{-soil} ha^{-1}). In this example, this soil layer (named ESM.0000-3000) had a cumulative ESM SOC value of 104.5 Mg_{-SOC} ha^{-1}.

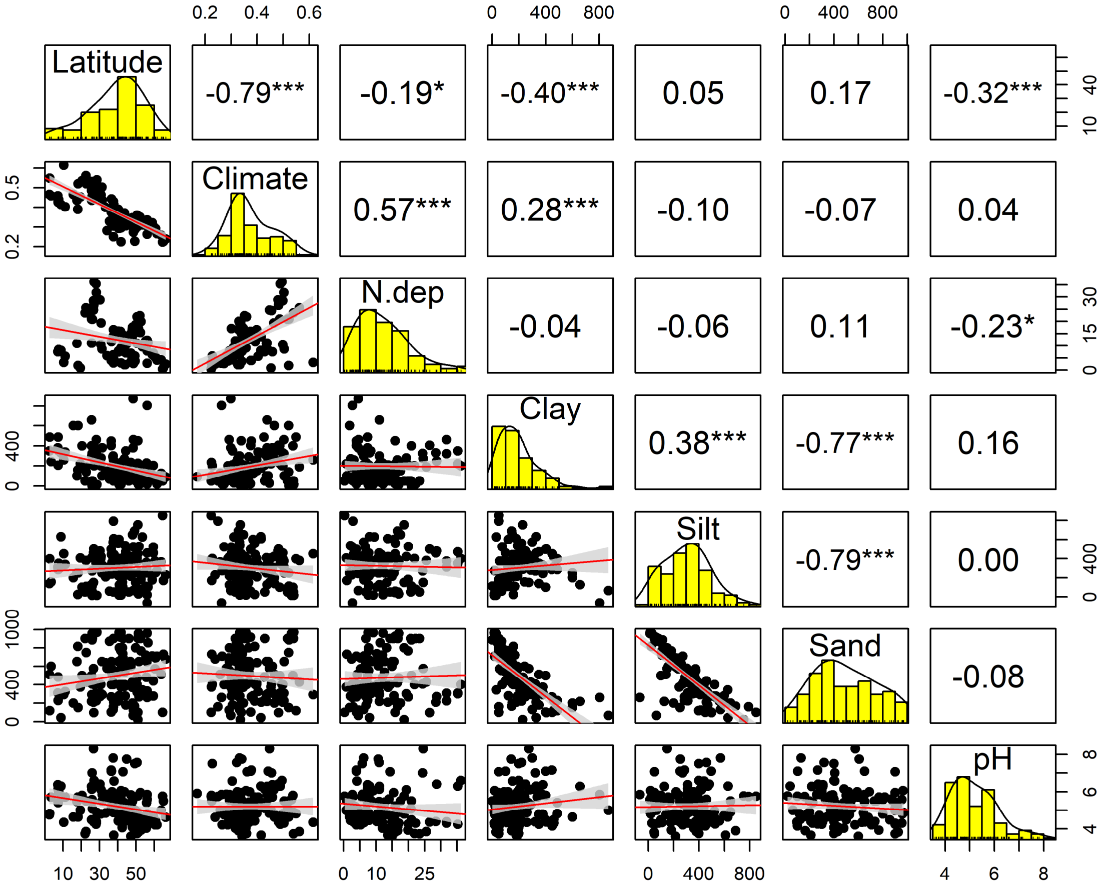

Supplementary Fig. S18 | Relationships among the site properties. Latitude (°); Climate = $f_{climate}$ (climate factor index; [0-1]; see Methods); N.dep = nitrogen atmospheric deposition (kg-N ha^{-1} yr^{-1}); Clay, Silt, and Sand = particle size fractions of soils (mg g^{-1}); pH = soil pH (unitless). The matrix shows the results of Spearman's rank correlation coefficients. The symbols *, **, and *** indicate correlations with P values respectively as follows: $P < 0.05$, $P < 0.01$, and $P < 0.001$.

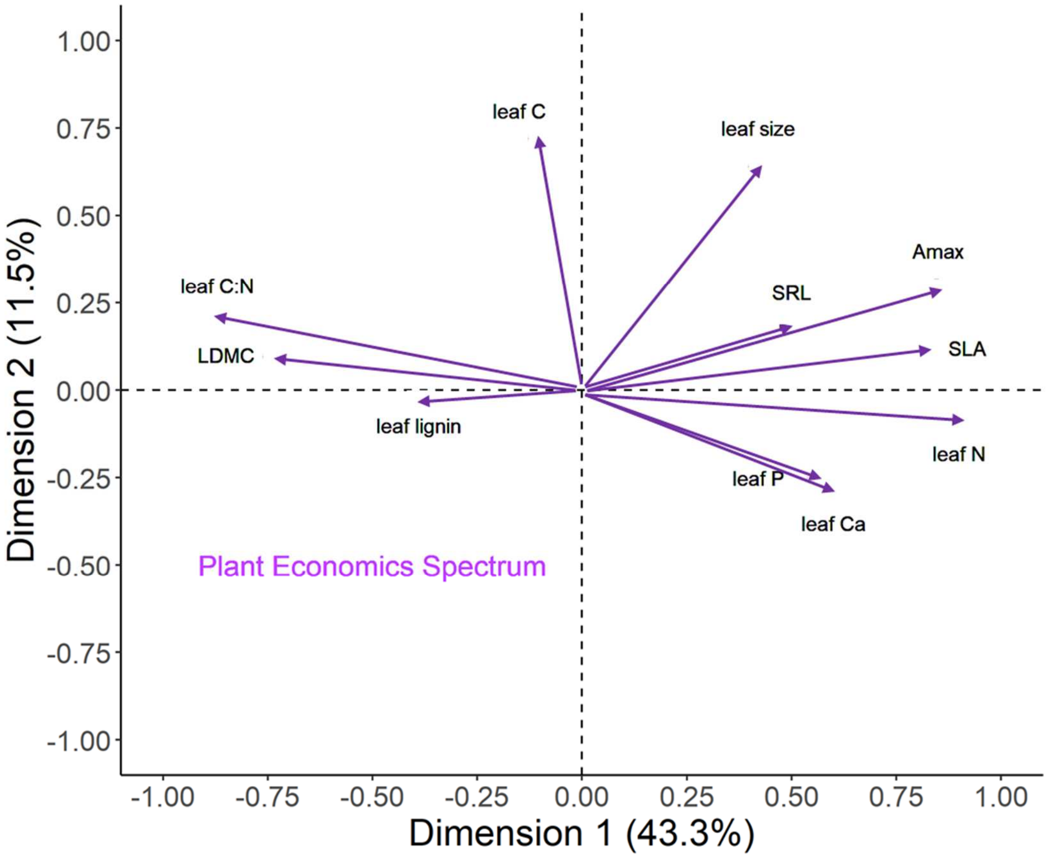

Supplementary Fig. S19 | Principal Component Analysis used to produce the index score of the Plant Economics Spectrum (PES). The functional traits of all the studied tree species were used to generate a Principal Component Analysis (PCA). For full explanation, see the section "*Data collection: plant functional traits*" in Methods. The final value of the index score of the PES was the coordinate value on the first axis ("Dimension 1").

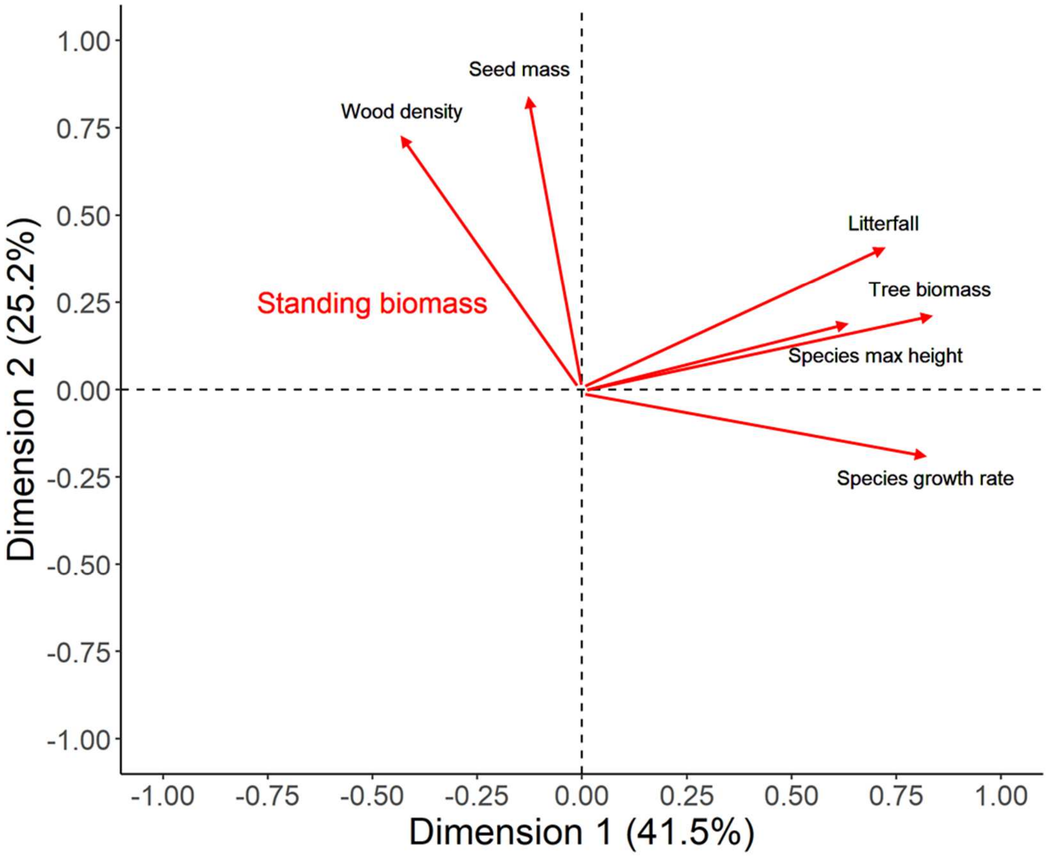

Supplementary Fig. S20 | Principal Component Analysis used to produce the index score of the forest standing biomass. The variables that were directly related to biomass ("litterfall" and "tree biomass"), and functional traits known to be related to biomass or growth ("max height", "growth rate", "seed mass", "wood density") were used to generate a Principal Component Analysis (PCA). For full explanation, see the section "*Data collection: plant functional traits*" in Methods. The final value of the index score of the standing biomass was the coordinate value on the first axis ("Dimension 1").

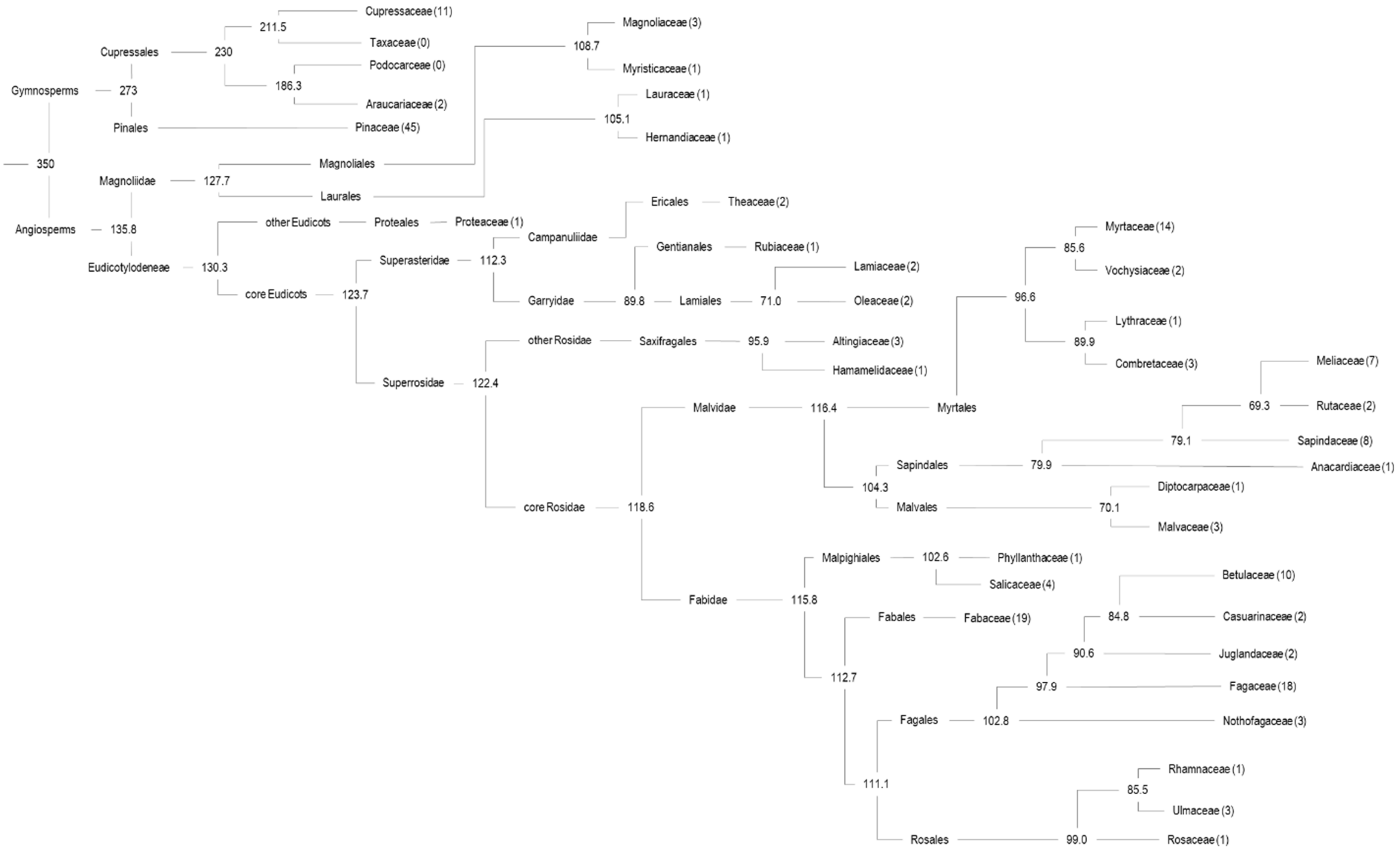

Supplementary Fig. S21 | Phylogenetic tree of the species of the study. Values on the phylogenetic tree are the distances in millions of years (Myr). For the sake of clarity, the stem lengths are not proportional to the phylogenetic distances. Values in brackets next to family names are the number of species present in our dataset.

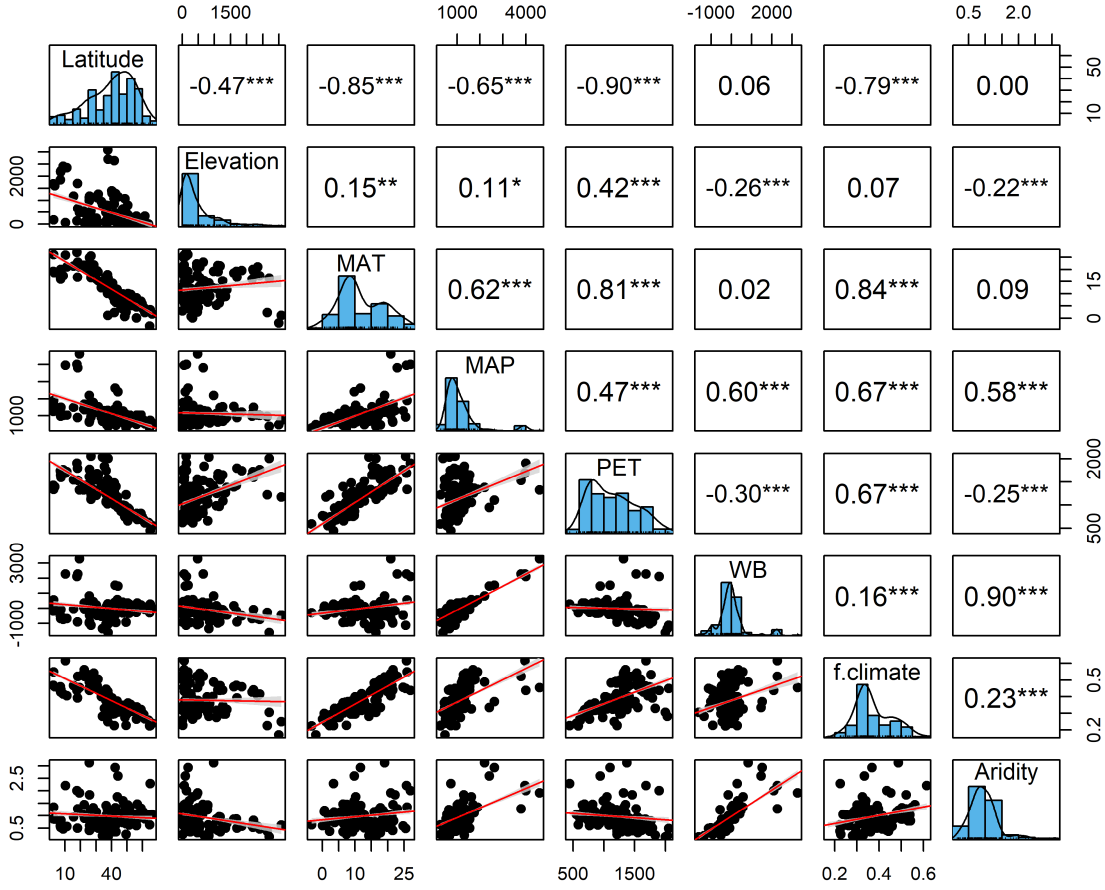

Supplementary Fig. S22 | Relationships among climatic descriptors. MAT = mean annual temperature (°C); MAP = mean annual precipitation (mm yr^{-1}); PET = potential evapotranspiration (mm yr^{-1}); WB = water balance (MAP-PET difference); $f_{climate}$ (climate factor index; [0-1]; see Methods). The matrix shows the results of Spearman's rank correlation coefficients. The symbols *, **, and *** indicate correlations with P values respectively as follows: P < 0.05, P < 0.01, and P < 0.001.

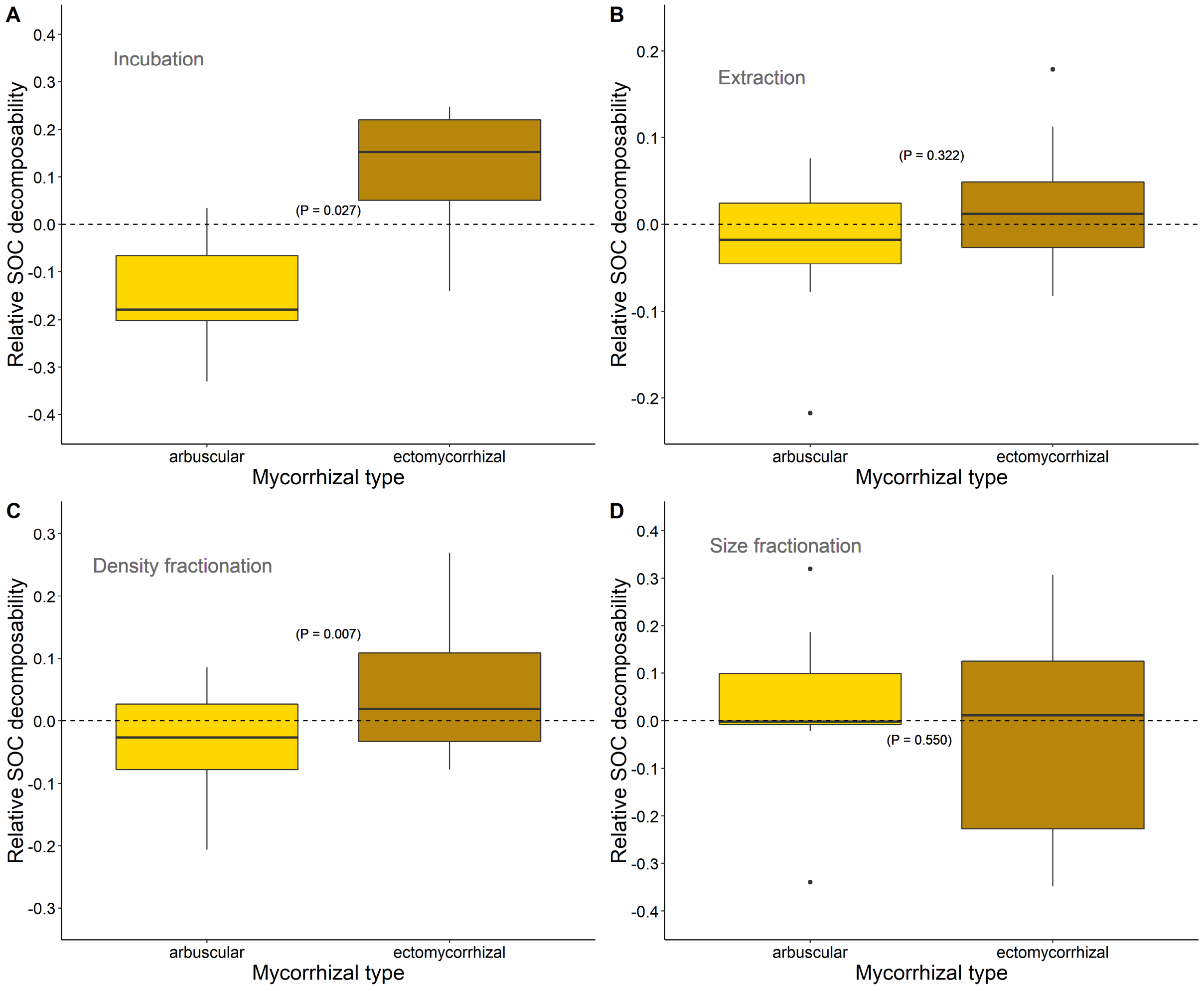

Supplementary Fig. S23 | Differences of SOC decomposability between arbuscular mycorrhizal tree species and ectomycorrhizal tree species, as influenced by the method used to quantify the SOC decomposability. Values (n=20, 28, 36 and 27 pairs, for the panels A, B, C and D, respectively) show the SOC decomposability, which is the opposite of SOC stability. Values are normalised. Boxplots represent the median, the first and third quartiles, and 1.5 × the inter-quartile range. Significant differences were tested with pairwise t-test or Wilcoxon test (two-sided), depending on data structure.

Supplementary Table S1 | Influence of plant functional traits and stand properties on SOC pools. N = number of values; r = Spearman correlation coefficient; *P* value. Leaf A_{max}: leaf photosynthetic maximum capacity; LDMC: leaf dry matter content; SLA and SRL: specific leaf area and specific root length; Leaf C, lignin, N, P, and Ca: leaf content (mass basis). Correlations were tested with the Spearman's rank correlation coefficients.

Predictor	*Forest floor +Topsoil*			*Forest floor*			*Topsoil*		
	n	r	*P*	n	r	*P*	n	r	*P*
Leaf A_{max}	(75)	-0.65	<0.001	(139)	-0.67	<0.001	(107)	-0.25	0.009
LDMC	(33)	0.56	0.001	(58)	0.72	<0.001	(40)	n.s.	n.s.
SLA	(85)	-0.54	<0.001	(163)	-0.60	<0.001	(127)	n.s.	n.s.
SRL	(40)	-0.60	<0.001	(90)	-0.24	0.021	(53)	-0.25	0.067
Wood density	(149)	-0.30	<0.001	(227)	-0.35	<0.001	(256)	-0.11	0.087
Leaf C	(149)	0.16	0.057	(227)	0.22	0.001	(256)	n.s.	n.s.
Leaf lignin	(110)	n.s.	n.s.	(171)	0.18	0.020	(147)	n.s.	n.s.
Leaf N	(108)	-0.39	<0.001	(186)	-0.57	<0.001	(164)	n.s.	n.s.
Leaf C:N ratio	(108)	0.39	<0.001	(186)	0.54	<0.001	(164)	n.s.	n.s.
Leaf P	(103)	n.s.	n.s.	(166)	-0.23	0.002	(159)	n.s.	n.s.
Leaf Ca	(74)	-0.33	0.004	(126)	-0.46	<0.001	(125)	n.s.	n.s.
Leaf Size	(110)	-0.31	0.001	(175)	-0.34	<0.001	(148)	-0.13	0.113
Seed mass	(122)	-0.30	0.001	(200)	-0.29	<0.001	(209)	n.s.	n.s.
Tree max height	(145)	0.15	0.074	(223)	0.25	<0.001	(225)	n.s.	n.s.
Stand biomass	(113)	0.25	0.007	(160)	0.24	0.002	(164)	0.14	0.082
Stand litterfall	(51)	0.35	0.012	(81)	0.16	0.147	(78)	n.s.	n.s.

Supplementary Table S2 | Models predicting the SOC content. The tested models were all of the basic form:

SOC ~ (χ + Biomass) + { (χ + Biomass) : (Climate + Sand + pH + PLU) }

with:

":" indicates the tested interactions

SOC = soil content in organic carbon

χ = main predictor (A_{max} or PES, depending on the model)

A_{max} = Leaf maximum photosynthetic capacity (relative values)

PES = score value of the Plant Economics Spectrum (imputed values)

Biomass = stand biomass (relative values)

Climate = climatic descriptor of the sites ($f_{climate}$)

Sand = soil sand content

pH = soil pH value

PLU = site past land-use (used only in two of the four models)

Model χ	Model PLU		Retained predictors	AIC	r^2	Adj. r^2
A_{max}	yes	(n=2)	*A_{max} , Biomass*	-86.2	0.500	0.485
A_{max}	no	(n=2)	*A_{max} , Biomass*	-86.2	0.500	0.485
PES	yes	(n=5)	*PES , PES:Climate , PES:pH , PES:PLU , Biomass :Sand*	-130.4	0.207	0.180
PES	no	(n=4)	*PES , PES:Climate , PES:pH , Biomass :Sand*	-122.0	0.201	0.178

Supplementary Table S3 | Mean values of functional traits of several tree plant functional types. AM and EC: arbuscular mycorrhizal tree species and ectomycorrhizal tree species; leaf A_{max}: leaf photosynthetic maximum capacity; leaf N: leaf content in nitrogen; LDMC: leaf dry matter content; SLA and SRL: specific leaf area and specific root length; max height: tree maximum height. Values are means ± 1 standard error. Values followed by different letters are significantly different (*P* < 0.05; tested with Bonferroni test, Wilcoxon test, or Mann-Whitney, depending on data structure). Groups with less than 5 values were not included in the tests (¤). Groups with less than 3 values are not presented (n.p.).

Plant functional type	n	Leaf A_{max} (μmol g^{-1} s^{-1})	Leaf N (mg g^{-1})	LDMC (g g^{-1})	SLA (mm^2 mg^{-1})	SRL (m g^{-1})	Wood density (kg L^{-1})	Max height (m)
Angiosperm - AM	21-65	0.15±0.02[b]	21.8±1.3[b]	0.36±0.01[ab]	17.5±2.0[c]	47.3±7.0[b]	0.54±0.02[b]	30.6±1.8[a]
Angiosperm - EC	14-34	0.15±0.02[b]	21.2±1.0[b]	0.35±0.02[a]	15.0±1.0[bc]	47.0±5.2[b]	0.62±0.02[c]	33.1±2.5[a]
Gymnosperm - AM	2-13	0.04±0.01[a]	12.2±1.0[a]	0.37±0.04[¤]	5.8±1.2[a]	n.p.	0.47±0.02[a]	46.0±5.7[b]
Gymnosperm - EC	16-45	0.05±0.01[a]	12.6±0.7[a]	0.42±0.02[b]	7.6±0.6[ab]	24.0±2.3[a]	0.46±0.01[a]	49.1±3.2[b]
Broadleaf deciduous	32-68	0.17±0.02[b]	23.0±0.7[b]	0.35±0.01[a]	18.4±1.6[c]	48.3±5.2[b]	0.55±0.02[b]	33.7±1.5[a]
Broadleaf evergreen	10-50	0.17±0.03[b]	19.9±1.9[b]	0.36±0.02[a]	13.4±1.1[b]	63.8±9.9[b]	0.61±0.02[c]	35.0±3.0[a]
Needleleaf deciduous	1-4	0.11±0.04[¤]	22.1±2.1[¤]	n.p.	11.7±1.5[¤]	n.p.	0.46±0.02[¤]	47.8±3.4[¤]
Needleleaf evergreen	19-55	0.05±0.01[a]	11.6±0.4[a]	0.42±0.02[b]	7.0±0.6[a]	23.7±2.4[a]	0.47±0.01[a]	46.8±3.1[b]
Angiosperm N fixer	5-26	0.20±0.04[a]	31.3±2.5[b]	0.35±0.03[a]	21.9±4.7[b]	68.1±20.7[a]	0.58±0.03[a]	23.4±2.1[a]
Angiosperm non-fixer	57-97	0.16±0.02[a]	19.3±0.6[a]	0.36±0.01[a]	15.1±0.7[a]	48.7±4.0[a]	0.58±0.01[a]	36.2±1.7[b]

Supplementary Table S4 | List of the variables used in the study.
H1, H2, H3 = hypotheses tested in the study (see main text); PES = Plant Economics Spectrum; SOC = Soil Organic Carbon;

Variable	Unit	Origin	Use	Hypothesis
Data weight (Wdata)	[0-1]	classified based on original publication (see Methods)	Used to give a statistical weight to SOC data (based on the strength of the study design)	H1, H2
Leaf C	mg/g	original publication, or trait database, or estimated (see Methods)	Trait of the PES, used as possible predictor of SOC	H1
Leaf C:N ratio	_	calculated		
Leaf N	mg/g	original publication, or trait database		
Leaf N:P ratio	_	calculated		
Leaf P	mg/g	original publication, or trait database		
Leaf K	mg/g	original publication, or trait database		
Leaf Ca	mg/g	original publication, or trait database		
Leaf Mg	mg/g	original publication, or trait database		
Leaf Mn	mg/g	original publication, or trait database		
Leaf Lignin	mg/g	original publication, or trait database		
Leaf Dry Matter Content (LDMC)	g/g	trait database		
Leaf area	mm2	trait database		
Specific Leaf Area (SLA)	mm2/mg	original publication, or trait database		
Leaf type (shape)	B=broadleaf, N=needleleaf	original publication, or trait database, or specialised websites		
Leaf phenology	D=deciduous, E=evergreen	original publication, or trait database, or specialised websites		
Leaf photosynthetic capacity (A_{max})	µmol/g/s	trait database		
Leaf stomatal conductance	mmol/m2/s	trait database	Initially used (1) to confirm that our dataset was consistent with the PES and (2) for the gap filling procedure (see Methods)	_
Leaf photosynthesis carboxylation capacity	µmol/g/s	trait database		
Litterfall C	mg/g	trait database		
Litterfall C:N ratio	_	calculated		
Litterfall N	mg/g	original publication, or trait database		
Litterfall P	mg/g	original publication, or trait database		

Litterfall K	mg/g	original publication, or trait database		
Litterfall Ca	mg/g	original publication, or trait database		
Litterfall Mg	mg/g	original publication, or trait database		
Litterfall Mn	mg/g	original publication, or trait database		
Fine root specific length (SRL)	m/g	trait database	Trait of the PES, used as possible predictor of SOC	H1
Fine root C	mg/g	original publication, or trait database	Initially used (1) to confirm that our dataset was consistent with the PES and (2) for the gap filling procedure (see Methods)	_
Fine root C:N ratio	_	calculated		
Fine root N	mg/g	original publication, or trait database		
Fine root P	mg/g	original publication, or trait database		
Fine root K	mg/g	original publication, or trait database		
Fine root Ca	mg/g	original publication, or trait database		
Fine root Mg	mg/g	original publication, or trait database		
Fine root diameter	mm	trait database		
Plant phylogenetic distance	Myr	calculated (see Methods)	Used as a possible proxy of functional distance between two species	H1
Plant phylogeny	spermaphyte, … , family, genus, species	wikispecies	Used as a categorical variable (e.g. angiosperms vs gymnosperms) that may explain SOC	H1
Plant mycorhizal type	AM=arbuscular, EC=ecto, MIX=mixed, NO=no symbiosis	trait database, or specialised publications (see Methods)	Used as a categorical variable that may explain SOC	H1
Plant N fixation	N=no, Y=yes	original publication, or determined based on species genus		
Plant growth rate	classes=1-3	original publication, or trait database, or specialised websites	Used as a proxy of the species ecological strategy	H1
Plant tolerance to drought	classes=1-5	trait database, or specialised publications, or specialised website (see Methods)		
Plant tolerance to waterlogging	classes=1-5	trait database, or specialised publications, or specialised website		
Plant xylem cavitation vulnerability (P50)	Mpa	trait database, or specialised publications, or specialised website		
Plant max height	m	original publication, or trait database, or specialised websites	Trait of the PES, used as possible predictor of SOC	H1
Plant seed mass	mg/seed	trait database, or specialised website		
Plant wood density	g/cm3	trait database, or specialised publications, or specialised website (see Methods)		

Site longitude	degrees	original publication	Used to extract data from global datasets	_
Site latitude	degrees	original publication	Used to evaluate the interactions between PES, SOC, and environmental conditions	H2
Site elevation	m above sea level	original publication, or global database (SRTM-NASA)		
Site mean annual precipitation (MAP)	mm/yr	original publication, or global database (WorldClim)		
Site mean annual temperature (MAT)	°C	original publication, or global database (WorldClim)		
Site Koppen climate	A=tropical, B=dry, C=temperate, D=snow, E=cold	global database (Kottet et al., 2006)		
Site aridity index	_	global database (WorldClim)		
Site potential evapotranspiration (PET)	mm/yr	global database (CGIAR)		
Site climatic conditions for biological activity ($f_{climate}$)	[0;1], From 0=unfavourable to 1=favourable	calculated based on latitude and mean monthly values of temperature and precipitation (Augusto et al., 2017)		
Site nitrogen atmospheric deposition	kg-N/ha/yr	global database (Vet et al., 2014)		
Site topography (class)	plain, hill, or mountain	original publication		
Site topography (value)	%	original publication		
Site past land-use	forest, grassland, savanna, tundra, wetland, other permanent vegetation, desert, cropland	original publication		
Site fertilisation history	N=no fertilisation, Y=at least one fertilisation application	original publication		
Site soil name	USDA soil classification	original publication		
Site soil parent material	acid, intermediate, mafic, calcareous (see Methods)	classified based on original publication (see Methods)		
Site mean value of topsoil texture (clay)	mg/g	calculated based on original publication (see Methods)		

Site mean value of topsoil texture (silt)	mg/g	calculated based on original publication		
Site mean value of topsoil texture (sand)	mg/g	calculated based on original publication		
Site mean value of topsoil SOC	mg/g	calculated based on original publication		
Site mean value of topsoil P-available content	µg/g	calculated based on original publication		
Site mean value of topsoil pH-water	–	calculated based on original publication		
Site mean value of topsoil base saturation	%	calculated based on original publication		
Stand age	yr	original publication	Used to evaluate the interactions between PES, SOC, and duration of the soil exposure to different tree species	H1
Stand aboveground biomass	Mg/ha	original publication, or calculated	Used a possible predictor of SOC	H1, H3
Stand aboveground growth	Mg/ha/yr	original publication		
Stand litterfall	Mg/ha/yr	original publication		
Stand fine root biomass	Mg/ha	original publication		
Stand fine root turnover	Mg/ha/yr	original publication		
Soil layer position (depth and thickness)	cm	original publication	Used to calculate SOC values following the Equivalent Soil Mass method	–
Soil bulk density	kg/dm3	original publication, or calculated		
Soil layer texture (clay)	mg/g	original publication, or estimated by global study (Hengl et al., 2017; Shangguan et al., 2014)	Used to (1) test the comparability among the stands of a given site, and (2) calculate the mean soil properties at the site scale	H2
Soil layer texture (silt)	mg/g	original publication, or estimated by global study (Hengl et al., 2017; Shangguan et al., 2014)		
Soil layer texture (sand)	mg/g	original publication, or estimated by global study (Hengl et al., 2017; Shangguan et al., 2014)		
Soil content in organic carbon (SOC)	mg/g	original publication, or calculated	Used to calculate the SOC values in ESM, which was the main studied variable	H1, H2, H3
Soil pool in organic carbon (SOC)	Mg/ha	original publication, or calculated		

Soil fraction of SOC stability	see Methods	original publication	Used as a proxy of the SOC stability	H1
Soil content in organic matter (SOM)	mg/g	original publication, or calculated	Used to calculate SOC values	–
Soil pool in organic matter (SOM)	Mg/ha	original publication, or calculated		
Soil content in total nitrogen (N-tot)	mg/g	original publication, or calculated	Used to calculate the mean soil properties at the site scale	H2
Soil pool in total nitrogen (N-tot)	Mg/ha	original publication, or calculated		
Soil C:N ratio	_	original publication, or calculated		
Soil content in P-available	µg/g	original publication		
Soil pH (water)	–	original publication, or estimated by global study (Hengl et al., 2017; Shangguan et al., 2014)		
Soil Cation Exchange Capacity (CEC)	cmol.c/kg	original publication		
Soil base saturation of the CEC	%	original publication, or estimated by global study (Hengl et al., 2017; Shangguan et al., 2014)		

Supplementary Table S5 | PRISMA abstract checklist.

Section and Topic	Item #	Checklist item	Reported (Yes/No)
**TITLE**			
Title	1	Identify the report as a systematic review.	Yes
**BACKGROUND**			
Objectives	2	Provide an explicit statement of the main objective(s) or question(s) the review addresses.	Yes
**METHODS**			
Eligibility criteria	3	Specify the inclusion and exclusion criteria for the review.	Yes
Information sources	4	Specify the information sources (e.g. databases, registers) used to identify studies and the date when each was last searched.	Yes
Risk of bias	5	Specify the methods used to assess risk of bias in the included studies.	Yes
Synthesis of results	6	Specify the methods used to present and synthesise results.	Yes
**RESULTS**			
Included studies	7	Give the total number of included studies and participants and summarise relevant characteristics of studies.	Yes
Synthesis of results	8	Present results for main outcomes, preferably indicating the number of included studies and participants for each. If meta-analysis was done, report the summary estimate and confidence/credible interval. If comparing groups, indicate the direction of the effect (i.e. which group is favoured).	Yes
**DISCUSSION**			
Limitations of evidence	9	Provide a brief summary of the limitations of the evidence included in the review (e.g. study risk of bias, inconsistency and imprecision).	Yes
Interpretation	10	Provide a general interpretation of the results and important implications.	Yes
**OTHER**			
Funding	11	Specify the primary source of funding for the review.	Yes
Registration	12	Provide the register name and registration number.	No

From: Page MJ, McKenzie JE, Bossuyt PM, Boutron I, Hoffmann TC, Mulrow CD, et al. The PRISMA 2020 statement: an updated guideline for reporting systematic reviews. BMJ 2021;372:n71. doi: 10.1136/bmj.n71
For more information: http://www.prisma-statement.org/

Supplementary Table S6 | PRISMA checklist.

Section and Topic	Item #	Checklist item	Location where item is reported
**TITLE**			
Title	1	Identify the report as a systematic review.	Methods (Data collection: soil organic carbon): lines 224-226
**ABSTRACT**			
Abstract	2	See the PRISMA 2020 for Abstracts checklist.	Dedicated file
**INTRODUCTION**			
Rationale	3	Describe the rationale for the review in the context of existing knowledge.	Introduction (lines 40-47)
Objectives	4	Provide an explicit statement of the objective(s) or question(s) the review addresses.	Introduction (lines 44-62)
**METHODS**			
Eligibility criteria	5	Specify the inclusion and exclusion criteria for the review and how studies were grouped for the syntheses.	Methods (Data collection: soil organic carbon): lines 228-245
Information sources	6	Specify all databases, registers, websites, organisations, reference lists and other sources searched or consulted to identify studies. Specify the date when each source was last searched or consulted.	Methods (Data collection: soil organic carbon): lines 223-228
Search strategy	7	Present the full search strategies for all databases, registers and websites, including any filters and limits used.	Idem
Selection process	8	Specify the methods used to decide whether a study met the inclusion criteria of the review, including how many reviewers screened each record and each report retrieved, whether they worked independently, and if applicable, details of automation tools used in the process.	Idem
Data collection process	9	Specify the methods used to collect data from reports, including how many reviewers collected data from each report, whether they worked independently, any processes for obtaining or confirming data from study investigators, and if applicable, details of automation tools used in the process.	Methods (Data collection: soil organic carbon): lines 245-251
Data items	10a	List and define all outcomes for which data were sought. Specify whether all results that were compatible with each outcome domain in each study were sought (e.g. for all measures, time points, analyses), and if not, the methods used to decide which results to collect.	All details in Methods (Data collection: soil organic carbon; Data collection: auxiliary data; Data collection: plant functional traits; Dataset compilation: phylogenetic distance)
	10b	List and define all other variables for which data were sought (e.g. participant and intervention characteristics, funding sources). Describe any assumptions made about any missing or unclear information.	Idem
Study risk of bias assessment	11	Specify the methods used to assess risk of bias in the included studies, including details of the tool(s) used, how many reviewers assessed each study and whether they worked independently, and if applicable, details of automation tools used in the process.	Data were extracted by both authors, working together. All studies and data were collegially evaluated to avoid inclusion/exclusion bias. See Methods
Effect measures	12	Specify for each outcome the effect measure(s) (e.g. risk ratio, mean difference) used in the synthesis or presentation of results.	Methods (Data handling and normalisation; Data analysis)
Synthesis methods	13a	Describe the processes used to decide which studies were eligible for each synthesis (e.g. tabulating the study intervention characteristics and comparing against the planned groups for each synthesis (item #5)).	Methods (Data collection: soil organic carbon): lines 234-245
	13b	Describe any methods required to prepare the data for presentation or synthesis, such as handling of missing summary	Methods (Data handling)

Section and Topic	Item #	Checklist item	Location where item is reported
		statistics, or data conversions.	
	13c	Describe any methods used to tabulate or visually display results of individual studies and syntheses.	Not applicable
	13d	Describe any methods used to synthesize results and provide a rationale for the choice(s). If meta-analysis was performed, describe the model(s), method(s) to identify the presence and extent of statistical heterogeneity, and software package(s) used.	Data were normalised to enable comparisons and synthesis of results (see Data handling and normalisation)
	13e	Describe any methods used to explore possible causes of heterogeneity among study results (e.g. subgroup analysis, meta-regression).	Heterogeneity was assessed by using climate, soil properties, past land-use, plant functional types, and stand biomass as factors (see for instance Figures 3-4)
	13f	Describe any sensitivity analyses conducted to assess robustness of the synthesized results.	Data were analysed using different methods, whose results were found to be consistent to each other (Methods: Data analysis)
Reporting bias assessment	14	Describe any methods used to assess risk of bias due to missing results in a synthesis (arising from reporting biases).	Data were evaluated collegially evaluated to avoid inclusion/exclusion bias. For regions where data were scarce, we applied the selection criteria with flexibility to avoid having areas of the world severely under-represented in the dataset.
Certainty assessment	15	Describe any methods used to assess certainty (or confidence) in the body of evidence for an outcome.	Methods: Data analysis
**RESULTS**			
Study selection	16a	Describe the results of the search and selection process, from the number of records identified in the search to the number of studies included in the review, ideally using a flow diagram.	Figure S15 in Supplementary Information
	16b	Cite studies that might appear to meet the inclusion criteria, but which were excluded, and explain why they were excluded.	Examples and criteria are given lines 236-241 (Data collection: soil organic carbon)
Study characteristics	17	Cite each included study and present its characteristics.	Supplementary References 1-3; Source Data
Risk of bias in studies	18	Present assessments of risk of bias for each included study.	Each included study received a score of confidence based on explicit criteria (Data handling and normalisation). This score was used to give to each study a statistical weight proportional to its robustness during data analyses
Results of individual studies	19	For all outcomes, present, for each study: (a) summary statistics for each group (where appropriate) and (b) an effect estimate and its precision (e.g. confidence/credible interval), ideally using structured tables or plots.	Not done because of the high number of original studies
Results of syntheses	20a	For each synthesis, briefly summarise the characteristics and risk of bias among contributing studies.	Not done because of the high number of original studies
	20b	Present results of all statistical syntheses conducted. If meta-analysis was done, present for each the summary estimate and its precision (e.g. confidence/credible interval) and measures of statistical heterogeneity. If comparing groups, describe the direction of the effect.	Results and Supplementary Information
	20c	Present results of all investigations of possible causes of heterogeneity among study results.	Results and Supplementary Information

Section and Topic	Item #	Checklist item	Location where item is reported
	20d	Present results of all sensitivity analyses conducted to assess the robustness of the synthesized results.	Not done
Reporting biases	21	Present assessments of risk of bias due to missing results for each synthesis assessed.	Not done
Certainty of evidence	22	Present assessments of certainty (or confidence) in the body of evidence for each outcome assessed.	Main findings were analysed using a modelling approach, with a quantification of the level of explained variance (Results, Methods, Figure 4)
**DISCUSSION**			
Discussion	23a	Provide a general interpretation of the results in the context of other evidence.	Discussion
	23b	Discuss any limitations of the evidence included in the review.	Not done
	23c	Discuss any limitations of the review processes used.	Not done
	23d	Discuss implications of the results for practice, policy, and future research.	Final paragraph of the Discussion
**OTHER INFORMATION**			
Registration and protocol	24a	Provide registration information for the review, including register name and registration number, or state that the review was not registered.	Not done
	24b	Indicate where the review protocol can be accessed, or state that a protocol was not prepared.	A protocol was initially prepared by the two authors to enable the collaborative work. This protocol was revised, when necessary, during the course of the systematic review. However, the protocol was neither register nor published
	24c	Describe and explain any amendments to information provided at registration or in the protocol.	Amendments were made when a case study presented a characteristic that was not yet taken into account in the protocol
Support	25	Describe sources of financial or non-financial support for the review, and the role of the funders or sponsors in the review.	Described in the section Acknowledgements
Competing interests	26	Declare any competing interests of review authors.	Described in the section Competing interests
Availability of data, code and other materials	27	Report which of the following are publicly available and where they can be found: template data collection forms; data extracted from included studies; data used for all analyses; analytic code; any other materials used in the review.	Described in the dedicated sections (Data availability; Code availability)

From: Page MJ, McKenzie JE, Bossuyt PM, Boutron I, Hoffmann TC, Mulrow CD, et al. The PRISMA 2020 statement: an updated guideline for reporting systematic reviews. BMJ 2021;372:n71. doi: 10.1136/bmj.n71; For more information: http://www.prisma-statement.org/

Supplementary Table S7 | Pedotransfer functions tested to estimate missing values of soil bulk density.

Four pedo-transfer functions were tested:

1. **Function A** [Augusto *et al.* Is 'grey literature' a reliable source of data to characterize soils at the scale of a region? *Eur. J. Soil Sci.* 61, 807–822 (2010)]: BD = soil bulk density (kg L^{-1}); SOC = soil organic carbon (mg g^{-1}); α, β, and γ = fitted parameters.
2. **Function D** [De Vos *et al.* Predictive quality of pedotransfer functions for estimating bulk density of forest soils. *Soil Sci. Soc. Am. J.* 69, 500–510 (2005)]: BD = soil bulk density (kg L^{-1}); LOI = lost on ignition (%); Sand and Clay = textural fractions (%); Depth = position in the soil profile (cm); α, β1, β2, β3, and β4 = fitted parameters. We tested the "CA" model of this publication.
3. **Function F** [Federer *et al.* The organic fraction - bulk density relationship and the expression of nutrient content in forest soils. *Can. J. For. Res.* 23, 1026–1032 (1993)]: BD = soil bulk density (kg L^{-1}); FO = fraction of organic matter [0-1; in mass]; α and β = fitted parameters.
4. **Function RK** [Ruehlmann & Korschens. Calculating the effect of soil organic matter concentration on soil bulk density. *Soil Sci. Soc. Am. J.* 73, 876–885 (2009)]: BD = soil bulk density (kg L^{-1}); SOC = soil organic carbon (mg g^{-1}); α, β, and γ = fitted parameters.

The validation dataset used to test the reliability of the functions was a compilation of measured values (soil bulk density, soil texture, soil organic carbon or soil organic matter, soil depth). This dataset consisted in 208 soils that are representative at the global scale of natural ecosystems (source: Augusto *et al.* Soil parent material - A major driver of plant nutrient limitations in terrestrial ecosystems. *Global Change Biology* 23, 3808–3824 (2017)). Estimated values were plotted versus measured values, and a linear regression was fitted (slope values, and adjusted r^2 values, are presented). Following the recommendation of Pineiro *et al.*, the linear regressions were of the form: $y = f(\hat{y})$, where y and ŷ are the measured value and the estimated value, respectively.

[Pineiro *et al.* How to evaluate models: Observed vs. predicted or predicted vs. observed? *Ecol. Model.* 216, 316–322 (2008)]

Pedotransfer function	Model	Reliability
A	$BD = \alpha + (\beta \times \exp^{(-\gamma \times SOC)})$	slope=0.94, r^2=0.66
D	$BD = (\alpha + \beta1 \times LOI + \beta2 \times Sand + \beta3 \times Clay + \beta4 \times Depth)^{-1}$	slope=0.95, r^2=0.64
F	$BD = (\alpha \times \beta) / ((FO \times \alpha) + ((1\text{-}FO) \times \beta))$	slope=1.12, r^2=0.57
RK	$BD = (\alpha - (\beta \times \gamma)) \times \exp^{(-\gamma \times SOC)}$	slope=0.90, r^2=0.68

Supplementary Table S8 | Comparisons between observed values and global values describing the site properties. When the descriptors of the sites were not provided in the original values (below referred to as *measured values*), we filled the data gaps with simulated values at the global scale in dedicated modelling studies (referred to as *estimated values*; for more details, see subsection *Data collection: auxiliary data* in Methods).

To test the reliability of the external datasets containing the estimated values, we compared them to our measured values (in the sites where the latter were available) by fitting linear regressions. Following the recommendation of Pineiro *et al.*, the linear regressions were of the form: $y = f(\hat{y})$, where y and ŷ are the measured value and the estimated value, respectively. Are presented only the variables for which estimated values were found as reliable enough: MAT = mean annual temperature (°C); MAP = mean annual precipitation (mm yr^{-1}); Elevation (m above sea level); soil texture (content in clay, content in sand, in mg g^{-1}); base saturation of the cation exchange capacity of the soil (%).

[Pineiro *et al.* How to evaluate models: Observed vs. predicted or predicted vs. observed? *Ecol. Model.* 216, 316–322 (2008)]

Variable tested	Results of the linear regression between measured values and estimated values			
MAT (°C)	n = 79	$P < 0.001$	slope = 1.02	$r^2 = 0.99$
MAP (mm/yr)	n = 110	$P < 0.001$	slope = 0.98	$r^2 = 0.90$
Elevation (m asl)	n = 91	$P < 0.001$	slope = 1.03	$r^2 = 0.95$
Soil clay (mg g^{-1})	n = 96	$P < 0.001$	slope = 0.98	$r^2 = 0.71$
Soil sand (mg g^{-1})	n = 91	$P < 0.001$	slope = 1.12	$r^2 = 0.86$
Soil pH	n = 102	$P < 0.001$	slope = 0.88	$r^2 = 0.98$
Soil BS (%)	n = 30	$P < 0.001$	slope = 0.75	$r^2 = 0.82$

Supplementary Table S9 | Regressions used for the gap filling procedure of the plant functional traits. Leaf A_{max}: leaf photosynthetic maximum capacity (µmol g^{-1} s^{-1}); Vc_{max}: leaf photosynthesis carboxylation capacity (µmol/g/s); Stomatal gs: leaf stomatal conductance (mmol m^{-2} s^{-1}); C, N, P, and Ca: content in carbon, nitrogen, phosphorus, or calcium (mg g^{-1}). The proportion of estimated values presents the number of tree species for which the trait value is estimated. Because the tree species were not equally present in the SOC database, and because the tree species that had estimated trait values differed from trait to trait, the percentage value was not proportional to the number of tree species involved in the gap filling procedure. Linear regressions were fitted.

Estimated trait	Predictor	Linear model	Regression performance			Proportion of estimated values	
Leaf A_{max}	Vcmax	$y = 0.187x + 0.003$	$P < 0.001$	$r^2 = 0.49$	n = 34	n = 4	1.5%
Leaf A_{max}	Stomatal gs	$y = 0.00032x + 0.034$	$P < 0.001$	$r^2 = 0.44$	n = 43	n = 2	
Leaf C	Litterfall C	$y = 0.978x$	$P < 0.001$	$r^2 = 0.99$	n = 43	n = 17	13.4%
Leaf C	Fine root C	$y = 1.027x$	$P < 0.001$	$r^2 = 0.99$	n = 54	n = 13	
Leaf N	Litterfall N	$y = 1.433x$	$P < 0.001$	$r^2 = 0.86$	n = 68	n = 6	2.6%
Leaf N	Fine root N	$y = 1.436x$	$P < 0.001$	$r^2 = 0.84$	n = 87	n = 4	
Leaf P	Litterfall P	$y = 1.582x$	$P < 0.001$	$r^2 = 0.87$	n = 49	n = 7	3.1%
Leaf P	Fine root P	$y = 1.412x$	$P < 0.001$	$r^2 = 0.83$	n = 47	n = 4	
Leaf Ca	Litterfall Ca	$y = 0.716x$	$P < 0.001$	$r^2 = 0.78$	n = 34	n = 25	13.7%
Leaf Ca	Fine root Ca	$y = 1.386x$	$P < 0.001$	$r^2 = 0.77$	n = 40	n = 6	

Supplementary Table S10 | Data availability for the main functional traits used in the study. Leaf A_{max}: leaf photosynthetic maximum capacity (µmol g^{-1} s^{-1}); LDMC: leaf dry matter content (g g^{-1}); SLA: specific leaf area (mm^2 mg^{-1}); SRL: specific root length (m g^{-1}); Wood density (kg L^{-1}); Leaf C, lignin, N, P, and Ca: content in carbon, lignin, nitrogen, phosphorus, or calcium (mg g^{-1}); Seed mass (mg $seed^{-1}$); Tree max height: maximum height observed for the tree species (m). The number of values from TRY, or other sources, represents the total data availability before applying the procedures of curation, homogenisation, and averaging (see Methods); After having calculating the mean values per each tree species, we calculated the proportion of the tree species having a non-missing measured value, and the proportion of tree species having an estimated value.

Studied traits	Values from TRY	Values from other sources	Proportion of non-missing values	Proportion of estimated values	Range of values
Leaf Amax	1 000	1 964	50%	1.5%	0.012–0.583
LDMC	8 734	3 905	36%	0.0%	0.25–0.55
SLA	2 790	9 925	75%	0.0%	1.2–97.0
SRL	186	251	37%	0.0%	1.9–142.4
Wood density	1 149	863	99%	0.0%	0.28–0.91
Leaf C	2 295	63	100%	13.4%	272–589
Leaf lignin	72	408	65%	0.0%	38–455
Leaf N	5 339	1 855	76%	2.6%	6.5–54.4
Leaf P	1 422	647	64%	3.1%	0.35–3.31
Leaf Ca	312	91	51%	13.7%	1.4–23.1
Seed mass	2 524	2 647	92%	0.0%	0.1–17696
Tree max height	24 919	387	96%	0.0%	2–120

Supplementary Table S11 | Fictive example of the values available in supplementary information and the values used during data analysis to test a possible relationship between two variables. SLA: specific leaf area; RR: relative value (see above); SOC: soil organic carbon; SI: supplementary information containing the final dataset (Appendix 1); NA: non-available value. In this example, RR.SLA was not presented in the final dataset for the site B because one tree species had a missing-value, disabling any relevant comparison with the RR.SOC (calculated based on all tree species).

Site	Species	SLA (mm^2 mg)	RR.SLA (in SI)	RR.SLA (recalculated)	SOC (kg m^2)	RR.SOC (in SI)	RR.SOC (recalculated)
A	*Abies a.*	5.6	-0.62	-0.62	8.5	0.00	0.00
A	*Fagus s.*	18.2	0.56	0.56	7.9	-0.07	-0.07
A	*Picea a.*	7.4	-0.34	-0.34	9.1	0.07	0.07
B	*Acer p.*	20.1	NA	0.57	4.4	-0.11	-0.10
B	*Cupressus m.*	NA	NA	NA	5.1	0.03	NA
B	*Picea a.*	7.4	NA	-0.43	5.3	0.07	0.09
B	*Pinus p.*	6.5	NA	-0.56	4.9	-0.01	0.01

For example, in the site B, the absolute SOC values are 4.4, 5.1, 5.3, and 4.1 for the stands *Acer*, *Cupressus*, *Picea*, and *Pinus*, respectively. The "site value" is calculated as the mean values of its stands:

$$SOC.Site = mean(SOC.Acer, SOC.Cupressus, SOC.Picea, SOC.Pinus) = \frac{4.4 + 5.1 + 5.3 + 4.1}{4} = 4.925$$

Then, the relative values of SOC are calculated as the ratio of the stand value per the site value, with a log function to reduce the statistical weight of outliers. For the Acer stand:

$$RR.Acer = log\left(\frac{SOC.Acer}{SOC.Site}\right) = log\left(\frac{4.4}{4.925}\right) = -0.113$$

The negative relative ratio value for the *Acer* stand indicates that the SOC pool was lower than the mean SOC pool of the site.

In our study, we sometimes calculated the effect of plant functional types (*e.g.* angiosperms *versus* gymnosperms, or arbuscular mycorrhizal tree species *versus* ectomycorrhizal tree species). Still using the B site as an example, the effect of the spermatophyte type was calculated as follows:

$$RR = log\left(\frac{SOC.angiosperms}{SOC.gymnosperms}\right) = log\left(\frac{mean\{SOC.Acer\}}{mean\{SOC.Cupressus, SOC.Picea, SOC.Pinus\}}\right)$$

which is

$$RR = log\left(\frac{4.4}{mean\{5.1, 5.3, 4.9\}}\right) = log\left(\frac{4.4}{5.1}\right) = -0.148$$

The negative relative ratio value indicates that the SOC pool under the angiosperm species was on average lower than under the gymnosperm species.

Supplementary References

Supplementary Reference 1 – List of references used to build the Soil Organic Carbon dataset

1 Abiyu, A., Lemenih, M., Gratzer, G., Aerts, R., Teketay, D., & Glatzel, G. (2011). Status of native woody species diversity and soil characteristics in an exclosure and in plantations of Eucalyptus globulus and Cupressus lusitanica in Northern Ethiopia. Mountain Research and Development, 31(2), 144-152.

2 Alban, D. H., Perala, D. A., & Schlaegel, B. E. (1978). Biomass and nutrient distribution in aspen, pine, and spruce stands on the same soil type in Minnesota. Canadian Journal of Forest Research, 8(3), 290-299.

3 Alriksson, A., & Eriksson, H. M. (1998). Variations in mineral nutrient and C distribution in the soil and vegetation compartments of five temperate tree species in NE Sweden. Forest Ecology and Management, 108(3), 261-273.

4 Andivia, E., Rolo, V., Jonard, M., Formánek, P., & Ponette, Q. (2016). Tree species identity mediates mechanisms of top soil carbon sequestration in a Norway spruce and European beech mixed forest. Annals of forest science, 73(2), 437-447.

5 Ayres, E., Steltzer, H., Berg, S., Wallenstein, M. D., Simmons, B. L., & Wall, D. H. (2009). Tree species traits influence soil physical, chemical, and biological properties in high elevation forests. Plos One, 4(6), e5964.

6 Bauhus, J., & Pare, D. (1998). Effects of tree species, stand age and soil type on soil microbial biomass and its activity in a southern boreal forest. Soil Biology and Biochemistry, 30(8-9), 1077-1089.

7 Belyaev, A. B. (2007). Long-term dynamics of the properties of leached chernozems under different forest plantations. Eurasian Soil Science, 40(8), 821-829.

8 Berger, T. W., Inselsbacher, E., & Zechmeister-Boltenstern, S. (2010). Carbon dioxide emissions of soils under pure and mixed stands of beech and spruce, affected by decomposing foliage litter mixtures. Soil Biology and Biochemistry, 42(6), 986-997.

9 Boča, A., & Van Miegroet, H. (2017). Can carbon fluxes explain differences in soil organic carbon storage under aspen and conifer forest overstories?. Forests, 8(4), 118.

10 Boerner, R. E., & Koslowsky, S. D. (1989). Microsite variations in soil chemistry and nitrogen mineralization in a beech-maple forest. Soil Biology and Biochemistry, 21(6), 795-801.

11 Bonnevie-Svendsen, C., & Gjems, O. (1957). Amount and chemical composition of the litter from larch, beech, Norway spruce and Scots pine stands and its effect on the soil. Meddelelser fra det norske skogforsøksvesen, 14, 111-174.

12 Borken, W., XU, Y. J., Davidson, E. A., & Beese, F. (2002). Site and temporal variation of soil respiration in European beech, Norway spruce, and Scots pine forests. Global Change Biology, 8(12), 1205-1216.

13 Charro, E., Gallardo, J. F., & Moyano, A. (2010). Degradability of soils under oak and pine in Central Spain. European journal of forest research, 129(1), 83-91.

14 Chavan, K. N., Kenjale, R. Y., & Chavan, A. S. (1995). Effect of forest tree species on properties of lateritic soil. Journal of the Indian Society of Soil Science, 43(1), 43-46.

15 Chen, G. S., Yang, Y. S., Xie, J. S., Guo, J. F., Gao, R., & Qian, W. (2005). Conversion of a natural broad-leafed evergreen forest into pure plantation forests in a subtropical area: effects on carbon storage. Annals of forest science, 62(7), 659-668.

16 Cole, D. W., Compton, J. E., Edmonds, R. L., Homann, P. S., & Van Miegroet, H. (1995). Comparison of carbon accumulation in Douglas fir and red alder forests. Carbon forms and functions in forest soils, 527-546.

17 Compton, J. E., Boone, R. D., Motzkin, G., & Foster, D. R. (1998). Soil carbon and nitrogen in a pine-oak sand plain in central Massachusetts: role of vegetation and land-use history. Oecologia, 116(4), 536-542.

18 Cook, R. L., Binkley, D., Mendes, J. C. T., & Stape, J. L. (2014). Soil carbon stocks and forest biomass following conversion of pasture to broadleaf and conifer plantations in southeastern Brazil. Forest Ecology and Management, 324, 37-45.

19 Cremer, M., & Prietzel, J. (2017). Soil acidity and exchangeable base cation stocks under pure and mixed stands of European beech, Douglas fir and Norway spruce. Plant and Soil, 415(1-2), 393-405.
20 Cross, A., & Perakis, S. S. (2011). Complementary models of tree species–soil relationships in old-growth temperate forests. Ecosystems, 14(2), 248-260.
21 Defrieri, R. L., Sarti, G., Tortarolo, M. F., Escobar-Ortega, J., García de Salamone, I., D'Auria, F., & Effron, D. (2011). Biochemical and microbiological properties of Argentinean Patagonia soil with implanted forest species. Journal of soil science and plant nutrition, 11(3), 111-124.
22 De Marco, A., Esposito, F., Berg, B., Giordano, M., & De Santo, A. V. (2013). Soil C and N sequestration in organic and mineral layers of two coeval forest stands implanted on pyroclastic material (Mount Vesuvius, South Italy). Geoderma, 209, 128-135.
23 Demessie, A., Singh, B. R., & Lal, R. (2011). Soil carbon and nitrogen stocks under plantations in Gambo District, Southern Ethiopia. Journal of Sustainable Forestry, 30(6), 496-517.
24 Deng, H., Zhang, B., Yin, R., Wang, H. L., Mitchell, S. M., Griffiths, B. S., & Daniell, T. J. (2010). Long-term effect of re-vegetation on the microbial community of a severely eroded soil in sub-tropical China. Plant and soil, 328(1-2), 447-458.
25 Devi, B., Bhardwaj, D. R., Panwar, P., Pal, S., Gupta, N. K., & Thakur, C. L. (2012). Carbon allocation, sequestration and carbon dioxide mitigation under plantation forests of north western Himalaya, India. Annals of Forest Research, 56(1), 123-135.
26 Díaz-Pinés, E., Rubio, A., Van Miegroet, H., Montes, F., & Benito, M. (2011). Does tree species composition control soil organic carbon pools in Mediterranean mountain forests?. Forest Ecology and Management, 262(10), 1895-1904.
27 Dijkstra, F. A., & Fitzhugh, R. D. (2003). Aluminum solubility and mobility in relation to organic carbon in surface soils affected by six tree species of the northeastern United States. Geoderma, 114(1-2), 33-47.
28 Fissore, C., Giardina, C. P., Kolka, R. K., Trettin, C. C., King, G. M., Jurgensen, M. F., ... & McDowell, S. D. (2008). Temperature and vegetation effects on soil organic carbon quality along a forested mean annual temperature gradient in North America. Global Change Biology, 14(1), 193-205.
29 Fonseca, F., de Figueiredo, T., Vilela, Â., Santos, R., de Carvalho, A. L., Almeida, E., & Nunes, L. (2019). Impact of tree species replacement on carbon stocks in a Mediterranean mountain area, NE Portugal. Forest Ecology and Management, 439, 181-188.
30 Forrester, D. I., Bauhus, J., & Cowie, A. L. (2005). On the success and failure of mixed-species tree plantations: lessons learned from a model system of Eucalyptus globulus and Acacia mearnsii. Forest Ecology and Management, 209(1-2), 147-155.
31 Goh, K. M., & Heng, S. (1987). The quantity and nature of the forest floor and topsoil under some indigenous forests and nearby areas converted to Pinus radiata plantations in South Island New Zealand. New Zealand Journal of Botany, 25(2), 243-254.
32 Gomes da Silva, L., MENDES, I. D. C., Reis Junior, F. B., Fernandes, M. F., de Melo, J. T., & Kato, E. (2009). Atributo físicos, químicos e biológicos de um Latossolo de Cerrado sob plantio de espécies florestais. Embrapa Tabuleiros Costeiros-Boletim de Pesquisa e Desenvolvimento (INFOTECA-E).
33 Haghdoost, N., Akbarinia, M., Hosseini, S. M., & Kooch, Y. (2011). Conversion of Hyrcanian degraded forests to plantations: Effects on soil C and N stocks. Ann. Biol. Res, 2, 385-399.
34 Hansson, K., Olsson, B. A., Olsson, M., Johansson, U., & Kleja, D. B. (2011). Differences in soil properties in adjacent stands of Scots pine, Norway spruce and silver birch in SW Sweden. Forest Ecology and Management, 262(3), 522-530.
35 Hoogmoed, M., Cunningham, S. C., Baker, P., Beringer, J., & Cavagnaro, T. R. (2014). N-fixing trees in restoration plantings: effects on nitrogen supply and soil microbial communities. Soil Biology and Biochemistry, 77, 203-212.
36 Huang, Z., Davis, M. R., Condron, L. M., & Clinton, P. W. (2011). Soil carbon pools, plant biomarkers and mean carbon residence time after afforestation of grassland with three tree species. Soil Biology and Biochemistry, 43(6), 1341-1349.
37 Jiang, P. K., & Qiu-Fang, X. U. (2006). Abundance and dynamics of soil labile carbon pools under different types of forest vegetation. Pedosphere, 16(4), 505-511.

38 Jiang, Y. M., Chen, C. R., Liu, Y. Q., & Xu, Z. H. (2010). Soil soluble organic carbon and nitrogen pools under mono-and mixed species forest ecosystems in subtropical China. Journal of Soils and Sediments, 10(6), 1071-1081.
39 Johnsen, K. H., Samuelson, L. J., Sanchez, F. G., & Eaton, R. J. (2013). Soil carbon and nitrogen content and stabilization in mid-rotation, intensively managed sweetgum and loblolly pine stands. Forest ecology and management, 302, 144-153.
40 Kasel, S., Singh, S., Sanders, G. J., & Bennett, L. T. (2011). Species-specific effects of native trees on soil organic carbon in biodiverse plantings across north-central Victoria, Australia. Geoderma, 161(1-2), 95-106.
41 Kaye, J. P., Resh, S. C., Kaye, M. W., & Chimner, R. A. (2000). Nutrient and carbon dynamics in a replacement series of Eucalyptus and Albizia trees. Ecology, 81(12), 3267-3273.
42 King, J. A., & Campbell, B. M. (1994). Soil organic matter relations in five land cover types in the miombo region (Zimbabwe). Forest Ecology and management, 67(1-3), 225-239.
43 Kooch, Y., Hosseini, S. M., Zaccone, C., Jalilvand, H., & Hojjati, S. M. (2012). Soil organic carbon sequestration as affected by afforestation: the Darab Kola forest (North of Iran) case study. Journal of Environmental Monitoring, 14(9), 2438-2446.
44 Kostenko, I. V. (2018). The impact of artificial forest plantations on mountain-meadow soils of Crimea. Eurasian Soil Science, 51(5), 485-494.
45 Kulakova, N. (2012). Impact of plant species on the formation of carbon and nitrogen stock in soils under semi-desert conditions. European Journal of Forest Research, 131(6), 1717-1726.
46 Ladegaard-Pedersen, P., Elberling, B., & Vesterdal, L. (2005). Soil carbon stocks, mineralization rates, and CO2 effluxes under 10 tree species on contrasting soil types. Canadian Journal of Forest Research, 35(6), 1277-1284.
47 Laganière, J., Angers, D. A., Paré, D., Bergeron, Y., & Chen, H. Y. (2011). Black spruce soils accumulate more uncomplexed organic matter than aspen soils. Soil Science Society of America Journal, 75(3), 1125-1132.
48 Laganiere, J., Paré, D., Bergeron, Y., Chen, H. Y., Brassard, B. W., & Cavard, X. (2013). Stability of soil carbon stocks varies with forest composition in the Canadian boreal biome. Ecosystems, 16(5), 852-865.
49 Laik, R., Kumar, K., Das, D. K., & Chaturvedi, O. P. (2009). Labile soil organic matter pools in a calciorthent after 18 years of afforestation by different plantations. Applied Soil Ecology, 42(2), 71-78.
50 Lakshmanan, C. (1962). Chemical and morphological characteristics of soils as influenced by several tree species (Doctoral dissertation, The Ohio State University).
51 Landgraf, D., Wedig, S., & Klose, S. (2005). Medium-and short-term available organic matter, microbial biomass, and enzyme activities in soils under Pinus sylvestris L. and Robinia pseudoacacia L. in a sandy soil in NE Saxony, Germany. Journal of Plant Nutrition and Soil Science, 168(2), 193-201.
52 Langenbruch, C., Helfrich, M., & Flessa, H. (2012). Effects of beech (Fagus sylvatica), ash (Fraxinus excelsior) and lime (Tilia spec.) on soil chemical properties in a mixed deciduous forest. Plant and Soil, 352(1-2), 389-403.
53 Laudicina, V. A., De Pasquale, C., Conte, P., Badalucco, L., Alonzo, G., & Palazzolo, E. (2012). Effects of afforestation with four unmixed plant species on the soil–water interactions in a semiarid Mediterranean region (Sicily, Italy). Journal of Soils and Sediments, 12(8), 1222-1230.
54 Lee, S. K., Son, Y., Noh, N. J., Heo, S. J., Yoon, T. K., Lee, A. R., ... & Lee, W. K. (2009). Carbon storage of natural pine and oak pure and mixed forests in Hoengseong, Kangwon. Journal of Korean Society of Forest Science, 98(6), 772-779.
55 Lejon, D. P., Chaussod, R., Ranger, J., & Ranjard, L. (2005). Microbial community structure and density under different tree species in an acid forest soil (Morvan, France). Microbial Ecology, 50(4), 614-625.
56 Lemenih, M., Olsson, M., & Karltun, E. (2004). Comparison of soil attributes under Cupressus lusitanica and Eucalyptus saligna established on abandoned farmlands with continuously cropped farmlands and natural forest in Ethiopia. Forest ecology and management, 195(1-2), 57-67.

57 Lemma, B., Kleja, D. B., Nilsson, I., & Olsson, M. (2006). Soil carbon sequestration under different exotic tree species in the southwestern highlands of Ethiopia. Geoderma, 136(3-4), 886-898.
58 Lemma, B. (2012). Soil chemical properties and nutritional status of trees in pure and mixed-species stands in south Ethiopia. Journal of Plant Nutrition and Soil Science, 175(5), 769-774.
59 Liang, C., Fujinuma, R., Wei, L., & Balser, T. C. (2007). Tree species-specific effects on soil microbial residues in an upper Michigan old-growth forest system. Forestry, 80(1), 65-72.
60 Lisanework, N., & Michelsen, A. (1994). Litterfall and nutrient release by decomposition in three plantations compared with a natural forest in the Ethiopian highland. Forest Ecology and Management, 65(2-3), 149-164.
61 López-Marcos, D., Martínez-Ruiz, C., Turrión, M. B., Jonard, M., Titeux, H., Ponette, Q., & Bravo, F. (2018). Soil carbon stocks and exchangeable cations in monospecific and mixed pine forests. European Journal of Forest Research, 137(6), 831-847.
62 Lu, S., Chen, C., Zhou, X., Xu, Z., Bacon, G., Rui, Y., & Guo, X. (2012). Responses of soil dissolved organic matter to long-term plantations of three coniferous tree species. Geoderma, 170, 136-143.
63 Lugo, A. E., Cuevas, E., & Sanchez, M. J. (1990). Nutrients and mass in litter and top soil of ten tropical tree plantations. Plant and Soil, 125(2), 263-280.
64 Manzanares, P. N., & Navarro-Cerrillo, R. M. (2004). Efecto de la sustitución del bosque nativo por plantaciones de pino y eucalipto sobre el horizonte orgánico en concepción (Chile). Revista ITEA, 100(2), 118-131.
65 Matos, E. S., Freese, D., Ślązak, A., Bachmann, U., Veste, M., & Hüttl, R. F. (2010). Organic-carbon and nitrogen stocks and organic-carbon fractions in soil under mixed pine and oak forest stands of different ages in NE Germany. Journal of Plant Nutrition and Soil Science, 173(5), 654-661.
66 McFarland, J. W., Ruess, R. W., Kielland, K., Pregitzer, K., & Hendrick, R. (2010). Glycine mineralization in situ closely correlates with soil carbon availability across six North American forest ecosystems. Biogeochemistry, 99(1-3), 175-191.
67 Mellor, N. J., Hellerich, J., Drijber, R., Morris, S. J., Stromberger, M. E., & Paul, E. A. (2013). Changes in ecosystem carbon following afforestation of native sand prairie. Soil Science Society of America Journal, 77(5), 1613-1624.
68 Melvin, A. M., & Goodale, C. L. (2013). Tree species and earthworm effects on soil nutrient distribution and turnover in a northeastern United States common garden. Canadian Journal of Forest Research, 43(2), 180-187.
69 Menyailo, O. V., & Hungate, B. A. (2005). Tree species effects on potential production and consumption of carbon dioxide, methane, and nitrous oxide: the Siberian afforestation experiment. In Tree Species Effects on Soils: Implications for Global Change (pp. 293-305). Springer, Dordrecht.
70 Mueller, K. E., Eissenstat, D. M., Hobbie, S. E., Oleksyn, J., Jagodzinski, A. M., Reich, P. B., ... & Chorover, J. (2012). Tree species effects on coupled cycles of carbon, nitrogen, and acidity in mineral soils at a common garden experiment. Biogeochemistry, 111(1-3), 601-614.
71 Nadelhoffer, K. J., Aber, J. D., & Melillo, J. M. (1984). Seasonal patterns of ammonium and nitrate uptake in nine temperate forest ecosystems. Plant and Soil, 80(3), 321-335.
72 Neirynck, J., Mirtcheva, S., Sioen, G., & Lust, N. (2000). Impact of Tilia platyphyllos Scop., Fraxinus excelsior L., Acer pseudoplatanus L., Quercus robur L. and Fagus sylvatica L. on earthworm biomass and physico-chemical properties. Forest Ecology and Management, 133(3), 275-286.
73 Nihlgård, B. (1971). Pedological influence of spruce planted on former beech forest soils in Scania, South Sweden. Oikos, 302-314.
74 Noble, A. D., Berthelsen, S., & Mather, J. (2005). Changes in soil chemical properties under two contrasting plantation systems on the Zululand coastal plain, South Africa. FAO report "Management of tropical sandy soils for sustainable agriculture. A holistic approach for sustainable".

75 Noh, N. J., Chung, H., Ryu, S. R., Son, Y., Lee, S. K., Yoon, T. K., ... & Kim, J. (2012). Changes in soil properties of Abies holophylla and Quercus-dominated stands 4 years after trenching. Scandinavian Journal of Forest Research, 27(6), 597-604.
76 Nsabimana, D., Klemedtson, L., Kaplin, B. A., & Wallin, G. (2009). Soil CO2 flux in six monospecific forest plantations in Southern Rwanda. Soil Biology and Biochemistry, 41(2), 396-402.
77 Olsson, B. A., Hansson, K., Persson, T., Beuker, E., & Helmisaari, H. S. (2012). Heterotrophic respiration and nitrogen mineralisation in soils of Norway spruce, Scots pine and silver birch stands in contrasting climates. Forest Ecology and Management, 269, 197-205.
78 Omoro, L., Starr, M., & Pellikka, P. K. (2013). Tree biomass and soil carbon stocks in indigenous forests in comparison to plantations of exotic species in the Taita Hills of Kenya. Silva Fennica, 47(2), 1-18.
79 Oostra, S., Majdi, H., & Olsson, M. (2006). Impact of tree species on soil carbon stocks and soil acidity in southern Sweden. Scandinavian Journal of Forest Research, 21(5), 364-371.
80 Oulehle, F., Růžek, M., Tahovská, K., Bárta, J., & Myška, O. (2016). Carbon and nitrogen pools and fluxes in adjacent mature Norway spruce and European beech forests. Forests, 7(11), 282.
81 Ovington, J. D. (1956). Studies of the development of woodland conditions under different trees: IV. The ignition loss, water, carbon and nitrogen content of the mineral soil. Journal of Ecology, 44(1), 171-179.
82 Paul, E. A., Morris, S. J., Six, J., Paustian, K., & Gregorich, E. G. (2003). Interpretation of soil carbon and nitrogen dynamics in agricultural and afforested soils. Soil Science Society of America Journal, 67(5), 1620-1628.
83 Peng, S., Chen, A., Fang, H., Wu, J., & Liu, G. (2013). Effects of vegetation restoration types on soil quality in Yuanmou dry-hot valley, China. Soil Science and Plant Nutrition, 59(3), 347-360.
84 Quideau, S. A., Graham, R. C., Chadwick, O. A., & Wood, H. B. (1998). Organic carbon sequestration under chaparral and pine after four decades of soil development. Geoderma, 83(3-4), 227-242.
85 Ramesh, T., Manjaiah, K. M., Tomar, J. M. S., & Ngachan, S. V. (2013). Effect of multipurpose tree species on soil fertility and CO2 efflux under hilly ecosystems of Northeast India. Agroforestry systems, 87(6), 1377-1388.
86 Rastvorova, O. G. (1970). Effect of various stands on the forest-growth properties of grey forest soils. Dolk. Otd. i Komis. Georg. Obsc. SSSR, 1970(13), 31-45.
87 Riestra, D., Noellemeyer, E., & Quiroga, A. (2012). Soil texture and forest species condition the effect of afforestation on soil quality parameters. Soil science, 177(4), 279-287.
88 Russell, A. E., Raich, J. W., Valverde-Barrantes, O. J., & Fisher, R. F. (2007). Tree species effects on soil properties in experimental plantations in tropical moist forest. Soil Science Society of America Journal, 71(4), 1389-1397.
89 Schulp, C. J., Nabuurs, G. J., Verburg, P. H., & de Waal, R. W. (2008). Effect of tree species on carbon stocks in forest floor and mineral soil and implications for soil carbon inventories. Forest ecology and management, 256(3), 482-490.
90 Scott, D. A., & Messina, M. G. (2010). Soil properties in 35 y old pine and hardwood plantations after conversion from mixed pine-hardwood forest. The American Midland Naturalist, 163(1), 197-211.
91 Sevgi, O., Makineci, E., & Karaoz, O. (2011). The forest floor and mineral soil carbon pools of six different forest tree species. Ekoloji, 20(81), 8-14.
92 Singh, B., Tripathi, K. P., Jain, R. K., & Behl, H. M. (2000). Fine root biomass and tree species effects on potential N mineralization in afforested sodic soils. Plant and soil, 219(1-2), 81-89.
93 Smith, C. K., Gholz, H. L., & de Assis Oliveira, F. (1998). Soil nitrogen dynamics and plant-induced soil changes under plantations and primary forest in lowland Amazonia, Brazil. Plant and Soil, 200(2), 193-204.
94 Shukla, M. K., Lal, R., Ebinger, M., & Meyer, C. (2006). Physical and chemical properties of soils under some piñon-juniper-oak canopies in a semi-arid ecosystem in New Mexico. Journal of arid environments, 66(4), 673-685.
95 Sigurðardóttir, R. (2000). Effects of different forest types on total ecosystem carbon sequestration in Hallormsstaður forest, eastern Iceland. Yale Univ., New Haven, CT.

96 Son, Y., & Gower, S. T. (1992). Nitrogen and phosphorus distribution for five plantation species in southwestern Wisconsin. Forest Ecology and Management, 53(1-4), 175-193.
97 Susyan, E. A., Ananyeva, N. D., Gavrilenko, E. G., Chernova, O. V., & Bobrovskii, M. V. (2009). Microbial biomass carbon in the profiles of forest soils of the southern taiga zone. Eurasian Soil Science, 42(10), 1148-1155.
98 Tang, G., & Li, K. (2013). Tree species controls on soil carbon sequestration and carbon stability following 20 years of afforestation in a valley-type savanna. Forest Ecology and Management, 291, 13-19.
99 Thapa, N., Upadhaya, K., Baishya, R., & Barik, S. K. (2011). Effect of plantation on plant diversity and soil status of tropical forest ecosystems in Meghalaya, northeast India. International Journal of Ecology and Environmental Sciences, 37(1), 61-73.
100 Turner, J., & Kelly, J. (1977). Soil chemical properties under naturally regenerated Eucalyptus spp and planted Douglas-fir. Australian Forest Research, 7, 163-172.
101 Turner, J., & Lambert, M. J. (1988). Soil properties as affected by Pinus radiata plantations. New Zealand Journal of Forestry Science, 18(1), 77-91.
102 Turner, D. P., & Franz, E. H. (1985). The influence of western hemlock and western redcedar on microbial numbers, nitrogen mineralization, and nitrification. Plant and Soil, 88(2), 259-267.
103 Turner, D. P., Sollins, P., Leuking, M., & Rudd, N. (1993). Availability and uptake of inorganic nitrogen in a mixed old-growth coniferous forest. Plant and Soil, 148(2), 163-174.
104 Vesterdal, L., Schmidt, I. K., Callesen, I., Nilsson, L. O., & Gundersen, P. (2008). Carbon and nitrogen in forest floor and mineral soil under six common European tree species. Forest Ecology and Management, 255(1), 35-48.
105 Wang, F., Li, Z., Xia, H., Zou, B., Li, N., Liu, J., & Zhu, W. (2010). Effects of nitrogen-fixing and non-nitrogen-fixing tree species on soil properties and nitrogen transformation during forest restoration in southern China. Soil Science & Plant Nutrition, 56(2), 297-306.
106 Wang, H., Liu, S. R., Mo, J. M., Wang, J. X., Makeschin, F., & Wolff, M. (2010). Soil organic carbon stock and chemical composition in four plantations of indigenous tree species in subtropical China. Ecological research, 25(6), 1071-1079.
107 Wang, Q., Xiao, F., Zhang, F., & Wang, S. (2013). Labile soil organic carbon and microbial activity in three subtropical plantations. Forestry, 86(5), 569-574.
108 Washburn, C. S., & Arthur, M. A. (2003). Spatial variability in soil nutrient availability in an oak-pine forest: potential effects of tree species. Canadian Journal of Forest Research, 33(12), 2321-2330.
109 Wen, L., Lei, P., Xiang, W., Yan, W., & Liu, S. (2014). Soil microbial biomass carbon and nitrogen in pure and mixed stands of Pinus massoniana and Cinnamomum camphora differing in stand age. Forest Ecology and Management, 328, 150-158.
110 Xu, X., Inubushi, K., & Sakamoto, K. (2006). Effect of vegetations and temperature on microbial biomass carbon and metabolic quotients of temperate volcanic forest soils. Geoderma, 136(1-2), 310-319.
111 Yan, W. D., Xu, W. M., Chen, X. Y., Tian, D. L., Peng, Y. Y., Zhen, W., ... & Xu, J. (2014). Soil CO2 flux in different types of forests under a subtropical microclimatic environment. Pedosphere, 24(2), 243-250.
112 Zhao, Q., Zeng, D. H., & Fan, Z. P. (2010). Nitrogen and phosphorus transformations in the rhizospheres of three tree species in a nutrient-poor sandy soil. Applied soil ecology, 46(3), 341-346.
113 Zheng, H., Ouyang, Z., Xu, W., Wang, X., Miao, H., Li, X., & Tian, Y. (2008). Variation of carbon storage by different reforestation types in the hilly red soil region of southern China. Forest Ecology and Management, 255(3-4), 1113-1121.
114 Zhiyanski, M., Kolev, K., Sokolovska, M., & Hursthouse, A. (2008). Tree species effect on soils in central Stara Planina Mountains. Nauka za Gorata, 45(4), 65-82.

Supplementary Reference 2 – List of references used to complement the Plant Functional Traits dataset

115 Abubacker, M. N., & Prince, M. (2013). Decomposition of lignin and holocellulose of Acacia dealbata Link (Mimosoideae) leaves, twigs and barks by fungal isolates from virgin forest ecosystem of Doddabetta Belt of Nilgiris. Biosciences Biotechnology Research Asia, 10(2), 719-726.

116 Aguilera, A. G., Alpert, P., Dukes, J. S., & Harrington, R. (2010). Impacts of the invasive plant Fallopia japonica (Houtt.) on plant communities and ecosystem processes. Biological Invasions, 12(5), 1243-1252.

117 Alban, D. H. (1982). Effects of nutrient accumulation by aspen, spruce, and pine on soil properties. Soil Science Society of America Journal, 46(4), 853-861.

118 Anthofer, J., Hanson, J., & Jutzi, S. C. (1998). Wheat growth as influenced by application of agroforestry-tree prunings in Ethiopian highlands. Agroforestry Systems, 40(1), 1-18.

119 Ashagrie, Y., & Zech, W. (2010). Dynamics of dissolved nutrients in forest floor leachates: comparison of a natural forest ecosystem with monoculture tree species plantations in south-east Ethiopia. Ecohydrology & Hydrobiology, 10(2-4), 183-190.

120 Atkin, O. K., Schortemeyer, M., McFarlane, N., & Evans, J. R. (1998). Variation in the components of relative growth rate in 10 Acacia species from contrasting environments. Plant, Cell & Environment, 21(10), 1007-1017.

121 Azeez, J. O. (2019). Recycling organic waste in managed tropical forest ecosystems: effects of arboreal litter types on soil chemical properties in Abeokuta, southwestern Nigeria. Journal of Forestry Research, 30(5), 1903-1911.

122 Bargali, K., & Bargali, S. S. (2009). Acacia nilotica: a multipurpose leguminous plant. Nature and Science, 7(4), 11-19.

123 Barnard, H. R., & Ryan, M. G. (2003). A test of the hydraulic limitation hypothesis in fast-growing Eucalyptus saligna. Plant, Cell & Environment, 26(8), 1235-1245.

124 Bell, D. T., & Ward, S. C. (1984). Foliar and twig macronutrients (N, P, K, Ca and Mg) in selected species of Eucalyptus used in rehabilitation: sources of variation. Plant and Soil, 81(3), 363-376.

125 Berg, B., Johansson, M. B., Ekbohm, G., McClaugherty, C., Rutigliano, F., & Santo, A. V. D. (1996). Maximum decomposition limits of forest litter types: a synthesis. Canadian Journal of Botany, 74(5), 659-672.

126 Birhane, E., Desalegn, T., Kebede, F., Giday, K., Hishe, H., & Hadgu, K. M. (2019). In situ leaf litter production, decomposition and nutrient release of dry Afromontane trees. East African Agricultural and Forestry Journal, 83(3), 176-190.

127 Boulmane, M., Oubrahim, H., Halim, M., Bakker, M. R., & Augusto, L. (2017). The potential of Eucalyptus plantations to restore degraded soils in semi-arid Morocco (NW Africa). Annals of Forest Science, 74(3), 57.

128 Buajan, S., Liu, J., He, Z., & Feng, X. (2018). Effect of gap sizes on specific leaf area and chlorophyll contents at the Castanopsis kawakamii natural reserve forest, China. Forests, 9(11), 682.

129 Calvo-Alvarado, J. C., McDowell, N. G., & Waring, R. H. (2008). Allometric relationships predicting foliar biomass and leaf area: sapwood area ratio from tree height in five Costa Rican rain forest species. Tree physiology, 28(11), 1601-1608.

130 Cattanio, J. H., Kuehne, R., & Vlek, P. L. (2008). Organic material decomposition and nutrient dynamics in a mulch system enriched with leguminous trees in the Amazon. Revista Brasileira de Ciência do Solo, 32(3), 1073-1086.

131 Chao, L., Liu, Y., Freschet, G. T., Zhang, W., Yu, X., ... & Wang, S. (2019). Litter carbon and nutrient chemistry control the magnitude of soil priming effect. Functional Ecology, 33(5), 876-888.

132 Chaturvedi, O. P., & Singh, J. S. (1982). Total biomass and biomass production of Pinus roxburghii trees growing in all-aged natural forests. Canadian Journal of Forest Research, 12(3), 632-640.

133 Chaturvedi, R. K., Raghubanshi, A. S., & Singh, J. S. (2011). Leaf attributes and tree growth in a tropical dry forest. Journal of Vegetation Science, 22(5), 917-931.

134 Chaturvedi, R. K., & Raghubanshi, A. S. (2018). Leaf size and specific leaf area of tropical deciduous trees increase with elevation in soil moisture content. Int. Journal of Hydrology, 2(4), 466-469.
135 Choat, B., Jansen, S., Brodribb, T. J., Cochard, H., Delzon, S., Bhaskar, R., ... & Jacobsen, A. L. (2012). Global convergence in the vulnerability of forests to drought. Nature, 491(7426), 752-755.
136 Choi, D. S., Kayama, M., Jin, H. O., Lee, C. H., Izuta, T., & Koike, T. (2006). Growth and photosynthetic responses of two pine species (Pinus koraiensis and Pinus rigida) in a polluted industrial region in Korea. Environmental Pollution, 139(3), 421-432.
137 Cizungu, L., Staelens, J., Huygens, D., Walangululu, J., Muhindo, D., Van Cleemput, O., & Boeckx, P. (2014). Litterfall and leaf litter decomposition in a central African tropical mountain forest and Eucalyptus plantation. Forest Ecology and Management, 326, 109-116.
138 Coker, G. W. R. (2006). Leaf area index in closed canopies: an indicator of site quality. Master report. School of Forestry, Faculty of Engineering, University of Canterbury.
139 Combalicer, M. S., Lee, D. K., Woo, S. Y., Park, P. S., Lee, K. W., Tolentino, E. L., ... & Park, Y. D. (2011). Aboveground biomass and productivity of nitrogen-fixing tree species in the Philippines. Scientific Research and Essays, 6(27), 5820-5836.
140 Constantinides, M., & Fownes, J. H. (1994). Nitrogen mineralization from leaves and litter of tropical plants: relationship to nitrogen, lignin and soluble polyphenol concentrations. Soil Biology and Biochemistry, 26(1), 49-55.
141 Cornelissen, J. H. C., Cerabolini, B., Castro-Díez, P., Villar-Salvador, P., Montserrat-Martí, G., ... & Aerts, R. (2003). Functional traits of woody plants: correspondence of species rankings between field adults and laboratory-grown seedlings? Journal of Vegetation Science, 14(3), 311-322.
142 Côté, B., & Fyles, J. W. (1994). Nutrient concentration and acid–base status of leaf litter of tree species characteristic of the hardwood forest of southern Quebec. Canadian journal of forest research, 24(1), 192-196.
143 Dames, J. F., Scholes, M. C., & Straker, C. J. (2002). Nutrient cycling in a Pinus patula plantation in the Mpumalanga Province, South Africa. Applied soil ecology, 20(3), 211-226.
144 Das, S., & Joy, V. C. (2009). Chemical quality impacts of tropical forest tree leaf litters on the growth and fecundity of soil Collembola. European Journal of Soil Biology, 45(5-6), 448-454.
145 Daubenmire, R., & Prusso, D. (1963). Studies of the decomposition rates of tree litter. Ecology, 44(3), 589-592.
146 De Santo, A. V., De Marco, A., Fierro, A., Berg, B., & Rutigliano, F. A. (2009). Factors regulating litter mass loss and lignin degradation in late decomposition stages. Plant and soil, 318(1-2), 217-228.
147 Dong, T., Li, J., Zhang, Y., Korpelainen, H., Niinemets, Ü., & Li, C. (2015). Partial shading of lateral branches affects growth, and foliage nitrogen-and water-use efficiencies in the conifer Cunninghamia lanceolata growing in a warm monsoon climate. Tree Physiology, 35(6), 632-643.
148 Dong, T., Duan, B., Zhang, S., Korpelainen, H., Niinemets, Ü., & Li, C. (2016). Growth, biomass allocation and photosynthetic responses are related to intensity of root severance and soil moisture conditions in the plantation tree Cunninghamia lanceolata. Tree physiology, 36(7), 807-817.
149 Eguchi, N., Fukatsu, E., Funada, R., Tobita, H., Kitao, M., Maruyama, Y., & Koike, T. (2004). Changes in morphology, anatomy, and photosynthetic capacity of needles of Japanese larch (Larix kaempferi) seedlings grown in high CO2 concentrations. Photosynthetica, 42(2), 173-178.
150 Ehleringer, J. R., Field, C. B., Lin, Z. F., & Kuo, C. Y. (1986). Leaf carbon isotope and mineral composition in subtropical plants along an irradiance cline. Oecologia, 70(4), 520-526.
151 Fahey, T. J. (1983). Nutrient dynamics of aboveground detritus in lodgepole pine (Pinus contorta ssp. latifolia) ecosystems, southeastern Wyoming. Ecological Monographs, 53(1), 51-72.
152 Fellner, H., Dirnberger, G. F., & Sterba, H. (2016). Specific leaf area of European Larch (Larix decidua M ill.). Trees, 30(4), 1237-1244.
153 Frainer, A., Moretti, M. S., Xu, W., & Gessner, M. O. (2015). No evidence for leaf-trait dissimilarity effects on litter decomposition, fungal decomposers, and nutrient dynamics. Ecology, 96(2), 550-561.

154 Ganesh, P. S., Gajalakshmi, S., & Abbasi, S. A. (2009). Vermicomposting of the leaf litter of acacia (Acacia auriculiformis): Possible roles of reactor geometry, polyphenols, and lignin. Bioresource technology, 100(5), 1819-1827.
155 Ghanbary, E., Tabari, M., García-Sánchez, F., Zarafshar, M., & Sanches, M. C. (2012). Response variations of Alnus subcordata (L.), Populus deltoides (Bartr. ex Marsh.), and Taxodium distichum (L.) seedlings to flooding stress. Taiwan Journal of Forest Science, 27(3), 251-263.
156 Gill, R. S., & Lavender, D. P. (1983). Urea fertilization and foliar nutrient composition of Western Hemlock (Tsuga heterophylla (Raf.) Sarc.). Forest ecology and management, 6(4), 333-341.
157 Girisha, G. K., Condron, L. M., Clinton, P. W., & Davis, M. R. (2003). Decomposition and nutrient dynamics of green and freshly fallen radiata pine (Pinus radiata) needles. Forest Ecology and management, 179(1-3), 169-181.
158 Godoy, O., Castro-Díez, P., Van Logtestijn, R. S., Cornelissen, J. H., & Valladares, F. (2010). Leaf litter traits of invasive species slow down decomposition compared to Spanish natives: a broad phylogenetic comparison. Oecologia, 162(3), 781-790.
159 Goudie, J. W., Parish, R., & Antos, J. A. (2016). Foliage biomass and specific leaf area equations at the branch, annual shoot and whole-tree levels for lodgepole pine and white spruce in British Columbia. Forest Ecology and Management, 361, 286-297.
160 Gray, E. F., Wright, I. J., Falster, D. S., Eller, A. S., Lehmann, C. E. R., Bradford, M. G., & Cernusak, L. A. (2019). Leaf: wood allometry and functional traits together explain substantial growth rate variation in rainforest trees. AoB Plants, 11(3), plz024.
161 Guimarães, Z. T. M., Dos Santos, V. A. H. F., Nogueira, W. L. P., de Almeida Martins, N. O., & Ferreira, M. J. (2018). Leaf traits explaining the growth of tree species planted in a Central Amazonian disturbed area. Forest Ecology and Management, 430, 618-628.
162 Guner, S. T., Comez, A. (2017). Biomass equations and changes in carbon stock in afforested black pine (Pinus nigra) stands in Turkey. Fresenius Environmental Bulletin, 26(3), 2368-2379.
163 Guo, L. B., & Sims, R. E. H. (2002). Eucalypt litter decomposition and nutrient release under a short rotation forest regime and effluent irrigation treatments in New Zealand: II. Internal effects. Soil Biology and Biochemistry, 34(7), 913-922.
164 Han, S., LEE, S. J., Yoon, T. K., Han, S. H., Lee, J., Kim, S., ... & Son, Y. (2015). Species-specific growth and photosynthetic responses of first-year seedlings of four coniferous species to open-field experimental warming. Turkish Journal of Agriculture and Forestry, 39(2), 342-349.
165 Hanba, Y. T., Miyazawa, S. I., & Terashima, I. (1999). The influence of leaf thickness on the CO2 transfer conductance and leaf stable carbon isotope ratio for some evergreen tree species in Japanese warm-temperate forests. Functional Ecology, 13(5), 632-639.
166 He, Z., Yu, Z., Huang, Z., Davis, M., & Yang, Y. (2016). Litter decomposition, residue chemistry and microbial community structure under two subtropical forest plantations: A reciprocal litter transplant study. Applied Soil Ecology, 101, 84-92.
167 Isaac, S. R., & Nair, M. A. (2006). Litter dynamics of six multipurpose trees in a homegarden in Southern Kerala, India. Agroforestry Systems, 67(3), 203-213.
168 Ishikawa, H., Osono, T., & Takeda, H. (2007). Effects of clear-cutting on decomposition processes in leaf litter and the nitrogen and lignin dynamics in a temperate secondary forest. Journal of Forest Research, 12(4), 247-254.
169 Jabiol, J., Lecerf, A., Lamothe, S., Gessner, M. O., & Chauvet, E. (2019). Litter quality modulates effects of dissolved nitrogen on leaf decomposition by stream microbial communities. Microbial Ecology, 77(4), 959-966.
170 Jain, T. B., & Graham, R. T. (2015). Decrease in sapling nutrient concentrations for six northern Rocky Mountain coniferous species. Forest Science, 61(3), 570-578.
171 Janusauskaite, D., Baliuckas, V., & Dabkevicius, Z. (2013). Needle litter decomposition of native Pinus sylvestris L. and alien Pinus mugo at different ages affecting enzyme activities and soil properties on dune sands. Baltic Forestry, 19(1), 50-60.
172 Kang, H., Berg, B., Liu, C., & Westman, C. J. (2009). Variation in mass-loss rate of foliar litter in relation to climate and litter quality in Eurasian forests: Differences among functional groups of litter. Silva Fennica, 43(4), 549-575.
173 Khurana, E., & Singh, J. S. (2006). Impact of life-history traits on response of seedlings of five tree species of tropical dry forest to shade. Journal of Tropical Ecology, 653-661.

174 Kizildag, N., Darici, C., & Aka Sagliker, H. (2014). Influence of different parent materials on litter decomposition in the East Mediterranean region. Pak. J. Bot, 46(3), 875-879.
175 Kobe, R. K., & Coates, K. D. (1997). Models of sapling mortality as a function of growth to characterize interspecific variation in shade tolerance of eight tree species of northwestern British Columbia. Canadian Journal of Forest Research, 27(2), 227-236.
176 Kwon, B., Kim, H. S., Jeon, J., & Yi, M. J. (2016). Effects of temporal and interspecific variation of specific leaf area on leaf area index estimation of temperate broadleaved forests in Korea. Forests, 7(10), 215.
177 Lange, O. L., Nobel, P. S., Osmond, C. B., & Ziegler, H. (1983). Encyclopedia of plant physiology. Physiological plant ecology. Responses to the chemical and biological environment. Springer.
178 Leuschner, C., Backes, K., Hertel, D., Schipka, F., Schmitt, U., Terborg, O., & Runge, M. (2001). Drought responses at leaf, stem and fine root levels of competitive Fagus sylvatica L. and Quercus petraea (Matt.) Liebl. trees in dry and wet years. Forest ecology and Management, 149(1-3), 33-46.
179 Leverenz, J. W. (1996). Shade-shoot structure, photosynthetic performance in the field, and photosynthetic capacity of evergreen conifers. Tree Physiology, 16(1-2), 109-114.
180 Li, W., Pan, K. W., Wu, N., Wang, J. C., Han, C. M., & Liang, X. L. (2009). Effects of mixing pine and broadleaved tree/shrub litter on decomposition and N dynamics in laboratory microcosms. Ecological Research, 24(4), 761-769.
181 Li, Q., Zhao, G., Cao, G., & Liu, Z. (2016). Soil effects of six different two-species litter mixtures that include Ulmus pumila. Chemistry and Ecology, 32(8), 707-721.
182 Li, R., Lu, Y., Wan, F., Wang, Y., & Pan, X. (2018). Impacts of a high nitrogen load on foliar nutrient status, N metabolism, and photosynthetic capacity in a Cupressus lusitanica Mill. Plantation. Forests, 9(8), 483.
183 Lienard, J., Florescu, I., & Strigul, N. (2015). An appraisal of the classic forest succession paradigm with the shade tolerance index. PloS One, 10(2), e0117138.
184 Loaiza-Usuga, J. C., León-Peláez, J. D., González-Hernández, M. I., Gallardo-Lancho, J. F., Osorio-Vega, W., & Correa-Londoño, G. (2013). Alterations in litter decomposition patterns in tropical montane forests of Colombia. Canadian Journal of Forest Research, 43(6), 528-533.
185 Loranger, G., Ponge, J. F., Imbert, D., & Lavelle, P. (2002). Leaf decomposition in two semi-evergreen tropical forests: influence of litter quality. Biology and Fertility of Soils, 35(4), 247-252.
186 Maggs, J. (1985). Litter fall and retranslocation of nutrients in a refertilized and prescribed burned Pinus elliottii plantation. Forest ecology and management, 12(3-4), 253-268.
187 Maire, V., Wright, I. J., Prentice, I. C., Batjes, N. H., Bhaskar, R., van Bodegom, P. M., ... & Reich, P. B. (2015). Global effects of soil and climate on leaf photosynthetic traits and rates. Global Ecology and Biogeography, 24(6), 706-717.
188 McArthur, C., Bradshaw, O. S., Jordan, G. J., Clissold, F. J., & Pile, A. J. (2010). Wind affects morphology, function, and chemistry of eucalypt tree seedlings. Int. J. Plant Sci., 171(1), 73-80.
189 McTiernan, K. B., Ineson, P., & Coward, P. A. (1997). Respiration and nutrient release from tree leaf litter mixtures. Oikos, 527-538.
190 Medhurst, J. L., Battaglia, M., Cherry, M. L., Hunt, M. A., White, D. A., & Beadle, C. L. (1999). Allometric relationships for Eucalyptus nitens (Deane and Maiden) Maiden plantations. Trees, 14(2), 91-101.
191 Melillo, J. M., Aber, J. D., & Muratore, J. F. (1982). Nitrogen and lignin control of hardwood leaf litter decomposition dynamics. Ecology, 63(3), 621-626.
192 Merchant, A., Callister, A., Arndt, S., Tausz, M., & Adams, M. (2007). Contrasting physiological responses of six Eucalyptus species to water deficit. Annals of Botany, 100(7), 1507-1515.
193 Moore, T. R., Trofymow, J. A., Prescott, C. E., Titus, B. D., & CIDET Working Group. (2011). Nature and nurture in the dynamics of C, N and P during litter decomposition in Canadian forests. Plant and Soil, 339(1-2), 163-175.
194 Mugendi, D. N., & Nair, P. K. R. (1996). Predicting the decomposition patterns of tree biomass in tropical highland microregions of Kenya. Agroforestry Systems, 35(2), 187-201.

195 Muhammad, S., Wuyts, K., & Samson, R. (2019). Atmospheric net particle accumulation on 96 plant species with contrasting morphological and anatomical leaf characteristics in a common garden experiment. Atmospheric Environment, 202, 328-344.
196 Murphy, K. L., Klopatek, J. M., & Klopatek, C. C. (1998). The effects of litter quality and climate on decomposition along an elevational gradient. Ecological Applications, 8(4), 1061-1071.
197 Nardi, S., Pizzeghello, D., Reniero, F., & Rascio, N. (2000). Chemical and biochemical properties of humic substances isolated from forest soils and plant growth. Soil Science Society of America Journal, 64(2), 639-645.
198 Niinemets, Ü., & Valladares, F. (2006). Tolerance to shade, drought, and waterlogging of temperate Northern Hemisphere trees and shrubs. Ecological Monographs, 76(4), 521-547.
199 Norgrove, L., & Hauser, S. (2000). Leaf properties, litter fall, and nutrient inputs of Terminalia ivorensis at different tree stand densities in a tropical timber-food crop multistrata system. Canadian journal of forest research, 30(9), 1400-1409.
200 Norris, M. D., Blair, J. M., & Johnson, L. C. (2001). Land cover change in eastern Kansas: litter dynamics of closed-canopy eastern redcedar forests in tallgrass prairie. Canadian Journal of Botany, 79(2), 214-222.
201 Ono, K., Hiraide, M., & Amari, M. (2003). Determination of lignin, holocellulose, and organic solvent extractives in fresh leaf, litterfall, and organic material on forest floor using near-infrared reflectance spectroscopy. Journal of Forest Research, 8(3), 191-198.
202 Osono, T., & Takeda, H. (2004). Accumulation and release of nitrogen and phosphorus in relation to lignin decomposition in leaf litter of 14 tree species. Ecological Research, 19(6), 593-602.
203 Ostrofsky, M. L. (1997). Relationship between chemical characteristics of autumn-shed leaves and aquatic processing rates. Journal of the North American Benthological Society, 16(4), 750-759.
204 Paine, C. T., Amissah, L., Auge, H., Baraloto, C., Baruffol, M., Bourland, N., ... & Doust, S. (2015). Globally, functional traits are weak predictors of juvenile tree growth, and we do not know why. Journal of Ecology, 103(4), 978-989.
205 Partey, S. T., Quashie-Sam, S. J., Thevathasan, N. V., & Gordon, A. M. (2011). Decomposition and nutrient release patterns of the leaf biomass of the wild sunflower (Tithonia diversifolia): a comparative study with four leguminous agroforestry species. Agroforestry Systems, 81(2), 123-134.
206 Perala, D. A., & Alban, D. H. (1982). Biomass, nutrient distribution and litterfall in Populus, Pinus and Picea stands on two different soils in Minnesota. Plant and Soil, 64(2), 177-192.
207 Prescott, C. E., Kabzems, R., & Zabek, L. M. (1999). Effects of fertilization on decomposition rate of Populus tremuloides foliar litter in a boreal forest. Canadian Journal of Forest Research, 29(3), 393-397.
208 Prescott, C. E., Vesterdal, L., Pratt, J., Venner, K. H., Montigny, L. D., & Trofymow, J. A. (2000). Nutrient concentrations and nitrogen mineralization in forest floors of single species conifer plantations in coastal British Columbia. Canadian Journal of Forest Research, 30(9), 1341-1352.
209 Purnomo, D., Sulistyo, T. D., & Budiastuti, S. (2012). Potential of varies trees litter containing tannin on agroforestry system as nitrification inhibitor for increasing nitrogen fertilizer efficiency for soybean. Journal of Agricultural Science and Technology. B, 2(2B), 198.
210 Quideau, S. A., Graham, R. C., Oh, S. W., Hendrix, P. F., & Wasylishen, R. E. (2005). Leaf litter decomposition in a chaparral ecosystem, Southern California. Soil Biology and Biochemistry, 37(11), 1988-1998.
211 Radersma, S., Ong, C. K., & Coe, R. (2006). Water use of tree lines: importance of leaf area and micrometeorology in sub-humid Kenya. Agroforestry Systems, 66(3), 179-189.
212 Rahman, M. M., Tsukamoto, J., Rahman, M. M., Yoneyama, A., & Mostafa, K. M. (2013). Lignin and its effects on litter decomposition in forest ecosystems. Chemistry and Ecology, 29(6), 540-553.
213 Rahmonov, O. (2009). The chemical composition of plant litter of black locust (Robinia pseudoacacia L.) and its ecological role in sandy ecosystems. Acta Ecologica Sinica, 29(4), 237-243.

214 Reich, P. B., Tjoelker, M. G., Walters, M. B., Vanderklein, D. W., & Buschena, C. (1998). Close association of RGR, leaf and root morphology, seed mass and shade tolerance in seedlings of nine boreal tree species grown in high and low light. Functional Ecology, 12(3), 327-338.
215 Ribeiro, C., Madeira, M., & Araújo, M. C. (2002). Decomposition and nutrient release from leaf litter of Eucalyptus globulus grown under different water and nutrient regimes. Forest Ecology and Management, 171(1-2), 31-41.
216 Roberts, B. A., Deering, K. W., & Titus, B. D. (1998). Effects of intensive harvesting on forest floor properties in Betula papyrifera stands in Newfoundland. Journal of Vegetation Science, 9(4), 521-528.
217 Rodriguez-Calcerrada, J., Reich, P. B., Rosenqvist, E., Pardos, J. A., Cano, F. J., & Aranda, I. (2008). Leaf physiological versus morphological acclimation to high-light exposure at different stages of foliar development in oak. Tree physiology, 28(5), 761-771.
218 Roggy, J. C., Moiroud, A., Lensi, R., & Domenach, A. M. (2004). Estimating N transfers between N2-fixing actinorhizal species and the non-N2-fixing Prunus avium under partially controlled conditions. Biology and fertility of soils, 39(5), 312-319.
219 Rueda, M., Godoy, O., & Hawkins, B. A. (2018). Trait syndromes among North American trees are evolutionarily conserved and show adaptive value over broad geographic scales. Ecography, 41(3), 540-550.
220 Salamanca, E. F., Kaneko, N., & Katagiri, S. (1998). Effects of leaf litter mixtures on the decomposition of Quercus serrata and Pinus densiflora using field and laboratory microcosm methods. Ecological Engineering, 10(1), 53-73.
221 Santonja, M., Fernandez, C., Gauquelin, T., & Baldy, V. (2015). Climate change effects on litter decomposition: intensive drought leads to a strong decrease of litter mixture interactions. Plant and Soil, 393(1-2), 69-82.
222 Saur, E., Nambiar, E. K. S., & Fife, D. N. (2000). Foliar nutrient retranslocation in Eucalyptus globulus. Tree physiology, 20(16), 1105-1112.
223 Sausen, T. L., Schaefer, G. F. D. P., Tomazi, M., Santos, L. S. D., Bayer, C., & Rosa, L. M. G. (2014). Clay content drives carbon stocks in soils under a plantation of Eucalyptus saligna Labill. in southern Brazil. Acta Botanica Brasilica, 28(2), 266-273.
224 Schlesinger, W. H., & Hasey, M. M. (1981). Decomposition of chaparral shrub foliage: losses of organic and inorganic constituents from deciduous and evergreen leaves. Ecology, 62(3), 762-774.
225 Schortemeyer, M., Atkin, O. K., McFarlane, N., & Evans, J. R. (2002). N2 fixation by Acacia species increases under elevated atmospheric CO2. Plant, Cell & Environment, 25(4), 567-579.
226 Segura, C., Fernández-Ondoño, E., Jiménez, M. N., & Navarro, F. B. (2019). Carbon and nutrient contents in the miscellaneous fraction of litterfall under different thinning intensities in a semiarid Pinus halepensis afforestation. iForest-Biogeosciences and Forestry, 12(4), 375.
227 Semwal, R. L., Maikhuri, R. K., Rao, K. S., Sen, K. K., & Saxena, K. G. (2003). Leaf litter decomposition and nutrient release patterns of six multipurpose tree species of central Himalaya, India. Biomass and Bioenergy, 24(1), 3-11.
228 Serrano, L., Penuelas, J., & Ustin, S. L. (2002). Remote sensing of nitrogen and lignin in Mediterranean vegetation from AVIRIS data: Decomposing biochemical from structural signals. Remote sensing of Environment, 81(2-3), 355-364.
229 Shahrokhzadeh, U., Sohrabi, H., & Copenheaver, C. A. (2015). Aboveground biomass and leaf area equations for three common tree species of Hyrcanian temperate forests in northern Iran. Botany, 93(10), 663-670.
230 Sharpe, D. M., Cromack Jr, K., Johnson, W. C., & Ausmuss, B. S. (1980). A regional approach to litter dynamics in Southern Appalachian forests. Canadian Journal of Forest Research, 10(3), 395-404.
231 Singh, K. P., Singh, P. K., & Tripathi, S. K. (1999). Litterfall, litter decomposition and nutrient release patterns in four native tree species raised on coal mine spoil at Singrauli, India. Biology and Fertility of Soils, 29(4), 371-378.
232 Smith, C. K., Gholz, H. L., & de Assis Oliveira, F. (1998). Fine litter chemistry, early-stage decay, and nitrogen dynamics under plantations and primary forest in lowland Amazonia. Soil Biology and Biochemistry, 30(14), 2159-2169.

233 Spain, A. V., & Le Feuvre, R. P. (1987). Breakdown of four litters of contrasting quality in a tropical Australian rainforest. Journal of Applied Ecology, 279-288.
234 Sudrajat, D. J., Siregar, I. Z., Khumaida, N., Siregar, U. J., & Mansur, I. (2015). Adaptability of white jabon (Anthocephalus cadamba MIQ.) seedling from 12 populations to drought and waterlogging. Agrivita, Journal of Agricultural Science, 37(2), 130-143.
235 Tabari, M., Yosef-Zadeh, H., Espahbodi, K., & Jalali, G. A. (2008). The effect of seed source on the leaf morphology of Acer velutinum (Boiss.) seedlings. Taiwan J For Sci, 23(1), 13-9.
236 Tanikawa, N., Nakaji, T., Yahara, H., & Makita, N. (2019). Exploring patterns of fine root morphological, chemical, and anatomical traits of 12 tree species from visible–near-infrared spectral reflectance. Plant and Soil, 445(1-2), 469-481.
237 Tolia, N., Devakumar, A. S., Sheshshayee, M. S., & Kambalimath, S. (2019). Growth performance of six multipurpose tree species based on the carbon assimilation capacity: a functional approach. Agroforestry Systems, 93(3), 1031-1043.
238 Turner, J., & Lambert, M. J. (1983). Nutrient cycling within a 27-year-old Eucalyptus grandis plantation in New South Wales. Forest Ecology and Management, 6(2), 155-168.
239 van Huysen, T. L., Harmon, M. E., Perakis, S. S., & Chen, H. (2013). Decomposition and nitrogen dynamics of 15N-labeled leaf, root, and twig litter in temperate coniferous forests. Oecologia, 173(4), 1563-1573.
240 Vityakon, P., & Dangthaisong, N. (2005). Environmental influences on nitrogen transformation of different quality tree litter under submerged and aerobic conditions. Agroforestry Systems, 63(3), 225-236.
241 Wan, X., Huang, Z., He, Z., Yu, Z., Wang, M., Davis, M. R., & Yang, Y. (2015). Soil C: N ratio is the major determinant of soil microbial community structure in subtropical coniferous and broadleaf forest plantations. Plant and soil, 387(1-2), 103-116.
242 Wang, Q. K., Wang, S. L., & Zhong, M. C. (2013). Ecosystem carbon storage and soil organic carbon stability in pure and mixed stands of Cunninghamia lanceolata and Michelia macclurei. Plant and Soil, 370(1-2), 295-304.
243 Wang, N., Palmroth, S., Maier, C. A., Domec, J. C., & Oren, R. (2019). Anatomical changes with needle length are correlated with leaf structural and physiological traits across five Pinus species. Plant, Cell & Environment, 42(5), 1690-1704.
244 Weger, H. G., Silim, S. N., & Guy, R. D. (1993). Photosynthetic acclimation to low temperature by western red cedar seedlings. Plant, Cell & Environment, 16(6), 711-717.
245 White, J. D., & Scott, N. A. (2006). Specific leaf area and nitrogen distribution in New Zealand forests: Species independently respond to intercepted light. Forest Ecology and Management, 226(1-3), 319-329.
246 Wooliver, R. C., Marion, Z. H., Peterson, C. R., Potts, B. M., Senior, J. K., Bailey, J. K., & Schweitzer, J. A. (2017). Phylogeny is a powerful tool for predicting plant biomass responses to nitrogen enrichment. Ecology, 98(8), 2120-2132.
247 Wu, H., Xiang, W., Fang, X., Lei, P., Ouyang, S., & Deng, X. (2017). Tree functional types simplify forest carbon stock estimates induced by carbon concentration variations among species in a subtropical area. Scientific Reports, 7(1), 1-11.
248 Xue, L. (1996). Nutrient cycling in a Chinese-fir (Cunninghamia lanceolata) stand on a poor site in Yishan, Guangxi. Forest Ecology and Management, 89(1-3), 115-123.
249 Yadav, R. S., Yadav, B. L., & Chhipa, B. R. (2008). Litter dynamics and soil properties under different tree species in a semi-arid region of Rajasthan, India. Agroforestry Systems, 73(1), 1-12.
250 Yan, X. L., Wang, C., Ma, X., & Wu, P. (2019). Root morphology and seedling growth of three tree species in southern China in response to homogeneous and heterogeneous phosphorus supplies. Trees, 33(5), 1283-1297.
251 Yang, Y. S., Guo, J. F., Chen, G. S., Xie, J. S., Cai, L. P., & Lin, P. (2004). Litterfall, nutrient return, and leaf-litter decomposition in four plantations compared with a natural forest in subtropical China. Annals of Forest Science, 61(5), 465-476.
252 Yang, K., & Zhu, J. J. (2015). Impact of tree litter decomposition on soil biochemical properties obtained from a temperate secondary forest in Northeast China. Journal of Soils and Sediments, 15(1), 13-23.

253 Zhao, L., Hu, Y. L., Lin, G. G., Gao, Y. C., Fang, Y. T., & Zeng, D. H. (2013). Mixing effects of understory plant litter on decomposition and nutrient release of tree litter in two plantations in Northeast China. PLoS One, 8(10), e76334.
254 Zhang, X., & Wang, W. (2015). The decomposition of fine and coarse roots: their global patterns and controlling factors. Scientific Reports, 5, 9940.
255 Zhang, Q., Xiong, D., Xie, J., Li, X., You, Z., Lyu, M., ... & Yang, Y. (2018). Ecophysiological process regulates the growth of Cunninghamia lanceolata to suit short-term warming and nitrogen addition in the sub-tropical regions. Trees, 32(2), 631-643.
256 Zhu, X., Chen, H., Zhang, W., Huang, J., Fu, S., Liu, Z., & Mo, J. (2016). Effects of nitrogen addition on litter decomposition and nutrient release in two tropical plantations with N2-fixing vs. non-N2-fixing tree species. Plant and Soil, 399(1-2), 61-74.
257 Zimmermann, T. G., Andrade, A., & Richardson, D. M. (2016). Experimental assessment of factors mediating the naturalization of a globally invasive tree on sandy coastal plains: a case study from Brazil. AoB Plants, 8.
258 Zukswert, J. M., & Prescott, C. E. (2017). Relationships among leaf functional traits, litter traits, and mass loss during early phases of leaf litter decomposition in 12 woody plant species. Oecologia, 185(2), 305-316.

Supplementary Reference 3 – List of references used to build the mixed forests dataset and the SOC stability dataset

Supplementary Reference 3a – Mixed forests dataset

259 Andivia, E., Rolo, V., Jonard, M., Formánek, P., & Ponette, Q. (2016). Tree species identity mediates mechanisms of top soil carbon sequestration in a Norway spruce and European beech mixed forest. Annals of forest science, 73(2), 437-447.

260 Berger, T. W., Inselsbacher, E., & Zechmeister-Boltenstern, S. (2010). Carbon dioxide emissions of soils under pure and mixed stands of beech and spruce, affected by decomposing foliage litter mixtures. Soil Biology and Biochemistry, 42(6), 986-997.

261 Blaško, R., Forsmark, B., Gundale, M. J., Lundmark, T., & Nordin, A. (2020). Impacts of tree species identity and species mixing on ecosystem carbon and nitrogen stocks in a boreal forest. Forest Ecology and Management, 458, 117783.

262 Chomel, M., DesRochers, A., Baldy, V., Larchevêque, M., & Gauquelin, T. (2014). Non-additive effects of mixing hybrid poplar and white spruce on aboveground and soil carbon storage in boreal plantations. Forest ecology and management, 328, 292-299.

263 Cremer, M., & Prietzel, J. (2017). Soil acidity and exchangeable base cation stocks under pure and mixed stands of European beech, Douglas fir and Norway spruce. Plant and Soil, 415(1-2), 393-405.

264 Díaz-Pinés, E., Rubio, A., Van Miegroet, H., Montes, F., & Benito, M. (2011). Does tree species composition control soil organic carbon pools in Mediterranean mountain forests?. Forest Ecology and Management, 262(10), 1895-1904.

265 Fanin, N., Maxwell, T. L., Altinalmazis-Kondylis, A., Bon, L., Meredieu, C., Jactel, H., Bakker, M. R., Augusto, L. (2021). Effects of tree species mixture and water availability on soil organic carbon stocks are depth-dependent in a temperate podzol. Submitted.

266 Forrester, D. I., Bauhus, J., & Cowie, A. L. (2005). On the success and failure of mixed-species tree plantations: lessons learned from a model system of Eucalyptus globulus and Acacia mearnsii. Forest Ecology and Management, 209(1-2), 147-155.

267 Jiang, Y. M., Chen, C. R., Liu, Y. Q., & Xu, Z. H. (2010). Soil soluble organic carbon and nitrogen pools under mono-and mixed species forest ecosystems in subtropical China. Journal of Soils and Sediments, 10(6), 1071-1081.

268 Kaye, J. P., Resh, S. C., Kaye, M. W., & Chimner, R. A. (2000). Nutrient and carbon dynamics in a replacement series of Eucalyptus and Albizia trees. Ecology, 81(12), 3267-3273.

269 Laganière, J., Angers, D. A., Paré, D., Bergeron, Y., & Chen, H. Y. (2011). Black spruce soils accumulate more uncomplexed organic matter than aspen soils. Soil Science Society of America Journal, 75(3), 1125-1132.

270 Laganiere, J., Paré, D., Bergeron, Y., Chen, H. Y., Brassard, B. W., & Cavard, X. (2013). Stability of soil carbon stocks varies with forest composition in the Canadian boreal biome. Ecosystems, 16(5), 852-865.

271 Langenbruch, C., Helfrich, M., & Flessa, H. (2012). Effects of beech (Fagus sylvatica), ash (Fraxinus excelsior) and lime (Tilia spec.) on soil chemical properties in a mixed deciduous forest. Plant and Soil, 352(1-2), 389-403.

272 Lee, S. K., Son, Y., Noh, N. J., Heo, S. J., Yoon, T. K., Lee, A. R., ... & Lee, W. K. (2009). Carbon storage of natural pine and oak pure and mixed forests in Hoengseong, Kangwon. Journal of Korean Society of Forest Science, 98(6), 772-779.

273 Lemma, B. (2012). Soil chemical properties and nutritional status of trees in pure and mixed-species stands in south Ethiopia. Journal of Plant Nutrition and Soil Science, 175(5), 769-774.

274 López-Marcos, D., Martínez-Ruiz, C., Turrión, M. B., Jonard, M., Titeux, H., Ponette, Q., & Bravo, F. (2018). Soil carbon stocks and exchangeable cations in monospecific and mixed pine forests. European Journal of Forest Research, 137(6), 831-847.

275 Matos, E. S., Freese, D., Ślązak, A., Bachmann, U., Veste, M., & Hüttl, R. F. (2010). Organic-carbon and nitrogen stocks and organic-carbon fractions in soil under mixed pine and oak forest stands of different ages in NE Germany. Journal of Plant Nutrition and Soil Science, 173(5), 654-661.

276 Mellor, N. J., Hellerich, J., Drijber, R., Morris, S. J., Stromberger, M. E., & Paul, E. A. (2013). Changes in ecosystem carbon following afforestation of native sand prairie. Soil Science Society of America Journal, 77(5), 1613-1624.
277 Wen, L., Lei, P., Xiang, W., Yan, W., & Liu, S. (2014). Soil microbial biomass carbon and nitrogen in pure and mixed stands of Pinus massoniana and Cinnamomum camphora differing in stand age. Forest Ecology and Management, 328, 150-158.
278 Yan, W. D., Xu, W. M., Chen, X. Y., Tian, D. L., Peng, Y. Y., Zhen, W., ... & Xu, J. (2014). Soil CO2 flux in different types of forests under a subtropical microclimatic environment. Pedosphere, 24(2), 243-250.

Supplementary Reference 3b – SOC stability dataset

279 Boča, A., & Van Miegroet, H. (2017). Can carbon fluxes explain differences in soil organic carbon storage under aspen and conifer forest overstories?. Forests, 8(4), 118.
280 Charro, E., Gallardo, J. F., & Moyano, A. (2010). Degradability of soils under oak and pine in Central Spain. European journal of forest research, 129(1), 83-91.
281 Chen, G. S., Yang, Y. S., Xie, J. S., Guo, J. F., Gao, R., & Qian, W. (2005). Conversion of a natural broad-leafed evergreen forest into pure plantation forests in a subtropical area: effects on carbon storage. Annals of forest science, 62(7), 659-668.
282 Díaz-Pinés, E., Rubio, A., Van Miegroet, H., Montes, F., & Benito, M. (2011). Does tree species composition control soil organic carbon pools in Mediterranean mountain forests?. Forest Ecology and Management, 262(10), 1895-1904.
283 Forrester, D. I., Bauhus, J., & Cowie, A. L. (2005). On the success and failure of mixed-species tree plantations: lessons learned from a model system of Eucalyptus globulus and Acacia mearnsii. Forest Ecology and Management, 209(1-2), 147-155.
284 Forrester, D. I., Pares, A., O'hara, C., Khanna, P. K., & Bauhus, J. (2013). Soil organic carbon is increased in mixed-species plantations of Eucalyptus and nitrogen-fixing Acacia. Ecosystems, 16(1), 123-132.
285 Graham, R. C., Ervin, J. O., & Wood, H. B. (1995). Aggregate stability under oak and pine after four decades of soil development. Soil Science Society of America Journal, 59(6), 1740-1744.
286 Huang, Z., Davis, M. R., Condron, L. M., & Clinton, P. W. (2011). Soil carbon pools, plant biomarkers and mean carbon residence time after afforestation of grassland with three tree species. Soil Biology and Biochemistry, 43(6), 1341-1349.
287 Jiang, P. K., & Qiu-Fang, X. U. (2006). Abundance and dynamics of soil labile carbon pools under different types of forest vegetation. Pedosphere, 16(4), 505-511.
288 Johnsen, K. H., Samuelson, L. J., Sanchez, F. G., & Eaton, R. J. (2013). Soil carbon and nitrogen content and stabilization in mid-rotation, intensively managed sweetgum and loblolly pine stands. Forest ecology and management, 302, 144-153.
289 Kasel, S., Singh, S., Sanders, G. J., & Bennett, L. T. (2011). Species-specific effects of native trees on soil organic carbon in biodiverse plantings across north-central Victoria, Australia. Geoderma, 161(1-2), 95-106.
290 Ladegaard-Pedersen, P., Elberling, B., & Vesterdal, L. (2005). Soil carbon stocks, mineralization rates, and CO2 effluxes under 10 tree species on contrasting soil types. Canadian Journal of Forest Research, 35(6), 1277-1284.
291 Landgraf, D., Wedig, S., & Klose, S. (2005). Medium-and short-term available organic matter, microbial biomass, and enzyme activities in soils under Pinus sylvestris L. and Robinia pseudoacacia L. in a sandy soil in NE Saxony, Germany. Journal of Plant Nutrition and Soil Science, 168(2), 193-201.
292 Laganière, J., Angers, D. A., Paré, D., Bergeron, Y., & Chen, H. Y. (2011). Black spruce soils accumulate more uncomplexed organic matter than aspen soils. Soil Science Society of America Journal, 75(3), 1125-1132.
293 Laganiere, J., Paré, D., Bergeron, Y., Chen, H. Y., Brassard, B. W., & Cavard, X. (2013). Stability of soil carbon stocks varies with forest composition in the Canadian boreal biome. Ecosystems, 16(5), 852-865.

294 Laik, R., Kumar, K., Das, D. K., & Chaturvedi, O. P. (2009). Labile soil organic matter pools in a calciorthent after 18 years of afforestation by different plantations. Applied Soil Ecology, 42(2), 71-78.
295 Matos, E. S., Freese, D., Ślązak, A., Bachmann, U., Veste, M., & Hüttl, R. F. (2010). Organic-carbon and nitrogen stocks and organic-carbon fractions in soil under mixed pine and oak forest stands of different ages in NE Germany. Journal of Plant Nutrition and Soil Science, 173(5), 654-661.
296 McFarland, J. W., Ruess, R. W., Kielland, K., Pregitzer, K., & Hendrick, R. (2010). Glycine mineralization in situ closely correlates with soil carbon availability across six North American forest ecosystems. Biogeochemistry, 99(1-3), 175-191.
297 Menyailo, O. V., & Hungate, B. A. (2005). Tree species effects on potential production and consumption of carbon dioxide, methane, and nitrous oxide: the Siberian afforestation experiment. In Tree Species Effects on Soils: Implications for Global Change (pp. 293-305). Springer, Dordrecht.
298 Ramesh, T., Manjaiah, K. M., Tomar, J. M. S., & Ngachan, S. V. (2013). Effect of multipurpose tree species on soil fertility and CO2 efflux under hilly ecosystems of Northeast India. Agroforestry systems, 87(6), 1377-1388.
299 Riestra, D., Noellemeyer, E., & Quiroga, A. (2012). Soil texture and forest species condition the effect of afforestation on soil quality parameters. Soil science, 177(4), 279-287.
300 Suominen, K., Kitunen, V., & Smolander, A. (2003). Characteristics of dissolved organic matter and phenolic compounds in forest soils under silver birch (Betula pendula), Norway spruce (Picea abies) and Scots pine (Pinus sylvestris). European journal of soil science, 54(2), 287-293.
301 Susyan, E. A., Ananyeva, N. D., Gavrilenko, E. G., Chernova, O. V., & Bobrovskii, M. V. (2009). Microbial biomass carbon in the profiles of forest soils of the southern taiga zone. Eurasian Soil Science, 42(10), 1148-1155.
302 Tang, G., & Li, K. (2013). Tree species controls on soil carbon sequestration and carbon stability following 20 years of afforestation in a valley-type savanna. Forest Ecology and Management, 291, 13-19.
303 Wang, Q., Xiao, F., Zhang, F., & Wang, S. (2013). Labile soil organic carbon and microbial activity in three subtropical plantations. Forestry, 86(5), 569-574.
