# Supplementary material for: Tree functional traits, forest biomass, and tree species diversity interact with site properties to drive forest soil carbon
Source: Nat Commun. 2022 Mar 1;13:1097. doi: 10.1038/s41467-022-28748-0 (PMC8888738; doi:10.1038/s41467-022-28748-0)
Supplement: Supplementary file 2 — Reporting Summary [file 41467_2022_28748_MOESM2_ESM.pdf]

nature research

Corresponding author(s): Laurent Augusto

Last updated by author(s): 2022/01/30

Reporting Summary

Nature Research wishes to improve the reproducibility of the work that we publish. This form provides structure for consistency and transparency in reporting. For further information on Nature Research policies, see our Editorial Policies and the Editorial Policy Checklist.

Statistics

For all statistical analyses, confirm that the following items are present in the figure legend, table legend, main text, or Methods section.

n/a	Confirmed	
☐	☒	The exact sample size (*n*) for each experimental group/condition, given as a discrete number and unit of measurement
☐	☒	A statement on whether measurements were taken from distinct samples or whether the same sample was measured repeatedly
☐	☒	The statistical test(s) used AND whether they are one- or two-sided<br>*Only common tests should be described solely by name; describe more complex techniques in the Methods section.*
☐	☒	A description of all covariates tested
☐	☒	A description of any assumptions or corrections, such as tests of normality and adjustment for multiple comparisons
☐	☒	A full description of the statistical parameters including central tendency (e.g. means) or other basic estimates (e.g. regression coefficient) AND variation (e.g. standard deviation) or associated estimates of uncertainty (e.g. confidence intervals)
☐	☒	For null hypothesis testing, the test statistic (e.g. *F*, *t*, *r*) with confidence intervals, effect sizes, degrees of freedom and *P* value noted<br>*Give P values as exact values whenever suitable.*
☒	☐	For Bayesian analysis, information on the choice of priors and Markov chain Monte Carlo settings
☒	☐	For hierarchical and complex designs, identification of the appropriate level for tests and full reporting of outcomes
☒	☐	Estimates of effect sizes (e.g. Cohen's *d*, Pearson's *r*), indicating how they were calculated

Our web collection on statistics for biologists contains articles on many of the points above.

Software and code

Policy information about availability of computer code

Data collection	Data was collected following methods used in compilation studies and meta-analyses (searches with Web of Science, and Google Scholar).<br>SOC data were collected from original publications (when necessary with WebPlotDigitizer version 4.3: https://automeris.io/WebPlotDigitizer).<br>Data about plant traits were obtained from databases (TRY, FRED, China Plant Trait database, ORNL DAAC leaf traits, and TTT), publications (see Supplementary References) and websites (https://www.wikipedia.org, https://www.conifers.org, http://efloras.org, http://www.fs.fed.us, https://www.gbif.org, http://issg.org/database/welcome/, www.iplantz.com, https://pfaf.org, http://www.tree-guide.com, http://tropical.theferns.info, https://wiki.bugwood.org/Main_Page, http://www.worldagroforestry.org, http://data.kew.org/sid, http://db.worldagroforestry.org/wd). Elevation and climatic variables were collected from the publications and global databases (http://worldclim.org, https://cgiarcsi.community, https://www2.jpl.nasa.gov/srtm).<br>All data were harmonised and normalised (with code developped by authors using SAS (version 9.4) and R (version 4.0.3) languages).
Data analysis	Data were analysed using common statistical methods (random forest, linear regressions, mixed models, Bonferroni test, Mann-Whitney test) using code developped by authors (SAS and R languages; respectively versions 9.4 and 4.0.3). All analyses are fully described in the Methods section. Codes are available upon requests to authors.

For manuscripts utilizing custom algorithms or software that are central to the research but not yet described in published literature, software must be made available to editors and reviewers. We strongly encourage code deposition in a community repository (e.g. GitHub). See the Nature Research guidelines for submitting code & software for further information.

Data

Policy information about availability of data

All manuscripts must include a data availability statement. This statement should provide the following information, where applicable:

- Accession codes, unique identifiers, or web links for publicly available datasets
- A list of figures that have associated raw data
- A description of any restrictions on data availability

Statements of data and code availability are available online.
The main SOC dataset generated in this study have been deposited in the https://data.inrae.fr/ database under accession link https://doi.org/10.15454/LJRFJR (used for the main Figures). The references used to compile SOC data and plant traits are provided in the Supplementary Information. The raw from TRY database are protected and are not available due to data privacy laws. However, raw data for plant traits can be obtained directly from the TRY database (www.try-db.org).

Field-specific reporting

Please select the one below that is the best fit for your research. If you are not sure, read the appropriate sections before making your selection.

☐ Life sciences ☐ Behavioural & social sciences ☒ Ecological, evolutionary & environmental sciences

For a reference copy of the document with all sections, see nature.com/documents/nr-reporting-summary-flat.pdf

Ecological, evolutionary & environmental sciences study design

All studies must disclose on these points even when the disclosure is negative.

Study description	We evaluated to what extent the tree properties (known as functional traits) can explain the quantity and the stability of soil organic carbon (SOC) in forests.
Research sample	We conducted a global analysis on all common gardens that comprised several mature (> 10 years) mono-specific stands of trees. In order to have a dataset representative of all taxa, no a priori criterion was applied to select tree species. Data was extracted from published studies (see Data collection). The final data selection was composed of 454 mature and mono-specific forest stands distributed worldwide. Stands were clustered into 136 sites that contained several mono-specific stands in comparable conditions, enabling us to compare the influence of different tree species on SOC pools. In total, 178 tree species, from 35 families, were represented.
Sampling strategy	We explored the literature using specific queries on scientific platforms (Web of Science, Google Scholar). In addition, we took into account former review studies (see PRISMA data flow). All proposed references were evaluated by both authors. The publications that met the inclusion criteria (see Methods) we retained. As in most compilation studies, we did not determine a priori any sample size. Instead, we collected as much data as possible.
Data collection	The data extraction/checking/homogenisation from the publication was done by the two authors, who are experienced in soil science. Data were directly collected from tables, or appendices, in publications. When data was presented as figures, we used a dedicated software to estimate the original values (WebPlotDigitizer version 4.3). Data were completely collected and handled by the two authors (no sub-contracting, nor student internship). The two authors had regular collective working sessions to cross-check the quality of their respective work. The procedure for collection is fully explained in the Methods section.
Timing and spatial scale	Data collection for SOC values was carried out from October 2017 (beginning of the study) to December 2018 (date at which all relevant data was extracted from the selected publications: see Data collection).<br>Data collection for plant traits was initiated in October 2019 (just after consolidation of the data about SOC) and was closed in October 2020 (when we found no more usable values in the literature or open databases).<br>To enable being representative of all kinds of forest, no restriction was used in terms of spatial scale.
Data exclusions	There was no data exclusion for functional traits. SOC data about non-forest sites, or highly disturbed reforested sites (mining spoil heaps, harbour sediments, city parks, etc.) were not retained.
Reproducibility	The results can be reproduced as all the bibliographic requests, the selected publications, the data retained, and all the data handling are fully described in the Methods of the article. In addition, the core dataset of the study is available online (see Data availability in the manuscript). The numerous code scripts (in SAS or in R, depending on the author and analysis) are available from the corresponding authors upon request.
Randomization	Because we retained all relevant data that was found, it was not possible to randomise data.
Blinding	Blinding was not relevant because data collection for meta-analyses requires experienced operators. Instead, the two authors (who are both experienced in such studies) worked together, discussing systematically the relevance of retaining (or not) each dataset.

Did the study involve field work? ☐ Yes ☒ No

Reporting for specific materials, systems and methods

We require information from authors about some types of materials, experimental systems and methods used in many studies. Here, indicate whether each material, system or method listed is relevant to your study. If you are not sure if a list item applies to your research, read the appropriate section before selecting a response.

Materials & experimental systems

n/a	Involved in the study
☒	☐ Antibodies
☒	☐ Eukaryotic cell lines
☒	☐ Palaeontology and archaeology
☒	☐ Animals and other organisms
☒	☐ Human research participants
☒	☐ Clinical data
☒	☐ Dual use research of concern

Methods

n/a	Involved in the study
☒	☐ ChIP-seq
☒	☐ Flow cytometry
☒	☐ MRI-based neuroimaging
